# How to break free from the "tool" dilemma? A study on the impact of perceived algorithmic control on sustainable value co-creation behavior

**DOI:** 10.1371/journal.pone.0357097

**Published:** 2026-09-08

**Authors:** Xuan Liu, Yuqing Wang, Haowei Zheng, Yuci Chen

**Affiliations:** School of Economics and Management, Nanjing Tech University, Nanjing, China; Longgang Otorhinolaryngology Hospital & Shenzhen Key Laboratory of Otorhinolaryngology, Shenzhen Institute of Otorhinolaryngology, CHINA

## Abstract

With the growing prevalence of algorithmic technologies, algorithmic management has become a dominant control mechanism in the gig economy, profoundly shaping platform workers’ psychological states and behaviors. Grounded in objectification theory and trait activation theory, this study examines how perceived algorithmic control (PAC) affects platform workers’ sustainable value co-creation behavior (SVCB), highlighting the mediating role of workplace objectification (WO) and the moderating role of algorithm aversion (AAV). Using a two-wave survey (n = 285) and a scenario-based experiment (PAC experimental vs. control group; n = 216), the study finds that: (1) PAC significantly negatively affects SVCB; (2) PAC increases WO, which in turn reduces SVCB; (3) AAV positively moderates the indirect effect of PAC on SVCB through WO. This study advances understanding of the psychological mechanisms linking PAC to SVCB in gig work settings. It provides actionable insights for enhancing algorithmic governance and fostering sustained engagement among platform workers.

## Introduction

With the rapid growth of mobile internet and the gig economy, online labor platforms (OLPs), such as Uber in the US and Meituan in China—have flourished. These platforms use algorithmic technologies to match fragmented user demands with workers’ spare time, reshaping traditional labor structures [1]. This has created a new labor force—platform workers—who perform tasks such as food delivery or ride-hailing under algorithmic control [2], such as food delivery riders and ride-hailing drivers. The penetration of platform-based work has been expanding and now constitutes a significant component of global employment [3]. In China, the Ali Research Institute predicts that by 2036, over 400 million people will engage in gig economy activities [4]. A similar growth trend is also evident across Western economies, where labor participation via platforms continues to rise [5,6]. Unlike traditional employment relationships, platform workers typically maintain a mutually beneficial, transactional relationship with the platform [7]. The long-term sustainability of platform enterprises depends heavily on the continued engagement and value creation of these workers [8].

Sustainable value co-creation behavior (SVCB) refers to employees’ proactive, ongoing engagement in value co-creation with their organization [9]. Within the context of the gig economy, SVCB is manifested in platform workers’ use of their skills, time, and resources to provide services to users, thereby enhancing service quality and brand value in collaboration with the platform [10]. For platforms, SVCB contributes to improved operational efficiency, enhanced user experience, and sustained competitive advantage [11,12]. For platform workers themselves, SVCB fosters job satisfaction and occupational identity, while also increasing access to orders, customer ratings, and platform-based rewards, ultimately enhancing income stability [9]. Therefore, how to effectively motivate SVCB among platform workers has become a critical concern for platform enterprises.

Unlike traditional organizations that use technical regulations and supervision, OLPs rely on algorithmic control. Algorithmic control automates task allocation, performance monitoring, and incentive adjustments [13]. While enhancing efficiency, algorithmic control can undermine workers’ autonomy and intrinsic motivation [14,15], reducing engagement and increasing psychological stress [16]. More broadly, when digitalized and intelligent technologies intervene in work processes, individuals’ attitudes, intentions, and perceived control can influence their behavioral responses to technological change [17]. In the context of platform work, recent research further suggests that PAC can affect gig workers’ work engagement, potentially through mechanisms such as psychological empowerment [18] However, existing studies have primarily focused on outcomes such as work engagement, service performance, or general work attitudes, while insufficient attention has been paid to whether PAC affects platform workers’ sustainable, cooperative, and relational value co-creation behavior, as well as the psychological mechanisms underlying this relationship. Since the long-term success of OLPs depends on sustained worker contribution, it is crucial to understand how PAC influences SVCB.

Objectification Theory suggests that individuals may be treated as objects or tools in specific social contexts, rather than as whole persons with autonomy, emotions, and subjectivity [19]. This theory was originally developed mainly to explain sexual objectification [20], but in recent years it has gradually been extended to workplace research, giving rise to the concept of workplace objectification (WO). WO concerns individuals’ instrumentalized position in workplace relations and their subjective experience of this position. Specifically, it refers to the extent to which individuals feel that they are reduced to instrumental means for achieving organizational goals, rather than being recognized as subjects with independent value and autonomy. At its core, WO captures workers’ experience of being treated as replaceable, controllable, and autonomy-constrained productive resources in the labor process [21,22].

When explaining the negative psychological experiences of platform workers under algorithmic management, WO and organizational dehumanization are two related but distinct concepts, both of which involve the experience of workers not being treated as whole subjects. To clarify the theoretical focus of the present study, it is necessary to briefly distinguish between them here. Organizational dehumanization places greater emphasis on employees’ external evaluation of the organization or management system, that is, employees feel that the organization denies their human attributes [23,24]. WO, by contrast, focuses more on instrumentalized self-understanding in labor relations, that is, whether employees feel that they are merely replaceable, usable, and controllable functional means within organizational or platform operations. In the context of the gig economy, WO is particularly explanatory. Platforms organize the labor process through algorithmic management practices such as data monitoring, automated dispatching, performance rating, and reward and punishment feedback [1], which makes platform workers more likely to perceive themselves as merely instrumental resources for completing tasks and maintaining system operations. Therefore, focusing on the research context of this study, WO is better able to capture platform workers’ perceptions of instrumentality and replaceability under algorithmic control. Previous studies have shown that WO weakens workers’ organizational identification, willingness to cooperate, and proactive engagement [25,26]. Based on this, the present study proposes that WO may be an important psychological mechanism through which PAC affects platform workers’ SVCB.

The Job Demand-Control Model [27] suggests that the effects of work contexts on individuals arise from the dynamic interaction between job demands and job control. In the gig economy, platform labor settings typically constitute a “high-demand–low-control” work context. In such settings, the place of work and workers’ behavioral space are compressed by algorithms [14,15]. As a result, platform workers commonly experience being controlled by algorithms. However, individuals do not respond to such controlling contexts in the same way. An important source of this variation lies in their pre-existing negative psychological orientation toward algorithmic technology. Previous research identified the phenomenon of “algorithm aversion” (AAV). Compared with human decision-makers, people lose trust in algorithms more quickly and more completely when algorithms make mistakes. Even when algorithms objectively outperform humans, users may still reject them [28]. Subsequent studies further suggested that resistance to algorithms may also stem from factors such as the opacity of algorithms and the deprivation of control [29]. On this basis, AAV has been defined as the negative emotions, unfavorable attitudes, and avoidance tendencies that users display toward the recommendations and services of artificial intelligence algorithms [30].

Across the existing literature, AAV is generally understood as a relatively stable negative psychological tendency toward algorithmic intervention. Building on this view and considering the specific “high-demand–low-control” context of the gig economy, the present study defines AAV as a relatively stable negative psychological tendency that platform workers hold toward algorithmic decision-making and intervention in the labor process. This tendency is manifested in emotional reactions such as uneasiness, aversion, and concern when workers face algorithmic control [28,31]. According to Trait Activation Theory, specific cues in work contexts can activate individuals’ relevant latent traits or tendencies, thereby systematically shaping their subsequent responses [32]. In the present study, the “high-demand–low-control” context of gig platforms constitutes a key situational cue that activates the stable tendency of AAV. For individuals with higher levels of AAV, such situational cues are more likely to activate and reinforce pre-existing negative cognitive and affective patterns. As a result, these individuals may be more sensitive to experiences of being controlled and objectified in the labor process. Therefore, the present study introduces AAV into the theoretical model and examines its role as a moderator in the relationship between PAC and WO.

**Fig 1:**
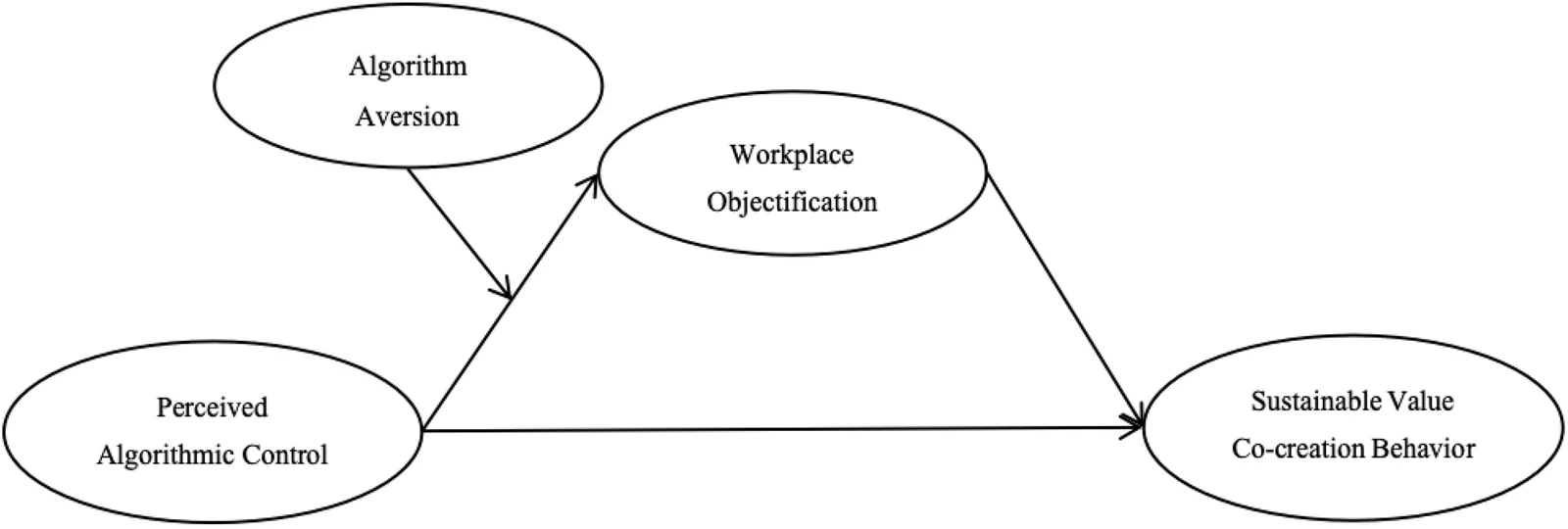
Theoretical model.

In summary, based on Objectification Theory and Trait Activation Theory, the present study develops a moderated mediation model to examine the effect of PAC on platform workers’ SVCB, as well as the mediating role of WO and the moderating role of AAV. The theoretical model is shown in Fig 1. The marginal contributions of this study are as follows: (1) by treating SVCB as an important behavioral outcome of PAC, this study extends research on the behavioral consequences of PAC among platform workers, thereby enriching empirical research on the behavioral mechanisms of individuals in the gig economy; (2) it introduces the perspective of WO to reveal how platform management practices trigger workers’ subjective experience of being “instrumentalized,” thereby explaining the underlying psychological mechanism through which PAC affects SVCB and further demonstrating the applicability of this concept in algorithmic management contexts; and (3) it introduces AAV as a moderating variable, showing that PAC functions as a technologized situational cue whose effect is shaped by individuals’ pre-existing attitudes toward algorithms, thereby further revealing the individual-difference mechanism through which PAC influences WO and providing a theoretical basis for more targeted interventions aimed at platform workers’ behavior.

## Theoretical foundations and research hypotheses

### Perceived algorithmic control (PAC) and sustainable value co-creation behavior (SVCB)

Unlike traditional organizational or leadership control, algorithmic control has become increasingly dominant on OLPs. These OLPs employ algorithmic systems to conduct virtual surveillance, continuously enhancing efficiency and performance, thereby establishing a full-cycle governance model [15]. Throughout this process, platform workers increasingly perceive themselves to be under tight algorithmic supervision, with limited autonomy and discretion, giving rise to the experience of PAC [33]. PAC refers to workers’ cognitive and emotional perceptions of algorithmic intervention, comprising three dimensions: normative guidance, tracking and evaluation, and behavioral constraint [33]. Normative guidance denotes the delivery of complex task requirements; tracking and evaluation involve stringent monitoring and performance assessment; and behavioral constraint refers to the immediate enforcement of rewards and penalties. On most OLPs, algorithmic systems govern the entire labor process—from task allocation to performance appraisal—undermining workers’ autonomy and discretion. However, SVCB relies heavily on intrinsic motivation and autonomy [34].Therefore, this study posits that PAC may undermine workers’ engagement in SVCB.

This inhibiting effect is primarily driven by the following factors: First, algorithmic systems are efficiency-oriented and optimize task allocation through standardized processes [10]. In a high PAC environment, workers tend to perceive their relationship with the platform as transactional, focusing on performance rather than relational investment. As a result, they may withhold emotional and cognitive resources, aiming only to fulfil minimum task requirements [35]. When workers further perceive that the platform’s algorithmic control is primarily cost-driven and lacks long-term developmental support, their emotional attachment to the platform erodes [36]. This weakens their motivation for proactive innovation and deep collaboration, ultimately suppressing their engagement in SVCB.

Second, the algorithmic normative and tracking mechanisms restrict autonomy and compress the space for platform workers’ innovation. The detailed task requirements delivered by algorithms (e.g., delivery time constraints) combined with stringent monitoring measures (such as real-time location tracking and process recording) create a “high demands–low control” work environment [33]. Prior research has established autonomy as a critical driver of proactive behavior [37]. Recent related research also shows that role stress and job autonomy jointly shape workers’ psychological recovery and physical and mental well-being, further indicating that autonomy is an important work resource in flexible employment contexts [38] When autonomy is stripped away by algorithmic management, platform workers are confined to executing tasks according to fixed procedures, losing the ability to adjust their work methods [39], which in turn diminishes their sense of control over their work. Since SVCB requires workers’ continuous active engagement [40], under conditions of constrained autonomy, platform workers find it difficult to propose improvements or innovations and tend to perform tasks mechanically rather than actively optimizing services.

Furthermore, OLPs reinforce behavioral constraints on workers through immediate reward and penalty mechanisms [33]. To avoid sanctions, workers tend to prioritize short-term, compliance-oriented cooperation [41]. However, SVCB depends on collaborative interaction between individuals and organizations [42,43]. The unidirectional obedience imposed by algorithmic control deprives platform workers of emotional support and feedback channels, leading them to perform tasks mechanically [44,45] and to no longer regard proactive value creation behaviors as part of their work responsibilities, thereby reducing ongoing value co-creation with the platform. Therefore, PAC undermines platform workers’ emotional investment, autonomy, and social support through task execution, normative guidance, and behavioral constraints, intensifying psychological strain and behavioral restrictions, and ultimately weakening their capacity and willingness to engage in SVCB. On this basis, the hypothesis below is developed:

H1: PAC negatively affects platform workers’ SVCB.

### The mediating role of workplace objectification (WO)

WO refers to the self-perception process through which individuals come to view themselves as objects in the workplace, whereby their autonomy and human essence are denied [25,46]. Based on objectification theory, highly standardized and controlled work environments reduce workers to replaceable production factors [46], leading to a dual experience of instrumentalization and vulnerabilities for platform workers. Within the setting of OLPs, platform workers often experience a dual process of instrumentalization and vulnerabilities. Instrumentalization occurs when algorithms reduce workers’ value to performance indicators—such as order volume or punctuality—while neglecting their professional development or emotional needs. Vulnerability, meanwhile, manifests in excessive task allocation driven by algorithmic efficiency, disregarding workers’ physical well-being [26]. Within algorithm-dominated labor systems, the phenomenon of WO is particularly prevalent.

This study argues that PAC positively predicts WO among platform workers. OLPs use algorithmic norms and performance-tracking systems to encourage compliance with efficiency targets [33]. Rules such as “delays result in penalty points” or “negative reviews affect task assignment” constrain worker behavior within algorithmic parameters [15]. Real-time tracking and ranking systems further reduce workers’ individuality to quantifiable data [47]. As workers internalize these logics, they are compelled to comply in order to maintain work opportunities, increasingly perceiving themselves as mechanical extensions of the system—thus heightening their experience of WO.

The algorithmic control mechanism systematically overlooks platform workers’ health rights, fostering a perception of physical vulnerability [48]. In pursuit of efficiency, OLPs often assign tasks that exceed normal workloads or schedule dense, time-consuming routes. As a result, platform workers may directly feel that they are controlled by algorithms and come to regard their bodies as tools for achieving platform interests. For instance, delivery riders frequently receive multiple cross-regional orders within tight timeframes. Despite physical fatigue, they must rush to meet system-imposed countdowns [49]. Prolonged exposure to such high-intensity conditions reinforces the belief that algorithmic rules ignore physical limits and that the platform disregards worker health. This deepened sense of control leads to the perception that health is subordinate to efficiency, heightening the experience of WO. Thus, PAC intensifies platform workers’ WO.

WO further impedes platform workers’ SVCB. SVCB entails proactive engagement and discretionary effort [50], yet objectified workers often withdraw effort and limit performance [51]. According to objectification theory, continual evaluation through an instrumental lens erodes workers’ sense of purpose, leading them to equate self-worth with measurable output [52]. Treated as manipulable tools, they lose autonomy and cannot meaningfully engage in service innovation or process optimization [53], often settling for minimum task fulfilment.

Moreover, emotional engagement is essential for sustained co-creation [54]. Workers reduced to “data points” by algorithmic systems experience reinforced manipulation and instrumentalization [55], hindering deep cognitive or emotional connection with their work. WO also leads to emotional alienation and burnout [51], diminishing willingness to contribute beyond basic duties. Thus, platform workers in objectifying environments are less inclined to engage in emotionally driven SVCB, further inhibiting proactive value co-creation.

In conclusion, PAC leads to WO via the instrumentalization and vulnerability of workers, thereby inhibiting SVCB. This research advances the subsequent hypotheses:

H2a: PAC positively influences platform workers’ WO.

H2b: WO negatively influences platform workers’ SVCB.

H2c: WO mediates the relationship between PAC and SVCB.

### The moderating role of algorithm aversion (AAV)

According to the Job Demand–Control Model, an individual’s psychological and behavioral reactions stem from the interaction of job demands and their perceived control [27]. Within OLPs, algorithms impose high work demands through real-time tracking and strict time assessments, while restricting autonomy via rigid task allocation systems and limited rejection options [33]. This results in platform workers increasingly perceiving themselves as tools of the system, leading to heightened PAC and, consequently, greater WO.

Yet, individuals do not uniformly respond to algorithmic control. As noted by Zou, Zhang [56], psychological traits influence how people perceive and react to identical environments. One such trait is AAV—a dispositional resistance to algorithmic decision-making, marked by cognitive distrust, emotional discomfort, and behavioral rejection of algorithmic authority [28,31]. Trait Activation Theory suggests that such latent traits are triggered by relevant contextual cues [32]. Research suggests that strong work events characterized by disruption and criticality can trigger employees’ psychological strain and further influence their subsequent behavioral responses [57]. Similarly, in the context of platform work, rigid and unpredictable algorithmic cues may also function as salient situational signals that intensify workers’ negative interpretations of algorithmic control. Specifically, AAV is likely to be activated by two key situational cues.

In the gig economy’s “high-demand, low-control” environments, AAV is likely to be activated by two key situational cues. The first involves rigid rule-based cues—such as non-negotiable review systems and mandatory task assignments—signaling a lack of human discretion and autonomy [58]. The second entails unpredictable cues, such as sudden pricing changes or opaque algorithmic decisions, which introduce uncertainty and amplify AAV’s defensive response. Together, these cues reinforce the belief that “algorithms equal control,” especially among workers predisposed to algorithmic distrust.

When such cues are encountered, individuals with high AAV are more likely to experience intensified WO, both cognitively and emotionally. Cognitively, they interpret algorithmic instructions as reflecting disregard for human needs [59]. For instance, unrelenting deadlines are viewed not just as work stressors but as symbolic denials of their physical limits. Emotionally, these cues elicit stronger perceptions of loss of control and negative affective responses [60]. In line with Trait Activation Theory, the closer the match between trait and environment, the more intense the trait expression [32]. Therefore, under the same level of PAC, workers with high AAV experience significantly stronger WO than those with low AAV.

Moreover, high AAV individuals often possess entrenched schemas linking algorithmic management with neglect of worker wellbeing. When OLPs push dense delivery schedules without regulating rest or accounting for fatigue [25], these individuals are especially likely to interpret such practices as objectifying. They see themselves as expendable, evaluated solely by metrics like task completion or customer ratings, and deprived of their rights to health, agency, and recognition [26]. This reinforces the internalization of their role as a disposable tool, further deepening their experience of WO.

Based on these observations, this study proposes the following hypothesis:

H3a: AAV positively moderates the relationship between PAC and platform workers’ WO.

In conclusion, the findings of this study provide robust evidence for the proposed moderated mediation model. We demonstrate that PAC adversely affects platform workers’ SVCB, with WO acting as a significant mediator in this association. Crucially, this mediated pathway is contingent upon a worker’s AAV. Specifically, for those with a higher AAV, the positive relationship between PAC and WO is notably stronger, thereby amplifying the negative indirect effect of PAC on SVCB via WO.

H3b: AAV positively moderates the indirect effect of PAC on platform workers’ SVCB through WO. In other words, the stronger the AAV, the stronger the negative impact of PAC on SVCB through WO.

## Study 1: Two-wave survey study

### Sample and procedure

This study received ethical approval from the Ethics Committee of Nanjing University of Technology (Approval No. NTJTECH-1–21). The recruitment period for this study is from November 1, 2024, through February 28, 2025. Data were collected via Credamo and Wenjuanxing, two widely used Chinese online platforms comparable to Prolific and MTurk. These platforms offer access to diverse participant pools and support flexible questionnaire design, thereby enhancing data quality. Before the survey began, all participants were asked to read an electronic informed consent form describing the purpose of the study, the procedures involved, and their right to withdraw at any time. Only those who agreed to participate were allowed to continue. All data were collected anonymously to ensure confidentiality. The sample in this study focused on workers from Chinese online labor platforms in the ride-hailing and delivery sectors. To ensure that the sample matched the target population, a screening question was included prior to the formal survey: “Do you work on an online labor platform in the ride-hailing or delivery sector (e.g., Didi, Meituan, Ele.me)?” Participants who answered “No” were automatically screened out.

This study adopted a two-wave time-lagged design, with data collected at two time points approximately one month apart, to reduce common method bias [61]. At Time 1, participants’ AAV and demographic variables were measured first, followed by PAC. When measuring AAV, the instructions explicitly asked participants to respond based on their “long-term and consistent overall feelings and attitudes toward algorithmic technology.” At Time 2, participants from the first wave were invited to complete measures of WO and SVCB. To ensure data quality and participant experience, (1) the questionnaires were distributed through the online platforms Credamo and Wenjuanxing, allowing participants to pause and resume the survey, but not to go back and revise previously answered items; and (2) during data cleaning, responses with unusually short completion times, obvious patterned responding, or failed attention checks (e.g., “Please select the third option for this item”) were excluded. Each participant who completed both waves received RMB 5 (approximately USD 0.71) as compensation.

In the first wave, 350 questionnaires were distributed, and 323 valid responses were obtained (response rate = 92.29%). A month later, in the second wave, questionnaires were distributed to these 323 participants. After excluding inattentive responses and questionnaires with mismatched identification codes, 285 valid questionnaires were retained (effective response rate = 88.24%).

Among the final sample, 177 participants (62.1%) were male and 108 (37.9%) were female. Most respondents (87.3%) were aged between 21 and 40. In terms of work experience, 98 participants (34.4%) had less than three years. Regarding education, 121 (42.4%) had a high school diploma or below, 114 (40.0%) held a college degree, and 50 (17.5%) held a bachelor’s degree. A total of 212 respondents (74.4%) were full-time platform workers, while 73 (25.6%) were part-time.

### Measures

In this study, all scales used are from established, authoritative journals and have been empirically validated. For the English scales, a “translation-back-translation” method was employed to convert them into Chinese, followed by multiple rounds of empirical testing. The study employs a 5-point Likert scale, asking participants to rate their level of agreement or disagreement with the items (1 = “Strongly Disagree” to 5 = “Strongly Agree”).

Perceived Algorithmic Control (PAC): PAC was measured using an 11-item scale [33]. A sample item is: “The platform system (algorithm) tracks and locates my geographical position in real time.” Cronbach’s α, which was used to assess the internal consistency reliability of the scale, was 0.903.

Workplace Objectification (WO): WO was measured using a 10-item adapted scale [26]. A sample item is: “The platform cares more about what I can do for them, rather than what they can do for me.” Cronbach’s α was 0.944.

Sustainable Value Co-Creation Behavior (SVCB): SVCB was measured using a 5-item adapted scale [62]. A sample item is: “In my future work or services, I will continue to provide feedback to the platform that contributes to service improvement.” Cronbach’s α was 0.878.

Algorithm Aversion (AAV): AAV was measured using a 3-item scale [30]. A sample item is: “The platform’s algorithm knows my preferences so well that it makes me uncomfortable.” Cronbach’s α was 0.796.

The item statements for all variables are provided in S1 Appendix.

Control Variables: Consistent with previous studies [63,64], this study includes gender, age, education level, job type, and work experience as control variables.

### Data analysis

Data were analyzed using SPSS 27.0 [65] and Mplus 8.3 [66]. First, confirmatory factor analysis was conducted to examine the discriminant validity among the core variables and to assess the adequacy of the measurement model. Second, common method bias tests, descriptive statistics, and correlation analyses were performed to provide a preliminary assessment of the relationships among the variables. Finally, hierarchical regression analyses and the PROCESS macro [65] were used to test the mediation effect, moderation effect, and moderated mediation effect, thereby providing further support for the theoretical model.

### Analysis results

#### Confirmatory factor analysis.

Confirmatory Factor Analysis (CFA) was conducted using Mplus 8.3 to assess the discriminant validity among the four key variables: PAC, SVCB, WO and AAV. To validate discriminant validity, a series of alternative models were constructed by combining the core variables into three-factor, two-factor, or one-factor structures for comparison. As presented in Table 1, the four-factor model exhibited robust fit indices (χ² = 831.134, CFI = 0.901, TLI = 0.889, RMSEA = 0.068, SRMR = 0.065). Crucially, this model significantly outperformed alternative models, thereby confirming strong discriminant validity across the variables and supporting their treatment as independent constructs in the subsequent hypothesis tests.

**Table 1:** Confirmatory factor analysis results.

MODEL	χ²	χ²/df	CFI	TLI	RMSEA	SRMR
Four-factor model: PAC; WO; AAV; SVCB	831.134	2.240^***^	0.901	0.889	0.068	0.065
Three-factor model A: PAC + AAV; WO; SVCB	1255.728	3.358^***^	0.824	0.809	0.091	0.075
Three-factor model B: PAC; WO + AAV; SVCB	1293.039	3.457^***^	0.807	0.790	0.093	0.092
Three-factor model C: PAC + WO; AAV; SVCB	1976.948	5.286^***^	0.680	0.652	0.123	0.135
Two-factor model A: PAC + AAV; WO+SVCB	1628.799	4.332^***^	0.737	0.716	0.108	0.094
Two-factor model B: PAC; WO + AAV+SVCB	1806.890	4.806^***^	0.714	0.691	0.116	0.105
One-factor model: PAC + WO + AAV+SVCB	2539.167	6.735^***^	0.546	0.511	0.142	0.138

Note: N = 285; PAC = Perceived Algorithmic Control, WO = Workplace Objectification, AAV = Algorithm Aversion, SVCB = Sustainable Value Co-creation Behavior, + indicates merging into a single factor, *** *p < 0.001.*

#### Common method bias.

To address common method bias, a Harman’s single-factor test was initially performed. This analysis revealed that the first unrotated factor explained 22.657% of the total variance, which is below the commonly accepted 40% threshold, suggesting that CMB is not a significant concern in this study [61], indicating minimal bias. Following the procedure proposed in [61], a latent common method factor was added to the hypothesized four-factor model to create a common latent factor (CLF) model. Comparison showed no significant improvement in model fit for the five-factor model, with negligible changes in fit indices (ΔCFI = 0.015, ΔTLI = 0.012, ΔRMSEA = 0.002). Consequently, common method bias is not considered a significant concern for the validity of this study’s findings.

#### Correlation analysis, measure reliability and validity.

The descriptive statistics for the main variables and control variables were analyzed using SPSS 27.0, with results shown in Table 2. All correlations were within a reasonable range, with the highest correlation at 0.580. The maximum variance inflation factor (VIF) was 1.271, indicating no severe multicollinearity. Average variance extracted (AVE) and composite reliability (CR) were also assessed. As shown in Table 2, AVE exceeded 0.5 and CR exceeded 0.7, meeting statistical standards [67], confirming good convergent and discriminant validity. Table 2 also shows a significant positive correlation between PAC and WO (r = 0.421, p < 0.001), and a significant negative correlation between WO and SVCB (r = − 0.499, p < 0.001), PAC was significantly and negatively correlated with SVCB (r = −0.344, p < 0.001). Exact p-values for the correlation coefficients reported in Table 2 are provided in S2 Appendix. These results are consistent with the study hypotheses and provide preliminary support for the subsequent hypothesis testing.

**Table 2:** Mean, standard deviation, and correlation coefficients of variables.

Variable	M	SD	1	2	3	4	5	6	7	8	9
1.Gender	1.380	0.531									
2.Age	2.610	0.702	−0.068								
3.Education Level	3.580	1.023	0.149*	−0.254***							
4.Work Experience	3.480	1.555	−0.065	0.580***	−0.307***						
5.Job Type	1.260	0.437	−0.077	0.467*	−0.280***	0.460***					
6.PAC	3.650	0.651	0.034	−0.247***	0.333***	−0.266***	−0.280***	**0.713**			
7.WO	3.320	0.911	−0.066	−0.255***	0.268***	−0.255***	−0.243***	0.421***	**0.817**		
8.AAV	2.990	0.912	−0.054	0.004	−0.018	−0.004	0.005	0.243***	0.133*	**0.843**	
9.SVCB	2.420	0.874	0.131	0.172**	−0.099	0.171**	0.146*	−0.344***	−0.499***	−0.193***	**0.820**
Composite Reliability (CR)								0.919	0.952	0.710	0.672
Average Variance Extracted (AVE)								0.508	0.667	0.880	0.911

Note: N = 285; * p < 0.05, ** p < 0.01, *** p < 0.001. The coding for certain variables is as follows: Gender: Male = 1, Female = 2; Age: Below 20 = 1, 21–30 years = 2, 31–40 years = 3, 41–50 years = 4, Above 50 = 5; Education Level: Primary school or below = 1, Middle school = 2, General high school/vocational school/technical school/vocational high school = 3, Associate degree = 4, Bachelor’s degree or above = 5; Work Experience: Less than 1 year = 1, 1 year (inclusive) to less than 3 years = 2, 3 years (inclusive) to less than 5 years = 3, 5 years (inclusive) to less than 7 years = 4, 7 years (inclusive) to less than 9 years = 5, 9 years or more = 6; Job Type: Full-time = 1, Part-time = 2. Diagonal elements represent the square root of AVE, and the bottom rows report CR and AVE values for each construct.

#### Hypothesis testing.

Main effect test. Hierarchical regression analysis was conducted using SPSS 27.0. First, only the control variables, such as gender and age, were entered in Model 4 of Table 3; the independent variable PAC was then added in Model 5. The results showed that, after controlling for demographic variables, PAC still had a significant negative effect on SVCB (β = −0.431, p < 0.001), and the explanatory power of the model increased significantly (ΔR² = 0.085). It is worth noting that although gender was significant in Model 5 (β = 0.265, p = 0.009), the effect of the core independent variable remained robust. Therefore, Hypothesis 1 was supported.

**Table 3:** Hierarchical regression analysis.

Variables	WO			SVCB		
	M1	M2	M3	M4	M5	M6
	β	β	β	β	β	β
**Control Variables**						
Gender	−0.117*	−0.205*	−0.198*	0.155**	0.265**	0.182
Age	−0.113	−0.116	−0.116	0.092	0.087	0.040
Education Level	0.201***	0.104*	0.103*	−0.056	0.021	0.063
Work Experience	−0.088	−0.035	−0.039	0.082	0.030	0.016
Job Type	−0.102	−0.117	−0.147	0.061	0.034	−0.014
**Independent Variable**						
PAC		0.466***	0.553***		−0.431***	−0.243**
**Mediating Variable**						
WO						−0.405***
**Moderating Variable**						
AAV			0.004			
**Interaction Term**						
PAC × AAV			0.279**			
R^2^	0.138	0.230	0.265	0.065	0.141	0.283
ΔR^2^	0.138	0.093	0.033	0.065	0.085	0.142
F	8.910***	13.860***	12.468***	3.850**	7.619***	15.615***

Note: * p < 0.05, ** p < 0.01, *** p < 0.001.

**Mediation effect test.** To test this hypothesis, the present study used the PROCESS 4.2 macro with 5,000 bootstrap resamples to estimate the confidence interval of the mediation effect. As shown in Table 3, after controlling for demographic variables, Model 2 indicated that PAC had a significant positive effect on WO (β = 0.466, p < 0.001). Model 6 showed that WO had a significant negative effect on SVCB (β = −0.405, p < 0.001). Exact p-values for the coefficients reported in Table 3 are provided in S3 Appendix. These results suggest that, after controlling for demographic variables, WO continued to play a stable mediating role in the relationship between PAC and SVCB. In addition, according to the bootstrap results reported in Table 4, the indirect effect of PAC on SVCB through WO was −0.189, with a 95% confidence interval of [−0.283, −0.113], excluding zero, indicating a significant mediation effect. Therefore, Hypotheses 2a, 2b, and 2c were supported.

**Table 4:** Mediating effect test.

Path: PAC – WO – SVCB			
Effect Type	Effect Value	SE	95% CI
**Total E ffect** PAC – SVCB	−0.431	0.081	[-0.591, -0.271]
**Direct Effect** PAC – SVCB	−0.243	0.079	[-0.398, -0.088]
**Indirect Effect** PAC – WO – SVCB	−0.189	0.044	[-0.283, -0.113]

Moderating effect test. Before testing the moderating effect of AAV, PAC and AAV were mean-centered by subtracting the sample mean of each variable from its observed values, following prior recommendations [68]. An interaction term between PAC and AAV was then created and entered in the regression model. This procedure not only effectively reduced the potential multicollinearity between the interaction term and the main effects but also improved the interpretability of the regression coefficients. The moderating effect was then tested using the PROCESS 4.2 macro. As shown in Model 3 of Table 3, the interaction term between PAC and AAV had a significant positive effect on WO (β = 0.279, p = 0.001), indicating that AAV significantly moderated the relationship between PAC and WO.

**Fig 2:**
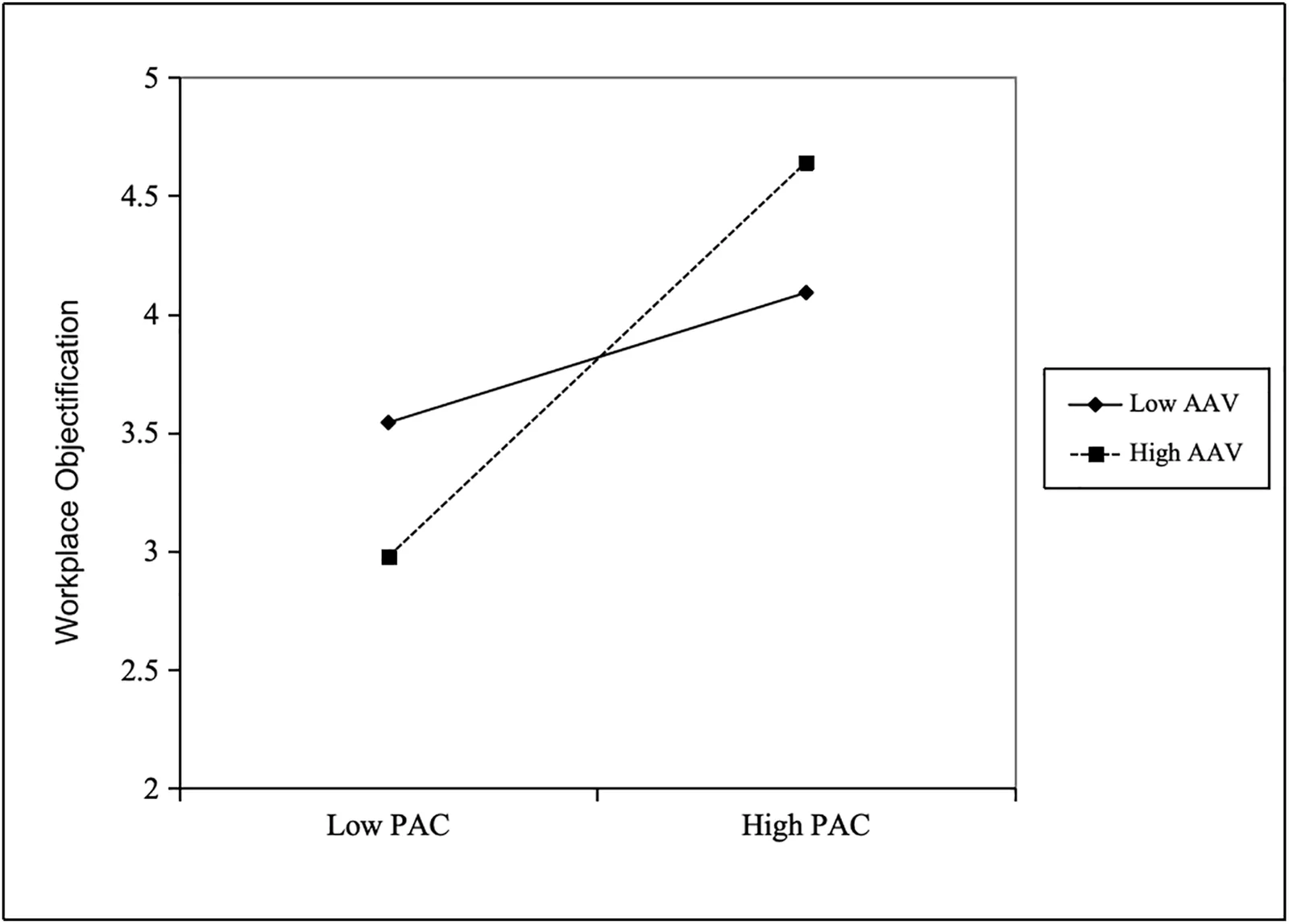
Moderation effect of AAV.

Following prior research [69], a simple slope analysis was further conducted by plotting the interaction effect at high and low levels of AAV. As shown in Fig 2, when AAV was high (M + 1 SD), the positive effect of PAC on WO became stronger (simple slope = 0.832, t = 5.371, p < 0.001); when AAV was low (M-1 SD), the effect of PAC on WO was not significant (simple slope = 0.274, t = 0.903, p = 0.367). These results indicate that the higher the level of AAV, the more likely platform workers were to experience WO under strong algorithmic control. Therefore, Hypothesis 3a was supported.

Moderated mediation effect test. This study assessed whether the indirect effect of PAC on SVCB via WO is moderated by AAV, using PROCESS 4.2 macro. The moderated mediation index was −0.113, with a 95% confidence interval of [−0.184, −0.046], excluding zero, confirming the moderated mediation. As shown in Table 5, when AAV is high, the indirect effect of PAC on SVCB through WO is strongest (−0.327, SE = 0.069, 95% CI [−0.473, −0.201]), with the interval excluding zero. When AAV is low, the indirect effect remains significant but weaker (−0.121, SE = 0.041, 95% CI [−0.213, −0.049]). The difference in mediation effects between high and low AAV is significant (−0.224, SE = 0.047, 95% CI [−0.324, −0.139]), confirming that AAV positively moderates the indirect effect of PAC on SVCB via WO. Hypothesis 3b is therefore supported.

**Table 5:** Results of moderated mediation effect test.

Mediator	Moderator	Effect Size	SE	95% CI
WO	Low AAV (M-1SD)	−0.121	0.041	[-0.213, -0.049]
	High AAV (M + 1SD)	−0.327	0.069	[-0.473, -0.201]
	Difference	−0.224	0.047	[-0.324, -0.139]

#### Discussion.

Study 1 provided preliminary support for the negative effect of PAC on SVCB, the mediating role of WO, and the moderating role of AAV through a two-wave time-lagged survey design. Specifically, PAC significantly and positively predicted WO (M2: β = 0.466, p < 0.001; M3: β = 0.553, p < 0.001) and significantly and negatively predicted SVCB (M5: β = −0.431, p < 0.001; M6: β = −0.243, p < 0.01). At the same time, WO had a significant negative effect on SVCB (M6: β = −0.405, p < 0.001). In addition, the interaction term between PAC and AAV had a significant positive effect on WO (M3: β = 0.279, p < 0.01), indicating that AAV strengthened the effect of PAC on WO. Overall, these findings supported the theoretical hypotheses of the present study.

In the model-building process, this study first established baseline models containing only control variables (M1 and M4), and then gradually introduced the core variables in order to test the independent effect of PAC more clearly. The results showed that some demographic variables were significant in several models. Specifically, in the WO models, gender had a significant negative effect in M1 and M2 (M1: β = −0.117, p < 0.05; M2: β = −0.205, p < 0.05), while education had a significant positive effect in M1, M2, and M3 (M1: β = 0.201, p < 0.001; M2: β = 0.104, p < 0.05; M3: β = 0.103, p < 0.05). In the SVCB models, gender had a significant positive effect in M4 and M5 (M4: β = 0.155, p < 0.01; M5: β = 0.265, p < 0.01), whereas age, work experience, and job type did not show stable significant effects in this study. These results suggest that platform workers with different demographic characteristics may differ in their perceptions and behavioral responses under algorithmic control.

More importantly, after controlling for the above demographic variables, the effects of PAC on WO and SVCB remained significant, and the mediating effect of WO also remained significant. This suggests that the proposed pathway of “PAC–WO–SVCB” was relatively robust in the current sample. However, because Study 1 was mainly based on survey data, the test of the full pathway still relied on correlational evidence. Therefore, Study 2 further adopted a scenario-based experimental method to provide more direct causal evidence for the effect of PAC on WO and the moderating role of AAV, thereby strengthening the internal validity of the findings.

## Study 2: Scenario-based experiment

Although Study 1 collected data at multiple time points, all variables were self-reported, with PAC and AAV measured simultaneously, potentially limiting causal identification of the relationship between the two variables to some extent. Therefore, Study 2 adopted a scenario-based experiment to provide more direct causal evidence for the effect of PAC on WO and the moderating role of AAV, thereby strengthening the internal validity of the findings. Specifically, it tests the effect of PAC on WO (H2a) and the moderating role of AAV in this mechanism (H3a).

### Design and participants

This experiment employed a between-subjects design with a single factor (PAC experimental group vs. control group). A total of 238 platform workers were recruited in China, informed that the survey was for academic purposes only, fully anonymous, and did not disclose personal information. The sample in this study focused on workers from Chinese online labor platforms in the ride-hailing and delivery sectors. To enhance sample quality and control for invalid responses, attention-check items (e.g., “Please select 3 for this question”) were included, yielding 216 valid samples. Using G*Power [70], the required sample size was calculated. With a medium effect size (d = 0.5), α = 0.05, and 1 − β = 0.80, at least 64 participants per group were needed. Therefore, the effective sample size met the standard. Among the valid participants, 38.5% were aged 21–30, 36.9% had 1 year (inclusive) to less than 3 years of work experience, and 63.5% were male.

### PAC manipulation and pretest

Drawing on prior scenario-based experimental materials [71], this study developed two sets of experimental texts: one to present a high-PAC experimental scenario and the other to serve as the control scenario. To ensure the validity of the manipulation materials, the author team conducted multiple rounds of internal discussion after completing the initial draft and invited two PhD students in the field of organizational behavior (OB) to evaluate and revise the materials. The revision process focused on the following aspects. First, the experimental-group materials were required to clearly present features of algorithmic control, such as algorithmic monitoring, automated dispatching, and performance evaluation, to create a sufficiently strong manipulation. Second, the control-group materials were required to remain consistent with the experimental-group materials in terms of cultural context and reading format, while excluding information related to algorithmic control, digital monitoring, platform governance, workplace supervision, or technological substitution. Third, the two sets of materials were made as similar as possible in length, reading difficulty, and narrative tone, to reduce the interference of irrelevant factors with the experimental results. The original experimental stimuli used in Study 2 are provided in S4 Appendix. The final experimental materials were then established.

Before the formal experiment, a pretest was conducted to examine the effectiveness of the PAC manipulation. A total of 136 platform workers were recruited for the pretest and were randomly assigned to either the high-PAC experimental group or the control group. Participants in the experimental group read an article emphasizing features of algorithmic control, whereas participants in the control group read an article that did not involve algorithmic control. PAC was then assessed using the same 11-item measure as in Study 1.

The independent-samples t-test showed that the PAC score of the high-PAC experimental group (M = 3.670, SD = 0.570) was significantly higher than that of the control group (M = 2.980, SD = 0.580), t(134) = 6.940, p < 0.001, 95% CI [0.490, 0.880], d = 1.190, indicating that the PAC manipulation was successful.

### Experiment procedure and measurement

To prevent participants from skipping instructions, the “Next” button remained inactive for 20 seconds. Before the formal scenario manipulation began, and following prior research [71], this study first measured the moderator AAV to obtain participants’ baseline levels prior to the scenario manipulation and to minimize, as much as possible, the influence of the subsequent experimental manipulation on the measurement of this variable.

Participants were then randomly assigned to two groups and asked to read different experimental materials. The materials were derived from the pretest and had passed the manipulation check. After reading the materials, participants first completed the PAC scale for the manipulation check and then completed the WO measure, using the same items as in Study 1. All items were measured on a 7-point Likert scale (1 = “strongly disagree,” 7 = “strongly agree”). Finally, participants provided demographic information. In terms of control variables, Study 2 retained the basic demographic variables used in Study 1, including gender, age, education, and work experience, and further incorporated daily working hours as a work-investment indicator considering the experimental context, to better reflect differences in platform labor intensity. Each participant received RMB 5 (approximately USD 0.71) after completing the experiment.

### Manipulation test

Independent samples t-test results from the main experiment indicated that the PAC score in the experimental group (M = 3.740, SD = 0.350) was significantly higher than that in the control group (M = 3.210, SD = 0.387), t(214) = 10.530, p < 0.001, 95% CI [0.431, 0.630], d = 0.815. This confirms the successful manipulation of PAC.

In addition, to examine the baseline equivalence of pre-manipulation AAV between the experimental and control groups, this study further compared the AAV levels of the two groups of participants. The independent-samples t-test showed a significant difference in AAV between the experimental and control groups (t = −6.732, p < 0.001). It should be emphasized that, in this experimental design, AAV was measured before the scenario manipulation materials were presented. Therefore, this statistical difference reflects baseline sample distribution characteristics arising during the random assignment process, rather than a state change induced by the experimental manipulation. To address this issue, AAV was subsequently entered into the regression equation as a pre-measured moderator in the follow-up regression analyses (see Table 7), thereby allowing for strict statistical control.

### Results

#### Reliability and validity analysis.

To ensure reliability, Cronbach’s α, average variance extracted (AVE), and composite reliability (CR) for WO and AAV were assessed. As shown in Table 6, all factor loadings exceeded 0.50, ranging from 0.629 to 0.874. In addition, all AVE values were above 0.50 and all CR values were above 0.70, meeting the relevant criteria [67] and indicating good convergent and discriminant validity.

**Table 6:** Results of reliability and validity analysis.

Constructs	Factor loadings	AVE	CR	Cronbach α
**Workplace Objectification**		**0.522**	**0.919**	**0.830**
WO1	0.699			
WO2	0.638			
WO3	0.743			
WO4	0.789			
WO5	0.710			
WO6	0.638			
WO7	0.682			
WO8	0.682			
WO9	0.629			
WO10	0.667			
**Algorithm Aversion**		**0.721**	**0.885**	**0.817**
AAV1	0.851			
AAV2	0.821			
AAV3	0.874			

#### Hypothesis testing.

Following prior research [72], this study included gender, age, education level, and work-related variables as control variables. In this analysis, AAV was treated as the moderator, PAC as the independent variable (coded as a dummy variable: PAC experimental group = 1, control group = 0), and WO as the dependent variable. A multilevel linear regression analysis was then conducted.

**Fig 3:**
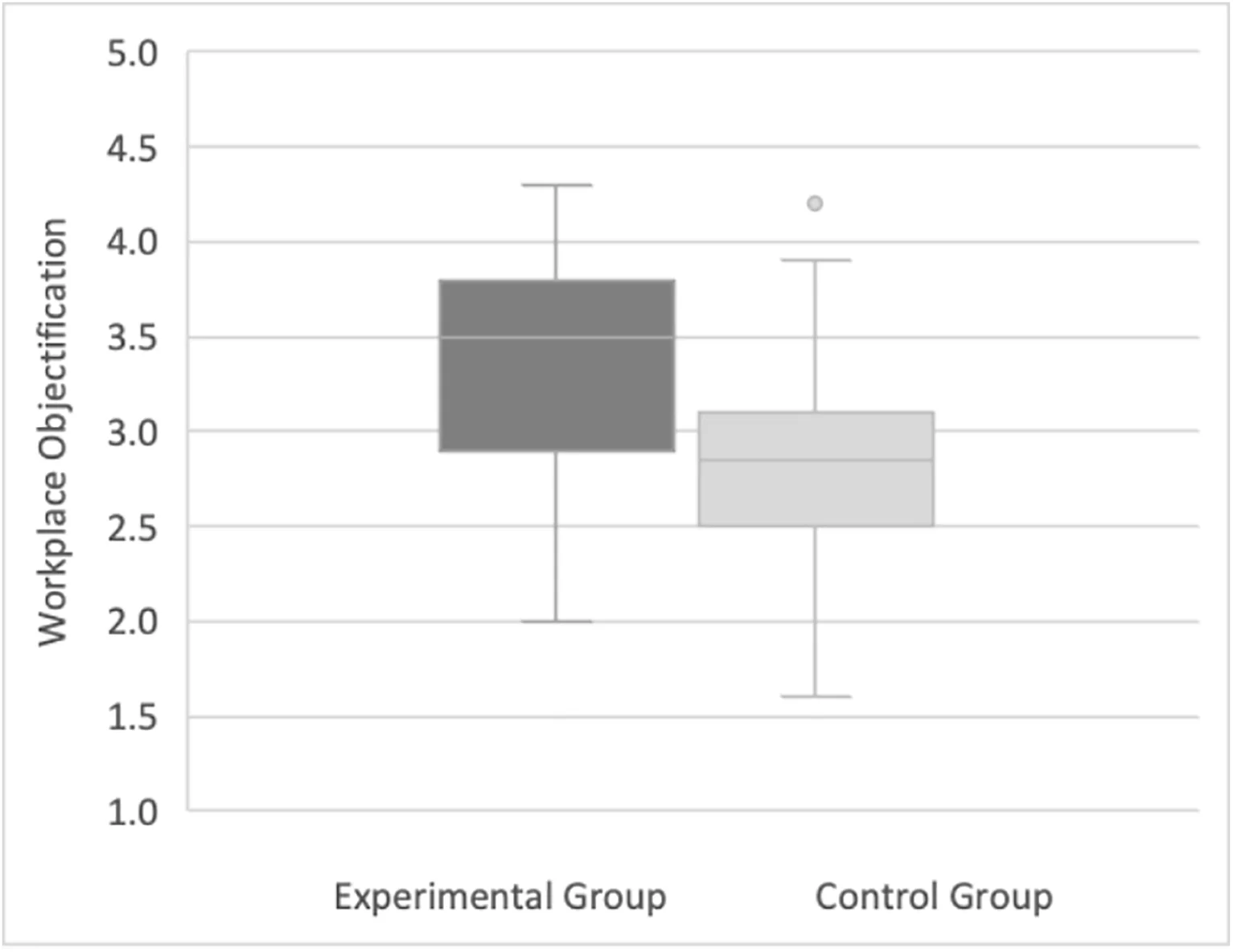
Boxplot of workplace objectification scores in the experimental and control groups.

Test of H2a. Fig 3 presents the distribution of WO scores in the PAC experimental group and the control group. The independent-samples t-test indicated that WO was significantly higher in the PAC experimental group (M = 3.390, SD = 0.539) than in the control group (M = 2.820, SD = 0.508), t(214) = −8.048, p < 0.001, 95% CI [−0.714, −0.433], d = −1.097, suggesting that stronger PAC was associated with higher levels of WO and thus provided preliminary support for Hypothesis 2a. A regression analysis was further conducted using the PROCESS 4.2 macro. As shown in Model 2 of Table 7, after controlling for demographic variables and entering only PAC as the independent variable, PAC still had a significant positive effect on WO (β = 0.502, p < 0.001). Exact p-values for the coefficients reported in Table 7 are provided in S5 Appendix. Therefore, Study 2 provided further support for the relationship between PAC and WO (H2a).

**Table 7:** Hierarchical regression analysis.

Variables	WO		
	M1	M2	M3
	β	β	β
**Control Variables**			
Gender	−0.139*	−0.069	−0.048
Age	0.253***	−0.070	0.061
Education level	0.128	0.019	0.024
Work experience	0.195**	0.054	0.044
Daily working hours	−0.150	0.017	−0.001
**Independent Variable**			
PAC		0.502***	0.435***
**Moderating Variable**			
AAV			0.089*
**Interaction Term**			
PAC × AAV			0.195*
R^2^	0.116	0.263	0.297
ΔR^2^	0.116	0.147	0.181
F	5.529***	12.401***	10.936***

Note: * p < 0.05, ** p < 0.01, *** p < 0.001. Daily working hours: Less than 2 hours = 1, 2(inclusive) to less than 4 hours = 2, 4 (inclusive) to less than 8 hours = 3, 8 (inclusive) hours or more = 4.

**Fig 4:**
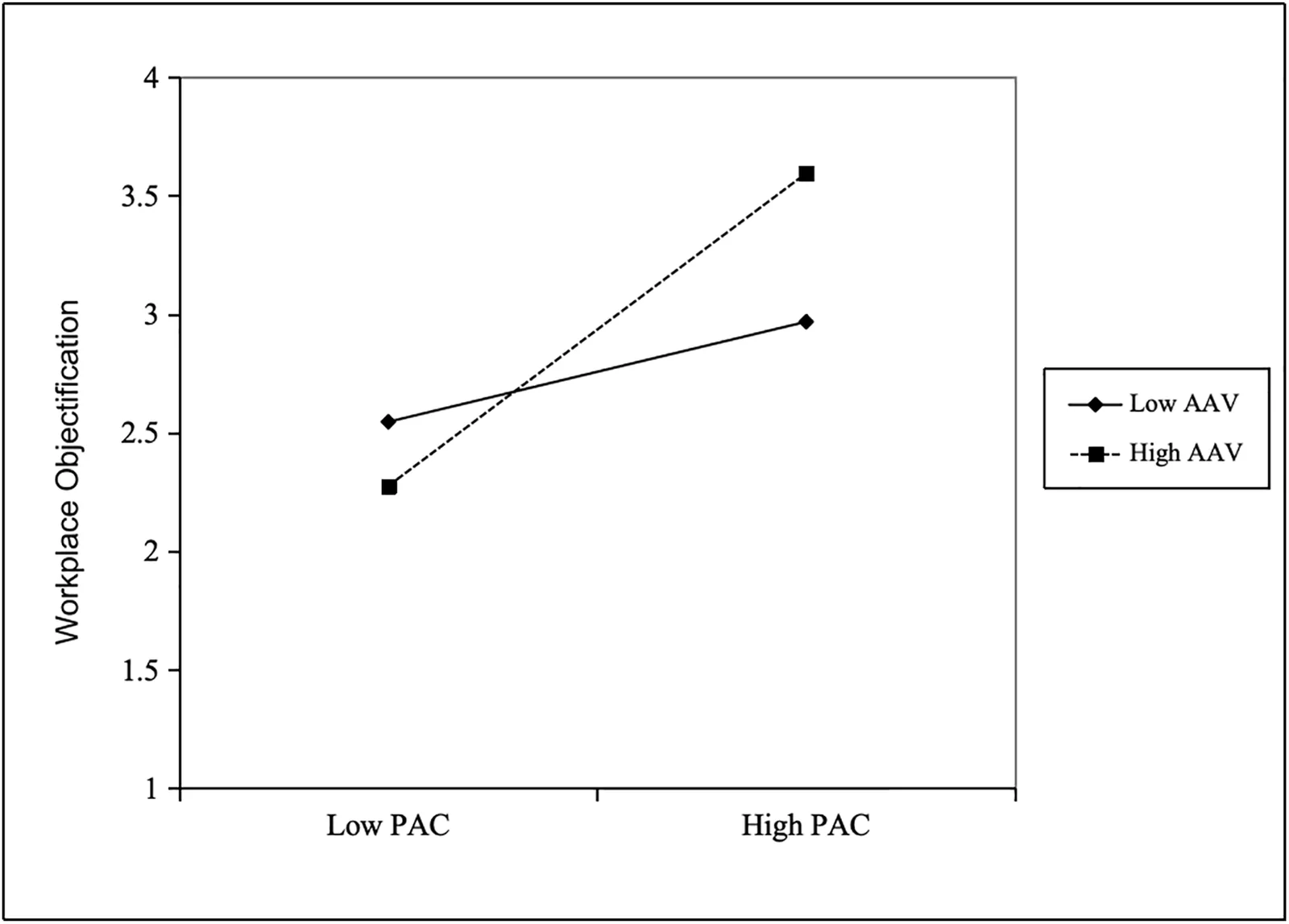
Moderation effect of AAV.

Test of H3a. The moderating effect of AAV on the relationship between PAC and WO was tested using the PROCESS 4.2 macro. As shown in Model 3 of Table 7, after including the main effect of AAV and the relevant control variables, the interaction term between PAC and AAV had a significant positive predictive effect on WO (β = 0.195, p < 0.05), providing preliminary evidence that AAV moderated the path through which PAC affected WO. A simple slope analysis was further conducted following prior research [69], and the interaction effect was plotted. As shown in Fig 4, when AAV was high (M + 1 SD), the positive effect of PAC on WO was stronger (simple slope = 0.659, t = 3.574, p < 0.001); when AAV was low (M – 1 SD), this effect was not significant (simple slope = 0.211, t = 0.594, p = 0.553). These results are consistent with the hypothesis, indicating that as individuals’ level of AAV increases, the positive effect of PAC on WO tends to become stronger. Therefore, Hypothesis 3a received experimental support.

### Discussion

Using a scenario-based experimental method, Study 2 provided more direct causal evidence for the relationship between PAC and WO and offered additional support for the moderating role of AAV. The results showed that, after controlling for demographic variables, PAC still had a significant positive effect on WO (M2: β = 0.502, p < 0.001; M3: β = 0.435, p < 0.001), indicating that stronger algorithmic control significantly increased platform workers’ experience of being instrumentalized. At the same time, AAV had a significant positive effect on WO (M3: β = 0.089, p < 0.05), and the interaction term between PAC and AAV was significant (M3: β = 0.195, p < 0.05), indicating that AAV strengthened the effect of PAC on WO. Therefore, Hypotheses 2a and 3a were supported again.

Study 2 also first established a baseline model containing only the control variables (M1). The results showed that gender, age, and work experience were significant predictors of WO in the baseline model, but their effects were no longer significant after PAC was entered into the model. This suggests that, compared with demographic differences, PAC had stronger explanatory power in accounting for WO among platform workers.

Overall, by using an experimental method, Study 2 addressed, to some extent, the limitations of Study 1 in identifying the relationship between PAC and WO. Together with the findings of Study 1, these results provided support for the theoretical model proposed in the present study.

## Discussion and implications

### General discussion

Taken together, the results of the two studies provide largely consistent support for the proposed theoretical model, namely, that PAC weakens platform workers’ SVCB, that WO serves as an important psychological mechanism in this process, and that AAV intensifies this effect. Study 1, based on a two-wave time-lagged survey, provided relatively comprehensive empirical evidence for the relationships among PAC, WO, SVCB, and AAV. The results showed that PAC was significantly negatively associated with SVCB, that WO mediated this relationship, and that AAV further strengthened the effect of PAC on WO as well as its indirect effect. Study 2 used a scenario-based experimental method and provided more direct causal evidence for the relationship between PAC and WO, while again supporting the moderating role of AAV.

It should be noted, however, that the full mediation model (PAC → WO → SVCB) and the moderated mediation model (H3b) were tested using correlational data from Study 1. Although Study 2 provided experimental evidence for the effect of PAC on WO and the moderating role of AAV, the complete mediating pathway and the moderated mediation effect remain correlational in nature and should not be interpreted as establishing strict causal ordering.

Taken together, the two studies suggest that the negative response of platform workers to PAC is mainly reflected in the strengthening of their experience of being instrumentalized, and that this psychological change further inhibits their SVCB.

### Theoretical implications

First, this study extends research on the behavioral consequences of PAC by focusing on SVCB. Previous studies on algorithmic control have mainly examined how online labor platforms affect workers’ economic interests, labor rights, and control over the work process, and early studies in this area relied largely on qualitative methods, such as research on algorithmic control and workers’ rights in platform labor [41]. Although empirical research has increased in recent years, most studies have focused on outcomes such as service performance, proactive behavior, work engagement, work stress, algorithmic fairness, constrained autonomy, and impaired well-being [73]. Less attention has been paid to how PAC affects platform workers’ SVCB. Unlike general service performance or short-term task completion, SVCB emphasizes workers’ continuous and proactive engagement in service interaction, relationship maintenance, problem solving, and value creation. By examining SVCB as an important behavioral outcome of PAC, this study shows that the effects of PAC are not limited to workers’ immediate performance, stress responses, or work attitudes, but may also extend to their sustained, cooperative, and relational behaviors. Thus, this study broadens the behavioral scope of research on the consequences of PAC and provides a new perspective for understanding how platform algorithmic management affects workers’ long-term value creation behavior.

Second, this study reveals the mediating role of workplace objectification, thereby enriching the theoretical pathways through which the negative effects of algorithmic management unfold. Existing research on objectification has predominantly focused on sexual objectification, particularly its adverse consequences for women, such as disordered eating, depressive symptoms, and diminished self-esteem [52,74]. Prior studies further suggest that algorithmic control not only regulates platform workers through external managerial mechanisms [75], but also shapes their self-understanding and emotional experiences through the internalization of objectifying psychological processes [25,46]. However, workplace objectification as a broader psychological phenomenon—especially how it emerges and intensifies within platform workers’ subjective experiences in digitalized and algorithm-driven work environments—has received limited scholarly attention. By introducing workplace objectification into the domain of algorithmic management, this study systematically examines its mediating role in the relationship between perceived algorithmic control and platform workers’ sustainable value co-creation behavior from the perspective of objectification theory. The findings contribute to a deeper understanding of the profound implications of algorithmic control for platform workers’ psychological well-being and work behavior, while also responding to ongoing academic debates over whether algorithms lead to the instrumentalization of labor. In doing so, this study advances the scholarly dialogue on algorithmic management and labor protection from normative discussion toward empirical analysis.

Third, this study further clarifies the theoretical boundary of WO in the context of platform algorithmic management. When explaining platform workers’ negative psychological experiences under algorithmic management, WO and organizational dehumanization are two related but distinct concepts. Organizational dehumanization emphasizes the denial of employees’ human attributes, such as emotion, warmth, individuality, and agency, and treats employees as objects lacking human qualities [23,24]. In contrast, WO focuses more on instrumental use in labor relations, that is, whether employees feel that they are treated as usable, controllable, and replaceable functional means [75]. Based on the research question of this study, PAC is mainly reflected in the ways platforms organize the labor process through task allocation, process monitoring, performance assessment, rating and ranking, and reward and punishment feedback [1]. These management practices are closely related to workers’ perceptions of their own instrumentality, replaceability, and functional positioning. Therefore, this study adopts WO as the theoretical lens for explaining how PAC affects SVCB. This does not mean that this study empirically proves that WO is superior to organizational dehumanization. Rather, it allows this study to more accurately address how algorithmic control may lead platform workers to experience being treated as instruments. Thus, this study further clarifies the conceptual boundary and applicability of WO in explaining the negative effects of platform algorithmic management, and provides a theoretical basis for future research to compare the differentiated roles of WO and organizational dehumanization in algorithmic management contexts.

Fourth, this study reveals the moderating role of AAV, showing that individuals’ pre-existing attitudes toward algorithms influence platform workers’ psychological responses to PAC. Existing research on AAV has mainly focused on issues such as technology acceptance, algorithmic fairness, and privacy concerns [76,77]. However, limited attention has been paid to how individuals’ pre-existing attitudes toward algorithms influence their psychological and behavioral responses in specific work contexts. Drawing on the Job Demand-Control Model, this study argues that platform labor settings are characterized by both high job demands and low autonomous control. Platform workers need to respond continuously to task assignments, service evaluations, and performance requirements, but they have relatively limited control over dispatching rules, evaluation standards, and reward and punishment mechanisms. In this context, PAC not only reflects the platform’s technologized management of the labor process, but also serves as an important situational cue through which platform workers understand their work conditions. Further drawing on Trait Activation Theory, this study argues that AAV, as individuals’ relatively stable pre-existing negative attitude toward algorithmic systems, shapes how workers interpret PAC as a situational cue. For platform workers with higher levels of AAV, stronger PAC is more likely to be interpreted as the platform system’s domination, compression, and instrumentalization of their labor process, and is more likely to be internalized as the self-understanding that they are merely instrumental resources within the platform system. This instrumentalized self-understanding may lead to stronger experiences of WO and further weaken SVCB. Based on this logic, this study uses Trait Activation Theory to explain the differentiated formation of platform workers’ psychological responses in the context of platform algorithmic management. Technologized and depersonalized algorithmic control, as an important situational cue, can activate individuals’ pre-existing attitudes toward algorithms and influence whether external algorithmic control is transformed into an instrumentalized understanding of the self. In this process, PAC represents the technologized situational cue in platform algorithmic management, WO reflects workers’ instrumentalized self-understanding under this cue, and AAV explains why this self-oriented psychological response differs across workers. Accordingly, this study enriches research on AAV and provides a more explanatory theoretical perspective for understanding how platform workers respond differentially to PAC.

### Managerial implications

First, platform firms should fully recognize the potential negative effects of algorithmic control on platform workers’ behavior and strive to maintain a balance between efficiency orientation and humane management. Although algorithmic control helps improve task-matching efficiency and reduce management costs, when platform workers are exposed to a high-intensity and low-autonomy control environment for a prolonged period, their sense of responsibility and willingness to cooperate may be weakened, which in turn may reduce SVCB. Therefore, when implementing algorithmic management, platforms should mitigate the negative consequences of excessive control by increasing rule transparency and preserving limited autonomy. Platforms should clearly explain to workers the basic criteria underlying task assignment and ranking, such as order distance, response speed, historical service quality, cancellation rate, and online working hours, as well as the approximate range of their relative weights. When orders are reassigned, accounts are restricted, or incentive eligibility changes, platforms should also provide understandable explanations rather than merely presenting generalized outcomes. At the same time, platforms can retain a certain degree of flexibility in areas such as shift selection, work pacing, and short-break arrangements, so that workers have limited but meaningful room for decision-making within established rules, thereby enhancing their sense of participation.

Second, platforms should pay close attention to WO and reduce platform workers’ sense of being treated as mere “tools of production.” Research suggests that once platform workers feel over time that they are only tools for task completion, their sense of belonging, proactivity, and willingness to engage in sustained cooperation may all decline. Therefore, platforms should reflect workers’ subject status in their institutional design. For example, platforms may strengthen interaction and connection among workers by establishing worker communities, experience-sharing spaces, local work groups, or online discussion forums. They may also adopt mechanisms such as showcasing excellent suggestions, adopting service improvement proposals, and sharing workers’ stories, so that workers feel that their experiences and opinions are noticed and valued by the platform. In addition, platforms may establish regular care mechanisms, such as sending fatigue reminders after periods of heavy workload, providing channels for human communication when appeals become frequent or ratings fluctuate abnormally, and offering basic psychological support resources to workers in need, so as to alleviate the negative effects associated with the experience of being instrumentalized.

Third, platforms should pay attention to and proactively address platform workers’ AAV, particularly by helping them reduce concerns about the opacity, unfairness, and uncontrollability of algorithms. For workers with higher levels of AAV, merely requiring them to adapt to algorithmic management is unlikely to be effective; a more effective approach is to enhance explanation, communication, and participation. On the one hand, platforms can help workers understand the basic operating logic of algorithms in task assignment, evaluation, incentives, and risk control through rule explanation pages, short video training, online Q&A, and case demonstrations, thereby reducing resistance arising from unfamiliarity and uncertainty. On the other hand, platforms should establish an operable two-way feedback mechanism, so that workers are not only recipients of algorithmic rules but also participants in the optimization process. For example, platforms can set up in-app channels for “task assignment appeals,” “rule feedback,” and “error correction for abnormal identification,” allowing workers to submit feedback on clearly unreasonable order assignments, route planning, evaluation results, or penalty decisions. At the same time, platforms should set fixed feedback processing cycles, such as automatic responses within 48 hours and final handling results within 72 hours, and regularly disclose frequent issues and explanations of optimization measures. For recurring problems, platforms may also invite worker representatives to participate in testing sessions, rule discussion meetings, or feedback collection before version updates, so that feedback genuinely enters the closed loop of algorithm optimization rather than remaining at the level of symbolic consultation.

Fourth, platform firms should also enhance workers’ adaptability and sense of development in algorithmic management environments through training and career support. Because the job demands and technological environment of platform labor change rapidly, relying solely on algorithmic dispatch is insufficient to build stable cooperative relationships. Platforms can provide practical training on topics such as order-taking strategies, service communication, exception handling, and the use of digital tools, thereby helping workers improve their capabilities in the platform environment. At the same time, platforms may also offer career development support, such as tiered training, skill certification, service-level promotion pathways, or opportunities for cross-position transition, so that workers can see themselves not merely as labor used to complete immediate tasks, but as cooperative actors capable of continuous growth. In addition, platform managers and operations staff should also receive corresponding training to avoid evaluating workers solely on the basis of efficiency indicators, and instead demonstrate more respect, explanation, and support in daily management, thereby improving the sustainability of platform governance.

Finally, from an ethical and policy perspective, this study suggests that platform algorithm governance should not remain at the level of firms’ self-optimization alone, but also requires institutional constraints from external regulatory frameworks. If platforms rely for a prolonged period on opaque and one-dimensional algorithmic control, this may not only intensify platform workers’ experience of WO, but also undermine basic fairness and dignity in labor relations. In recent years, the EU AI Act and the Platform Work Directive have begun to impose clearer requirements on algorithmic management in terms of risk management, transparency, human oversight, and the regulation of algorithmic systems used in platform work. These institutional arrangements help alleviate excessive control and information asymmetry in platform labor. At the same time, relevant Chinese policies on protecting the rights and interests of workers in new forms of employment also emphasize platform rule transparency, the standardization of labor management, and the protection of workers’ rights. Taken together, these developments suggest that future platform governance should focus not only on efficiency and matching, but also on establishing more comprehensive institutional arrangements regarding rule transparency, appeals and remedies, human intervention, and worker participation, so as to reduce the negative consequences brought about by PAC and WO.

### Limitations and future directions

Despite achieving certain results, this study has the following limitations. First, the sample in this study was drawn mainly from multiple gig platforms in China, and the platform types were concentrated primarily in labor settings such as ride-hailing and food delivery, which rely heavily on algorithmic dispatching and real-time monitoring. Although cross-platform sampling may, to some extent, enhance the generalizability of the findings, different platforms may vary in their algorithmic rules, task allocation mechanisms, performance evaluation methods, and worker management practices, and such differences may further shape platform workers’ perceptions of algorithmic control and their behavioral responses. At the same time, platform management practices, labor norms, and platform workers’ acceptance of algorithmic control may also differ across cultural contexts; therefore, the findings of this study may not be directly generalizable to Western platform settings. In addition, although the sample was drawn from different regions and different types of platforms across China, differences in platform management practices and local regulatory policies may have introduced unobserved heterogeneity. Moreover, ride-hailing and instant delivery platform work is characterized by strong time sensitivity, routinization, and algorithm dependence, which differs substantially from remote forms of platform labor such as freelance writing and programming. Future research could conduct cross-cultural comparisons, examine the algorithm governance characteristics of different platforms, carry out more context-specific investigations on particular platforms, or compare platform labor across countries, platform types, and task attributes (e.g., freelance writing or programming), so as to further test the boundary conditions of the present findings.

Second, this study was based primarily on survey data and supplemented by a scenario-based experiment, but overall it still relied largely on stage-based data and thus provided only limited insight into the dynamic evolution of the relationships among the variables. In particular, although Study 1 adopted a two-wave time-lagged design and controlled for common method bias using Harman’s single-factor test and a common latent factor approach, PAC and AAV were still measured in the same wave at Time 1, which means that potential common method variance cannot be fully ruled out. Future research could adopt longitudinal designs with longer time spans and more finely separated waves, measuring the independent variable and the moderator at different time points, in order to further reduce common method bias and more deeply examine the evolving relationships among PAC, WO, and SVCB, thereby improving the identification of causal relationships and dynamic mechanisms.

Third, although Study 2 adopted a scenario-based experimental method and thus strengthened, to some extent, the identification of the relationship between PAC and WO, the manipulation relied mainly on brief textual scenarios and therefore still had limited ability to reproduce real platform labor settings. In real platform work, algorithmic control is typically accompanied by continuous time pressure, dynamic task changes, performance feedback, and reward-and-punishment mechanisms, and these complex cues are difficult to fully simulate through a one-time reading task. Therefore, the evidence provided by Study 2 offers relatively direct support for the relationship between PAC and WO, but more complex and sustained experiences of algorithmic management still require further examination. Future research could adopt richer experimental manipulations, such as real-time task simulations, dynamic order-taking scenarios, or virtual reality (VR)-based designs, so as to more realistically reproduce the algorithmic pressure and interaction processes involved in platform labor and thereby enhance the realism and ecological validity of the experimental context.

Fourth, this study did not measure WO and organizational dehumanization simultaneously within the same research design, but only distinguished them theoretically. Therefore, it cannot directly test their empirical boundary in the context of algorithmic management. Future research could include both WO and organizational dehumanization and use discriminant validity tests, incremental validity tests, competing model comparisons, and analyses of differentiated mechanisms to further clarify the relationship and distinction between the two constructs in the context of platform algorithmic management. For example, future studies could examine whether WO mainly explains workers’ experience of understanding themselves as tools, components, or functional units within the system, whereas organizational dehumanization mainly explains workers’ perception that the organization denies their emotions, agency, and human attributes. In this way, future research could reveal more precisely how different negative psychological experiences operate in the process through which algorithmic management affects platform workers’ behavior.

Fifth, this study measured AAV using a three-item scale, and the scale showed acceptable internal consistency (Cronbach’s α = 0.796). However, the relatively small number of items may limit the breadth of the construct and the precision of the measurement. Future research could employ more comprehensive measurement instruments to better capture the multidimensional structure of AAV, including its cognitive, emotional, and behavioral aspects. Furthermore, although measuring AAV at Time 1 in Study 1 and before the manipulation in Study 2 is methodologically reasonable in terms of temporal precedence, this study did not examine whether AAV itself might be influenced by prior PAC experiences. Future research should adopt longitudinal designs to rule out the possibility that prior PAC experiences may shape AAV over time, thereby further clarifying the causal direction between the two variables.

Sixth, there remains room to further refine the conceptualization of AAV. Based on Trait Activation Theory, this study treated AAV as a relatively stable individual trait and confirmed its moderating role in the pathway through which PAC exerted its effects. However, AAV may also possess certain context-dependent or dynamically evolving characteristics. As platform workers gain more order-taking experience or become more familiar with algorithmic rules, their negative orientation toward algorithms may change. Future research could adopt longitudinal designs to examine the dynamic evolution of AAV over time and its long-term effects on responses to algorithmic management. In addition, this study examined only the moderating role of AAV. Future research could further introduce other individual characteristics and organizational-context variables, such as surface compliance (i.e., outwardly compliant behavior displayed by individuals who may not genuinely endorse organizational rules or managerial requirements internally, but comply in order to avoid punishment or maintain surface-level conformity) and platform support (i.e., the degree to which platform workers perceive that the platform values and supports their work in terms of resource provision, communication responsiveness, rights protection, and emotional care), in order to explore how other individual differences and organizational variables may shape the effects of PAC.

## Conclusion

This study, grounded in objectification theory and trait activation theory, examines how PAC leads to WO and subsequently inhibits platform workers’ SVCB, using a multi-wave survey and scenario experiment. The research further reveals that for workers with high AAV, the negative effects of PAC are more pronounced. The findings suggest that platform managers should consider workers’ attitudes towards algorithms in their algorithmic governance, fostering communication and interaction with workers to mitigate the negative impact of PAC. Overall, this study not only deepens the theoretical understanding of SVCB but also provides valuable insights for the management of PAC, laying a theoretical foundation for future developments in algorithmic management.

## Supporting information

S1 Appendix Lists of measures. (DOC)

S2 Appendix Exact p-values for Table 2 in Study 1. (DOCX)

S3 Appendix Exact p-values for Table 3 in Study 1. (DOCX)

S4 Appendix Original experiment stimuli in Study 2. (DOC)

S5 Appendix Exact p-values for Table 7 in Study 2. (DOCX)

## References

[1] Kellogg KC, Valentine MA, Christin A. Algorithms at work: The new contested terrain of control. Academy of Management Annals. 2020;14(1):366–410. doi: 10.5465/annals.2018.0174

[2] Vallas S, Schor JB. What Do Platforms Do? Understanding the Gig Economy. Annu Rev Sociol. 2020;46(1):273–94. doi: 10.1146/annurev-soc-121919-054857

[3] Lund S, Madgavkar A, Manyika J, Smit S, Ellingrud K, Robinson O. The future of work after COVID-19. 2021. https://www.mckinsey.com/featured-insights/future-of-work/the-future-of-work-after-covid-19

[4] Xiao Y, Zhu X, Kong D, Zhang J. Gig economy and corporate innovation: Evidence from the leading online delivery platform in China. In: 2025. https://ssrn.com/abstract=5225826

[5] Whalley J, Stocker V, Lutz C. A platform for doers? Fiverr and the gig economy. 2024. https://ssrn.com/abstract=4800456

[6] Rafélis de Broves O, Kang M, Grohmann R, Barcellos V, Gomes Mano F, Yoon C, et al. Workers organizing in the platform economy: Local forms and global trends of collective action. Sociology Compass. 2024;18(2). doi: 10.1111/soc4.13188

[7] Pu X. The Realization of Cooperation between Internet Platform Enterprises and Flexible Employees. Advances in Social Science, Education and Humanities Research. Atlantis Press SARL. 2023:45–52. doi: 10.2991/978-2-38476-116-6_6

[8] Yao G, Miao J. Service Value Co-Creation in Digital Platform Business: A Case of Xianyu Idle Trading Platform. Sustainability. 2021;13(20):11296. doi: 10.3390/su132011296

[9] Nadeem W, Juntunen M, Shirazi F, Hajli N. Consumers’ value co-creation in sharing economy: The role of social support, consumers’ ethical perceptions and relationship quality. Technological Forecasting and Social Change. 2020;151:119786. doi: 10.1016/j.techfore.2019.119786

[10] Möhlmann M, Zalmanson L, Henfridsson O, Gregory RW. Algorithmic Management of Work on Online Labor Platforms: When Matching Meets Control. MIS Quarterly. 2021;45(4):1999–2022. doi: 10.25300/misq/2021/15333

[11] Zhang TC, Jahromi MF, Kizildag M. Value co-creation in a sharing economy: The end of price wars? International Journal of Hospitality Management. 2018;71:51–8. doi: 10.1016/j.ijhm.2017.11.010

[12] Al-Kumaim NH, Alhazmi AK, Ramayah T, Shabbir MS, Gazem NA. Sustaining Continuous Engagement in Value Co-creation Among Individuals in Universities Using Online Platforms: Role of Knowledge Self-Efficacy, Commitment and Perceived Benefits. Front Psychol. 2021;12:637808. doi: 10.3389/fpsyg.2021.637808 33643168 PMC7907507

[13] Wiener M, Cram WA, Benlian A. Algorithmic control and gig workers: a legitimacy perspective of Uber drivers. European Journal of Information Systems. 2021;32(3):485–507. doi: 10.1080/0960085x.2021.1977729

[14] Liu S, Pei J, Ge C, Liu X, Chen Y. Algorithmic management of online labor platforms: theoretical exploration and research prospect. J Manag World. 2022;38:225–39.

[15] Wood AJ, Graham M, Lehdonvirta V, Hjorth I. Good Gig, Bad Gig: Autonomy and Algorithmic Control in the Global Gig Economy. Work Employ Soc. 2019;33(1):56–75. doi: 10.1177/0950017018785616 30886460 PMC6380453

[16] Schuilenburg M, Peeters R. The algorithmic society: Technology, power, and knowledge. London/New York: Routledge. 2021.

[17] Wu T-J, Zhang R-X, Li J-M. When employees meet digital-intelligence transformation: Unveiling the role of employee intentions. International Journal of Information Management. 2025;84:102912. doi: 10.1016/j.ijinfomgt.2025.102912

[18] Lin Q, Sun R, Zhu Q. Perceived algorithmic control and gig workers’ work engagement: assessing the mediating role of psychological empowerment and the moderating effect of deep acting. BMC Psychol. 2025;13(1):1237. doi: 10.1186/s40359-025-03570-7 41204295 PMC12595896

[19] Calogero RM. Objects don’t object: evidence that self-objectification disrupts women’s social activism. Psychol Sci. 2013;24(3):312–8. doi: 10.1177/0956797612452574 23341162

[20] Saguy T, Quinn DM, Dovidio JF, Pratto F. Interacting like a body: objectification can lead women to narrow their presence in social interactions. Psychol Sci. 2010;21(2):178–82. doi: 10.1177/0956797609357751 20424041

[21] Baldissarri C, Andrighetto L, Gabbiadini A, Volpato C. Work and freedom? Working self-objectification and belief in personal free will. Br J Soc Psychol. 2017;56(2):250–69. doi: 10.1111/bjso.12172 27862021

[22] Nussbaum MC. Objectification. Philosophy & Public Affairs. 1995;24(4):249–91. doi: 10.1111/j.1088-4963.1995.tb00032.x

[23] Haslam N. Dehumanization: An integrative review. Personality and Social Psychology Review. 2006;10(3):252–64. doi: 10.1207/s15327957pspr1003_4 16859440

[24] Bell CM, Khoury C. Organizational de/humanization, deindividuation, anomie, and in/justice. In: Gilliland S, Steiner D, Skarlicki D, editors. Emerging Perspectives on Organizational Justice and Ethics. Charlotte (NC): Information Age Publishing. 2011: 167–97.

[25] Belmi P, Schroeder J. Human “resources”? Objectification at work. J Pers Soc Psychol. 2021;120(2):384–417. doi: 10.1037/pspi0000254 32658521

[26] Crone L, Brunel L, Auzoult L. Validation of a Perception of Objectification in the Workplace Short Scale (POWS). Front Psychol. 2021;12:651071. doi: 10.3389/fpsyg.2021.651071 34163402 PMC8215106

[27] Karasek RA join('’. Job Demands, Job Decision Latitude, and Mental Strain: Implications for Job Redesign. Administrative Science Quarterly. 1979;24(2):285. doi: 10.2307/2392498

[28] Dietvorst BJ, Simmons JP, Massey C. Algorithm aversion: people erroneously avoid algorithms after seeing them err. J Exp Psychol Gen. 2015;144(1):114–26. doi: 10.1037/xge0000033 25401381

[29] Huq AZ. The waning democratic allure of digital technologies. Political Science Quarterly. 2025;qqaf121. doi: 10.1093/psquar/qqaf121

[30] Le C, Wang Z, Kong W. Algorithm appreciation vs. algorithm aversion? A study of user algorithm paradox under short video intelligent recommendation. Journal of Intelligence. 2024;43(08):170–81.

[31] Jussupow E, Benbasat I, Heinzl A. Why are we averse towards algorithms? A comprehensive literature review on algorithm aversion. In: Proceedings of the 28th European Conference on Information Systems (ECIS), 2020. https://aisel.aisnet.org/ecis2020_rp/168

[32] Tett RP, Burnett DD. A personality trait-based interactionist model of job performance. J Appl Psychol. 2003;88(3):500–17. doi: 10.1037/0021-9010.88.3.500 12814298

[33] Pei J, Liu S, Cui X, Qu J. Perceived algorithmic control of gig workers: Conceptualization, measurement and verification of the impact on service performance. Chinese J Nankai Business Review. 2021;24(6):14–25.

[34] Ryan R, Deci E. Intrinsic and Extrinsic Motivations: Classic Definitions and New Directions. Contemp Educ Psychol. 2000;25(1):54–67. doi: 10.1006/ceps.1999.1020 10620381

[35] Shin D, Park YJ. Role of fairness, accountability, and transparency in algorithmic affordance. Computers in Human Behavior. 2019;98:277–84. doi: 10.1016/j.chb.2019.04.019

[36] Tierney P, Farmer SM. Creative self-efficacy development and creative performance over time. J Appl Psychol. 2011;96(2):277–93. doi: 10.1037/a0020952 20954756

[37] Johnson JV, Hall EM. Job strain, work place social support, and cardiovascular disease: a cross-sectional study of a random sample of the Swedish working population. Am J Public Health. 1988;78(10):1336–42. doi: 10.2105/ajph.78.10.1336 3421392 PMC1349434

[38] Duan W-Y, Wu T-J, Liang Y. Are resources always beneficial? The three-way interaction between slashies’ role stress, self-efficacy and job autonomy. Asia Pac J Manag. 2025;43(1):613–43. doi: 10.1007/s10490-025-10027-3

[39] Guo Y, Li R, Xia L-X. Effects of relative deprivation on change in displaced aggression and the underlying motivation mechanism: A three-wave cross-lagged analysis. Br J Psychol. 2024;115(1):1–19. doi: 10.1111/bjop.12674 37351801

[40] Ramírez-Montoya M-S, García-Peñalvo F-J. Co-creation and open innovation: Systematic literature review. Comunicar: Revista Científica de Comunicación y Educación. 2018;26(54):09–18. doi: 10.3916/c54-2018-01

[41] Rosenblat A, Stark L. Algorithmic labor and information asymmetries: A case study of Uber’s drivers. International Journal of Communication. 2016;10:3758–84. doi: 10.2139/ssrn.2686227

[42] Amin M, Khan I, Shamim A, Ting DH, Jan A, Abbasi AZ. Employee motivations in shaping customer value co-creation attitude and behavior: Job position as a moderator. Journal of Retailing and Consumer Services. 2024;79:103819. doi: 10.1016/j.jretconser.2024.103819

[43] Pathak B, Ashok M, Leng Tan Y. Value co-creation in the B2B context: a conceptual framework and its implications. The Service Industries Journal. 2021;42(3–4):178–205. doi: 10.1080/02642069.2021.1989414

[44] Chen N, Hu M, Li W. Algorithmic decision-making safeguarded by human knowledge. ArXiv. 2022. https://arxiv.org/abs/2211.11028

[45] Huang Y, Cao X. Calling on the Third-party Privacy Control into Algorithmic Governance Framework: Linking Users’ Presumed Influence with Control Agency Theory. International Journal of Public Opinion Research. 2023;35(4). doi: 10.1093/ijpor/edad036

[46] Baldissarri C, Valtorta RR, Andrighetto L, Volpato C. Workers as objects: The nature of working objectification and the role of perceived alienation. TPM: Testing, Psychometrics, Methodology in Applied Psychology. 2017;24(2):156–66. doi: 10.4473/TPM24.2.1

[47] Murtza MH, Rasheed MI. The dark side of competitive psychological climate: exploring the role of workplace envy. JHTI. 2022;6(3):1400–18. doi: 10.1108/jhti-03-2022-0097

[48] You S, Yang CL, Li X. Algorithmic versus Human Advice: Does Presenting Prediction Performance Matter for Algorithm Appreciation? Journal of Management Information Systems. 2022;39(2):336–65. doi: 10.1080/07421222.2022.2063553

[49] Chen L. Labor order under digital control: research on labor control of take-out platform riders. J Chin Sociol. 2022;9(1). doi: 10.1186/s40711-022-00171-4

[50] Ranjan KR, Read S. Value co-creation: concept and measurement. J of the Acad Mark Sci. 2014;44(3):290–315. doi: 10.1007/s11747-014-0397-2

[51] Baldissarri C, Andrighetto L. Being Treated as an Instrument: Consequences of Instrumental Treatment and Self-Objectification on Task Engagement and Performance. Human Performance. 2021;34(2):85–106. doi: 10.1080/08959285.2021.1878182

[52] Fredrickson BL, Roberts T-A. Objectification Theory: Toward Understanding Women’s Lived Experiences and Mental Health Risks. Psychology of Women Quarterly. 1997;21(2):173–206. doi: 10.1111/j.1471-6402.1997.tb00108.x

[53] Poon K-T, Chen Z, Teng F, Wong W-Y. The effect of objectification on aggression. Journal of Experimental Social Psychology. 2020;87:103940. doi: 10.1016/j.jesp.2019.103940

[54] Schaufeli WB, Salanova M, González-romá V, Bakker AB. The Measurement of Engagement and Burnout: A Two Sample Confirmatory Factor Analytic Approach. Journal of Happiness Studies. 2002;3(1):71–92. doi: 10.1023/a:1015630930326

[55] Calogero RM, Tantleff-Dunn SE, Thompson J. Objectification theory: An introduction. Self-objectification in Women: Causes, Consequences, and Counteractions. Washington, DC: American Psychological Association. 2011:3–21.

[56] Zou Y, Zhang H, Peng J, Nie Q, Wang Z. Change or procrastination? Employees’ differentiated responses to illegitimate tasks. Acta Psychologica Sinica. 2023;55(9):1529. doi: 10.3724/sp.j.1041.2023.01529

[57] Liang Y, Wu T-J, Lin W. Exploring the impact of forced teleworking on counterproductive work behavior: the role of event strength and work-family conflict. Internet Research. 2024;36(1):53–77. doi: 10.1108/intr-08-2023-0658

[58] Longoni C, Bonezzi A, Morewedge CK. Resistance to Medical Artificial Intelligence. Journal of Consumer Research. 2019;46(4):629–50. doi: 10.1093/jcr/ucz013

[59] Du M, Wu D, Wang L, Guo X, Deng H, Zhuang X. The impact of algorithm dysfunction on human-algorithm collaboration. In: Kuala Lumpur, Malaysia, 2025. Article 23. https://aisel.aisnet.org/pacis2025/aiandml/aiandml/23

[60] Wang W, Chen C-HS, Nguyen B, Shukla P. Collaboration between East and West: influence of consumer dialectical self on attitude towards co-brand personality traits. IMR. 2020;37(6):1155–80. doi: 10.1108/imr-01-2019-0012

[61] Podsakoff PM, MacKenzie SB, Lee JY, Podsakoff NP. Common method biases in behavioral research: a critical review of the literature and recommended remedies. Journal of Applied Psychology. 2003;88(5):879–903. doi: 10.1037/0021-9010.88.5.879 14516251

[62] Tu K, Yang X, Su X, Ou X. The influence of supplier user’s role stress on continuous value co-creation behavior in the sharing economy: A mediated moderation model. Chinese J Nankai Business Review. 2020;23:88–98.

[63] Lang J, Cheng C, Li M. The Impact of Perceived Algorithmic Control on Employee Work Engagement: the mediating role of Burnout. In: 2022 13th International Conference on E-business, Management and Economics, 2022. 163–8. 10.1145/3556089.3556164

[64] Son S, Kim D-Y. Organizational career growth and career commitment: Moderated mediation model of work engagement and role modeling. The International Journal of Human Resource Management. 2019;32(20):4287–310. doi: 10.1080/09585192.2019.1657165

[65] Hayes AF. Introduction to mediation, moderation, and conditional process analysis: A regression-based approach. 2 ed. New York, NY: Guilford Publications. 2017.

[66] Muthén B, Muthén L. Mplus. In: Van der Linden WJ, editor. Handbook of Item Response Theory. Boca Raton, FL: Chapman and Hall/CRC. 2017:507–18.

[67] Fornell C, Larcker DF. Evaluating Structural Equation Models with Unobservable Variables and Measurement Error. Journal of Marketing Research. 1981;18(1):39. doi: 10.2307/3151312

[68] Aiken LS, West SG. Multiple regression: Testing and interpreting interactions. Newbury Park, CA: SAGE Publications. 1991.

[69] Preacher KJ, Curran PJ, Bauer DJ. Computational Tools for Probing Interactions in Multiple Linear Regression, Multilevel Modeling, and Latent Curve Analysis. Journal of Educational and Behavioral Statistics. 2006;31(4):437–48. doi: 10.3102/10769986031004437

[70] Faul F, Erdfelder E, Buchner A, Lang A-G. Statistical power analyses using G*Power 3.1: tests for correlation and regression analyses. Behav Res Methods. 2009;41(4):1149–60. doi: 10.3758/BRM.41.4.1149 19897823

[71] Wang H, Chen Z, Li Z, Liu Z, Liang C, Zhao B. How to break out of time dilemma: The subjective time boundaries for the effects of algorithmic control on gig workers. Acta Psychologica Sinica. 2025;57(2):275. doi: 10.3724/sp.j.1041.2025.0275

[72] Casu G, Mariani MG, Chiesa R, Guglielmi D, Gremigni P. The Role of Organizational Citizenship Behavior and Gender between Job Satisfaction and Task Performance. Int J Environ Res Public Health. 2021;18(18):9499. doi: 10.3390/ijerph18189499 34574423 PMC8469399

[73] XXX.

[74] Fardouly J, Diedrichs PC, Vartanian LR, Halliwell E. Social comparisons on social media: the impact of Facebook on young women’s body image concerns and mood. Body Image. 2015;13:38–45. doi: 10.1016/j.bodyim.2014.12.002 25615425

[75] Gruenfeld DH, Inesi ME, Magee JC, Galinsky AD. Power and the objectification of social targets. J Pers Soc Psychol. 2008;95(1):111–27. doi: 10.1037/0022-3514.95.1.111 18605855

[76] Lee I, Shin YJ. Machine learning for enterprises: Applications, algorithm selection, and challenges. Business Horizons. 2020;63(2):157–70. doi: 10.1016/j.bushor.2019.10.005

[77] Tyler TR, Lind EA. A Relational Model of Authority in Groups. Advances in Experimental Social Psychology. Elsevier. 1992:115–91. doi: 10.1016/s0065-2601(08)60283-x

