# Supplementary material for: How to break free from the "tool" dilemma? A study on the impact of perceived algorithmic control on sustainable value co-creation behavior
Source: PLoS One. 2026 Sep 8;21(9):e0357097. doi: 10.1371/journal.pone.0357097 (PMC13552789; doi:10.1371/journal.pone.0357097)
Supplement: Supplementary file 1 [file pone.0357097.s002.docx]

**S2 Appendix. Exact p-values for Table 2 in Study 1**

**Exact p-values for the correlation matrix in Table 2**

| **Variables** | M | SD | 1 | | 2 | | 3 | | 4 | | 5 | | 6 | | 7 | | 8 | |
| --- | --- | --- | --- | --- | --- | --- | --- | --- | --- | --- | --- | --- | --- | --- | --- | --- | --- | --- |
|  |  |  | r | p | r | p | r | p | r | p | r | p | r | p | r | p | r | p |
| 1.Gender | 1.380 | 0.531 |  |  |  |  |  |  |  |  |  |  |  |  |  |  |  |  |
| 2.Age | 2.610 | 0.702 | -0.068 | 0.255 |  |  |  |  |  |  |  |  |  |  |  |  |  |  |
| 3.Education Level | 3.580 | 1.023 | 0.149 | 0.012 | -0.254 | P<0.001 |  |  |  |  |  |  |  |  |  |  |  |  |
| 4.Work Experience | 3.480 | 1.555 | -0.065 | 0.275 | 0.580 | P<0.001 | -0.307 | P<0.001 |  |  |  |  |  |  |  |  |  |  |
| 5.Job Type | 1.260 | 0.437 | -0.077 | 0.193 | 0.467 | 0.012 | -0.280 | P<0.001 | 0.460 | P<0.001 |  |  |  |  |  |  |  |  |
| 6.PAC | 3.650 | 0.651 | 0.034 | 0.569 | -0.247 | P<0.001 | 0.333 | P<0.001 | -0.266 | P<0.001 | -0.280 | P<0.001 |  |  |  |  |  |  |
| 7.WO | 3.320 | 0.911 | -0.066 | 0.266 | -0.255 | P<0.001 | 0.268 | P<0.001 | -0.255 | P<0.001 | -0.243 | P<0.001 | 0.421 | P<0.001 |  |  |  |  |
| 8.AAV | 2.990 | 0.912 | -0.054 | 0.365 | 0.004 | 0.945 | -0.018 | 0.769 | -0.004 | 0.949 | 0.005 | 0.929 | 0.243 | P<0.001 | 0.133 | 0.025 |  |  |
| 9.SVCB | 2.420 | 0.874 | 0.131 | 0.028 | 0.172 | 0.004 | -0.099 | 0.096 | 0.171 | 0.004 | 0.146 | 0.014 | -0.344 | P<0.001 | -0.499 | P<0.001 | -0.193 | P<0.001 |
| N = 285；p< 0.05，p < 0.01. Gender: male=1, female=2; Age: under 20 years old=1, 21-30 years old=2, 31-40 years old=3, 41-50 years old=4, over 50 years old=5;Education: primary school and below=1,junior high school =2, general high school/vocational school/technical school/vocational high school=3, junior college=4, bachelor's degree or above=5; Work experience: within 1 year=1, 1-3 years=2, 3-5 years=3, 5-7 years=4, 7-9 years=5, 9 years and above = 6; Job type: full time=1, part-time=2 | | | | | | | | | | | | | | | | | | |
