# Supplementary material for: How to break free from the "tool" dilemma? A study on the impact of perceived algorithmic control on sustainable value co-creation behavior
Source: PLoS One. 2026 Sep 8;21(9):e0357097. doi: 10.1371/journal.pone.0357097 (PMC13552789; doi:10.1371/journal.pone.0357097)
Supplement: Supplementary file 2 [file pone.0357097.s003.docx]

| **Exact p-values corresponding to the hierarchical regression analysis in Table 3** | | | | | | | | | | | | | | | | | | |
| --- | --- | --- | --- | --- | --- | --- | --- | --- | --- | --- | --- | --- | --- | --- | --- | --- | --- | --- |
| **Variables** | **WO** | | | | | | | | | **SVCB** | | | | | | | | |
|  | **M1** | | | **M2** | | | **M3** | | | **M4** | | | **M5** | | | **M6** | | |
|  | **β** | **SE** | **p-Value** | **β** | **SE** | **p-Value** | **β** | **SE** | **p-Value** | **β** | **SE** | **p-Value** | **β** | **SE** | **p-Value** | **β** | **SE** | **p-Value** |
| **Control Variables** | |  |  |  |  |  |  |  |  |  |  |  |  |  |  |  |  |  |
| Gender | -0.117 | 0.105 | 0.038 | -0.205 | 0.100 | 0.042 | -0.198 | 0.098 | 0.044 | 0.155 | 0.105 | 0.009 | 0.265 | 0.101 | 0.009 | 0.182 | 0.093 | 0.051 |
| Age | -0.113 | 0.092 | 0.114 | -0.116 | 0.087 | 0.185 | -0.116 | 0.086 | 0.176 | 0.92 | 0.1092 | 0.215 | 0.087 | 0.088 | 0.325 | 0.040 | 0.081 | 0.624 |
| Education Level | 0.201 | 0.053 | P<0.001 | 0.104 | 0.052 | 0.047 | 0.103 | 0.051 | 0.046 | -0.056 | 0.053 | 0.366 | 0.021 | 0.052 | 0.683 | 0.063 | 0.048 | 0.191 |
| Work Experience | -0.088 | 0.042 | 0.219 | -0.035 | 0.040 | 0.380 | -0.039 | 0.039 | 0.322 | 0.82 | 0.042 | 0.275 | 0.030 | 0.040 | 0.450 | 0.016 | 0.037 | 0.661 |
| Job Type | -0.102 | 0.137 | 0.121 | -0.117 | 0.131 | 0.371 | -0.147 | 0.129 | 0.254 | 0.061 | 0.137 | 0.372 | 0.034 | 0.132 | 0.799 | -0.014 | 0.121 | 0.909 |
| **Independent Variable** | |  |  |  |  |  |  |  |  |  |  |  |  |  |  |  |  |  |
| PAC |  |  |  | 0.466 | 0.081 | P<0.001 | 0.553 | 0.088 | P<0.001 |  |  |  | -0.431 | 0.081 | P<0.001 | -0.243 | 0.079 | 0.002 |
| **Mediating Variable** | |  |  |  |  |  |  |  |  |  |  |  |  |  |  |  |  |  |
| WO |  |  |  |  |  |  |  |  |  |  |  |  |  |  |  | -0.405 | 0.093 | P<0.001 |
| **Moderating Variable** | |  |  |  |  |  |  |  |  |  |  |  |  |  |  |  |  |  |
| AAV |  |  |  |  |  |  | 0.004 | 0.056 | 0.939 |  |  |  |  |  |  |  |  |  |
| Interaction Term | |  |  |  |  |  |  |  |  |  |  |  |  |  |  |  |  |  |
| PAC × AAV |  |  |  |  |  |  | 0.279 |  | 0.001 |  |  |  |  |  |  |  |  |  |
| R2 | 0.138 |  |  | 0.230 |  |  | 0.265 |  |  | 0.065 |  |  | 0.141 |  |  | 0.283 |  |  |
| ΔR2 | 0.138 |  |  | 0.093 |  |  | 0.033 |  |  | 0.065 |  |  | 0.085 |  |  | 0.142 |  |  |
| F | 8.910 |  |  | 13.860 |  | P<0.001 | 12.468 |  | P<0.001 | 3.850 |  | P<0.001 | 7.619 |  |  | 15.615 |  | P<0.001 |

**S3 Appendix. Exact p-values for Table 3 in Study 1**
