# Supplementary material for: How to break free from the "tool" dilemma? A study on the impact of perceived algorithmic control on sustainable value co-creation behavior
Source: PLoS One. 2026 Sep 8;21(9):e0357097. doi: 10.1371/journal.pone.0357097 (PMC13552789; doi:10.1371/journal.pone.0357097)
Supplement: Supplementary file 3 [file pone.0357097.s005.docx]

**S5 Appendix. Exact p-values for Table 7 in Study 2**

**Exact p-values corresponding to the hierarchical regression analysis in Table 7**

| **Variables** | **WO** | | | | | | | | |
| --- | --- | --- | --- | --- | --- | --- | --- | --- | --- |
|  | **M1** | | | **M2** | | | **M3** | | |
|  | **β** | **SE** | **p-Value** | **β** | **SE** | **p-Value** | **β** | **SE** | **p-Value** |
| **Control Variables** |  |  |  |  |  |  |  |  |  |
| Gender | -0.139 | 0.043 | 0.037 | -0.069 | 0.040 | 0.086 | -0.048 | 0.040 | 0.227 |
| Age | 0.253 | 0.038 | P<0.001 | -0.070 | 0.037 | 0.061 | 0.061 | 0.036 | 0.096 |
| Education level | 0.128 | 0.043 | 0.054 | 0.019 | 0.040 | 0.635 | 0.024 | 0.040 | 0.549 |
| Work experience | 0.195 | 0.033 | 0.004 | 0.054 | 0.031 | 0.084 | 0.044 | 0.031 | 0.149 |
| Daily working hours | -0.15 | 0.034 | 0.820 | 0.017 | 0.031 | 0.592 | -0.001 | 0.031 | 0.974 |
| **Independent Variable** |  |  |  |  |  |  |  |  |  |
| PAC |  |  |  | 0.502 | 0.078 | P<0.001 | 0.435 | 0.083 | P<0.001 |
| **Moderating Variable** |  |  |  |  |  |  |  |  |  |
| AAV |  |  |  |  |  |  | 0.089 | 0.045 | 0.048 |
| **Interaction Term** |  |  |  |  |  |  |  |  |  |
| PAC × AAV |  |  |  |  |  |  | 0.195 | 0.090 | 0.032 |
| R^2^ | 0.116 |  |  | 0.263 |  |  | 0.297 |  |  |
| ΔR^2^ | 0.116 |  |  | 0.147 |  |  | 0.181 |  |  |
| F | 5.529 |  | P<0.001 | 12.401 |  | P<0.001 | 10.936 |  | P<0.001 |
