# Supplementary material for: How to break free from the "tool" dilemma? A study on the impact of perceived algorithmic control on sustainable value co-creation behavior
Source: PLoS One. 2026 Sep 8;21(9):e0357097. doi: 10.1371/journal.pone.0357097 (PMC13552789; doi:10.1371/journal.pone.0357097)
Supplement: Supplementary file 4 [file pone.0357097.s008.docx]

**Response to reviewers**

**Reviewer #1:** Thanks for allowing me to review the paper How to break free from the "tool" dilemma? A study on the impact of perceived algorithmic control on sustainable value co-creation behavior. This is a well-designed and timely study with strong theoretical and practical contributions. With moderate revisions addressing some points—particularly around conceptual clarity, generalizability, and experimental validity—it would be suitable for publication in PLOS ONE.

**Comments to the author:**

1.Conceptual Clarity and Theoretical Contribution
WO vs. Organizational Dehumanization: While the distinction is noted (lines 65–66, 482–485), it could be strengthened. Clarify how WO (instrumentality, disposability) uniquely applies to gig work versus general dehumanization constructs. A brief comparative analysis in the discussion would help. (e.g. Are resources always beneficial? The three-way interaction between slashies’ role stress, self-efficacy and job autonomy. Asia Pacific Journal of Management, 2025, 1-31.)
AAV as a Trait vs. State: The study treats AAV as a stable trait, but algorithmic aversion may be context-dependent or learnable. Acknowledge this limitation and suggest future research on AAV development over time. (e.g. The buffering role of workplace mindfulness: How job insecurity of human-artificial intelligence collaboration impacts employees’ work–life-related outcomes. Journal of Business and Psychology, 2024, 39.6: 1395-1411.)

**Authors’ response:**

We sincerely thank the reviewer for the constructive comments regarding conceptual clarity and theoretical refinement. In response to these suggestions, we have carefully revised the manuscript to further strengthen the theoretical distinction between workplace objectification (WO) and organizational dehumanization, and to provide a more cautious and precise conceptualization of algorithm aversion (AAV).

First, regarding the distinction between workplace objectification and organizational dehumanization, we have added a clearer theoretical differentiation in the Introduction (Lines 58–86) to explain why workplace objectification is more appropriate for capturing the psychological experience of platform workers under algorithmic control. We also revised the *Discussion section* (Lines 664–678) to provide a brief comparison between the two constructs. In addition, we clarified this issue in the *Limitations and Future Directions section* (Lines 834–841) by acknowledging the related limitation. Specifically, we clarified that organizational dehumanization refers to a broader perception that the organization denies employees’ human qualities, whereas workplace objectification focuses on the experience of being treated as an instrumental tool for task execution, which is particularly salient in algorithmically managed gig work.

Second, regarding the conceptualization of algorithm aversion, we carefully considered the reviewer’s point that this construct may be context-dependent and potentially learnable. Accordingly, we refined the definition of algorithm aversion in the Introduction (Lines 87–114) to make it more precise. We also added discussion in the *Limitations and Future Directions section* (Lines 848–864), where we acknowledge the context-dependent nature of algorithm aversion and call for future research to adopt longitudinal designs or experience sampling methods to examine its dynamic evolution, as well as whether its moderating role may vary across different stages of algorithm adaptation.

The relevant revisions have been highlighted in yellow in the manuscript. Thank you again for your valuable suggestion.

**Comments to the author:**

2. Sample and Generalizability
The sample is limited to Chinese platform workers (e.g., Didi, Meituan). While valuable, findings may not generalize to Western platforms or non-delivery/ride-hailing gig work (e.g., freelance writing, coding). Please confirm this cultural and platform-specific limitation.
Most participants were male (62.1%) and aged 21–40. Consider discussing how gender/age might influence PAC, WO, or SVCB, or control for them more explicitly.

**Authors’ response:**

We have revised the manuscript accordingly. In fact, both Study 1 and Study 2 were based exclusively on Chinese platform workers (e.g., workers from DiDi and Meituan). We have now clarified in the *Sample and Procedure section* of Study 1 (Lines 291–292), as well as in the *Design and Participants section* of Study 2 (Line 490-491), that the samples in this research focus on Chinese ride-hailing and food delivery platform workers. We have also added a corresponding statement in the *Limitations and Future Directions section* (Lines 788–808) to acknowledge the limitations related to the sample scope of the present study.

In addition, we agree that demographic variables such as gender and age may introduce potential bias into the results. To address this concern, we made two revisions. First, we re-ran the regression analyses using a hierarchical regression approach (see the revised Tables 3 and 7 for details). In Model 1 and Model 4, we included all demographic variables (e.g., gender, age, and education) as control variables. The results show that, after controlling for these demographic factors, the effects of perceived algorithmic control (PAC) on workplace objectification (WO) and sustainable value co-creation behavior (SVCB) remained highly significant. Second, we added brief interim discussions at the end of Study 1 (Lines 448–477) and Study 2 (Lines 602–620), respectively, to further interpret the findings at each stage.

The relevant revisions have been highlighted in yellow in the manuscript. Thank you for your suggestion.

**Comments to the author:**

3.Measurement and Common Method Bias
Although Harman’s test and CLF were used (lines 315–324), the two-wave design still collected PAC and AAV at the same time in Wave 1. Consider noting that temporal separation between PAC and AAV could further reduce CMV.
The AAV scale has only 3 items (Cronbach’s α = 0.796). While acceptable, a longer scale might improve reliability. Mention this as a minor limitation.

**Authors’ response:**

We appreciate the reviewer’s valuable comment on this issue. In fact, although PAC and AAV were both measured in the first wave during the initial questionnaire design, the survey procedure was structured in a way that helped reduce potential bias. Specifically, demographic information and algorithm aversion were measured first, whereas perceived algorithmic control was measured at the end of the questionnaire. In addition, in the instruction for the algorithm aversion measure, participants were explicitly asked to respond based on their “overall and stable feelings and attitudes toward algorithmic technology over time,” rather than on situational or momentary emotional reactions. We have added this clarification to the *Sample and Procedure section* (Lines 298–303).

At the same time, in the *Limitations and Future Directions section* (Lines 809–820), we acknowledge that collecting these variables at different time points could further reduce potential common method variance, and we suggest that future research may adopt a multi-wave design to separate the measurement of the independent variable and the moderator.

In addition, regarding the algorithm aversion (AAV) scale, which contains only three items (Cronbach’s α = 0.796), we agree that the relatively small number of items may to some extent limit construct coverage and measurement precision. Accordingly, we have added a corresponding statement in the *Limitations and Future Directions section* (Lines 842–847).

The relevant revisions have been highlighted in yellow in the manuscript. Thank you again for your valuable suggestion.

**Comments to the author:**

4.Experimental Design (Study 2)
The scenario-based manipulation of PAC (reading an article) may lack ecological validity compared to real platform interactions. Participants may not fully internalize the algorithmic control experience. Suggest future studies use simulated task platforms or VR.
The control group read an unrelated article. Ensure that the control topic is neutral and does not inadvertently trigger thoughts about algorithms or work control.

**Authors’ response:**

First, we agree that a scenario-based experimental manipulation may not fully capture the complexity and psychological intensity of real platform interactions. Therefore, we have addressed this issue in the *Limitations and Future Directions section* (Lines 821–833).

Second, regarding the control group materials, some related details were omitted in the original manuscript due to word limit constraints. In the revised manuscript, we have added a description of the development and selection process of the experimental materials in the PAC Manipulation and Pretest section (Lines 499–514). Specifically, the experimental materials were adapted from prior studies, and were further evaluated and revised through internal discussions and with the assistance of two doctoral students, in order to ensure their cultural appropriateness.

The relevant revisions have been highlighted in yellow in the manuscript. Thank you again for your valuable suggestion.

**Comments to the author:**

5. Causal Claims
While the experiment strengthens causality for PAC → WO, the mediation (WO → SVCB) and full moderated mediation model still rely on correlational survey data. Use cautious language (e.g., “supports a causal pathway” rather than “establishes causality”).

**Authors’ response:**

In response to this suggestion, we have made consistent revisions throughout the manuscript. Specifically, when referring to Study 1 and the full model, we adopted more cautious expressions to avoid overstating the findings. For Study 2, we revised the wording to indicate that it “provides more direct causal evidence for the relationship between PAC and WO,” while avoiding overly strong claims such as “proves” or “establishes causality.”

In addition, we further clarified in the discussion of Study 1, the discussion of Study 2, and the General Discussion the distinction between the scope of causal inference that can be supported by the experimental study and the correlational nature of the evidence provided by the survey study. The relevant revisions have been highlighted in the manuscript. Thank you again for your valuable suggestion.

**Comments to the author:**

6.Discussion and Implications
*Theoretical Implications*: Expand on how the study advances trait activation theory in algorithmic contexts. How does AAV interact with situational cues in gig work?

*Managerial Implications*: Provide more concrete examples: What would “transparent task allocation” look like? How can platforms implement “two-way feedback mechanisms” effectively? (e.g. When employees meet digital-intelligence transformation: Unveiling the role of employee intentions. International Journal of Information Management, 2025, 84: 102912.)
*Ethical and Policy Implications*: Briefly discuss how regulatory frameworks (e.g., EU AI Act, gig worker laws) could address PAC and WO.

**Authors’ response:**

Regarding the *theoretical implications*, we revised and enriched the relevant content in response to your suggestion. First, in the Introduction (Lines 87–113), we strengthened the logical connection among the high-demand/low-control platform labor context, algorithm aversion, and trait activation theory. We also expanded the *Theoretical Implications section* (Lines 679–702) to clarify how this study extends trait activation theory in the context of platform algorithmic management, and how algorithm aversion interacts with situational cues in gig work.

Regarding the managerial implications, we also revised and refined this part by adding more specific and actionable practical suggestions.

With respect to the ethical and policy implications, we added a fifth paragraph to the Managerial Implications section in the revised manuscript (Lines 770–786) to provide a brief discussion of these issues.

The relevant revisions have been highlighted in the manuscript. Thank you again for your valuable suggestion.

**Comments to the author:**

7.Writing and Presentation
Abstract: The abstract repeats the same sentence twice (lines 11–23). Remove the duplication.

Figures: Ensure all figures (Fig 1–4) are clearly referenced in the text. In the PDF, some figures are listed at the end without in-text callouts (e.g., “INSERT FIG HERE” placeholders remain).
References: Some refs are incomplete (e.g., ref 62 lacks title/author). Ensure all references follow PLOS ONE formatting guidelines.

Minor Corrections
Line 11–23: Remove duplicated sentence in abstract.
Line 301: “unutilized” → “used.”
Line 440–441: Table 7 note formatting is misaligned.
Line 527–528: “surface compliance or platform support” — briefly define these if they are proposed as future moderators.
Figure placement: Integrate figures into the main text where referenced.

**Authors’ response:**

We sincerely thank the reviewer for the careful examination of the manuscript’s formatting, figures, and references. We have revised each of these issues point by point in accordance with your suggestions.

We checked and standardized the in-text citations of Figures 1–4, removed placeholders such as “INSERT FIG HERE” from the original manuscript, and integrated the figures into the main text at the appropriate points of reference.

We also conducted a comprehensive review and supplementation of the references, corrected incomplete entries, and revised the reference list to conform to the formatting requirements of *PLOS ONE*.

The duplicated sentence in the abstract has been deleted.

The word “unutilized” in Line 301 has been revised.

The note format in Table 7 has been reformatted and consistently aligned.

Brief definitions of “surface compliance” and “platform support” have been added to improve clarity of expression.

The relevant revisions have been highlighted in the manuscript. Thank you again for your valuable suggestion.

**Reviewer #2:** We commend the authors on an interesting pair of studies. The question of platform workers’ well-being is important, and will only become increasingly so as more and more people engage in part-time or full-time platform work. There is no doubt that understanding how workers relate to their jobs in the platform work industry is critical. As the authors themselves are clearly aware, this understanding enables us to better intervene in how platform work is managed.
Comments are divided into major and minor recommendations. Most comments are listed in an order aligned with the paper itself, unless otherwise specified:
**Major Comments:**

Comments to the author:

1. We thank the authors for an extremely clear and coherent Introduction section. It did feel at times, however, that concepts and definitions were perhaps repeated more than is needed for readers’ understanding. Authors should feel free to disregard this recommendation, but it may be possible to streamline/shorten the Introduction section a little.

Authors’ response:

We appreciate the reviewer’s suggestion. While we retained most of the introduction to preserve conceptual clarity, we carefully reviewed the section and removed several repetitive sentences to improve conciseness.The relevant revisions have been highlighted in the manuscript.

Comments to the author:

2. One of the claims made by the authors (starting on line 481) is that “this research clarifies the conceptual distinction between WO and organizational dehumanization.” Given the scope of the studies reported here, it is not clear how this conceptual distinction has been made. It is this reviewer’s opinion that this cannot be stated as a major research contribution of the project, or, at the very least, not without additional context:
- In the Introduction, on line 65, the authors write “Unlike organizational dehumanization, which denies human uniqueness, WO emphasizes traits like instrumentality, disposability, lack of autonomy, and violability.” From this explanation alone, it is not clear how organizational dehumanization and WO are distinguished. A more detailed explanation of organization dehumanization (as opposed to WO), would be helpful, especially if it can say more about the specific features that are associated with one but not the other.
- In the discussion, as mentioned above, the authors claim that the conceptual distinction between the two has been made clear. However, in the studies itself, only WO is tested. As far as has been reported, it does not seem that any measures/scales reflecting “organizational humanization” were tested/collected in the two studies. If one claims to have distinguished between the two, then we would expect the two frameworks (WO vs. organizational dehumanization) to be pitted against each other in some manner.
- Especially to those unfamiliar with the relevant literatures, the way WO is described and operationalized can seem very much aligned with lay intuitions of organizational dehumanization.
For instance, in line 500, the authors state: “WO is particularly damaging in the gig economy, where workers may feel like mere "tools of production." This impression of being “tools of production” associated with WO seems very reminiscent of the “mechanistic” frame associated with organizational dehumanization (mentioned in line 482).
Please note that this reviewer isn’t arguing against the idea that WO and organizational dehumanization can be (at least partially) dissociated. Rather, the recommendation is to either: (1) Provide further evidence/background for how this distinction has been made, or to (2) Acknowledge that this distinction cannot yet be fully clarified by the present approach, and perhaps suggest ways this distinction could be clarified by future work.

Authors’ response:

We thank the reviewer for this important comment. We have revised the relevant statements accordingly. First, in the Introduction (Lines 58–86), we added a clearer conceptual distinction between workplace objectification (WO) and organizational dehumanization, and explained the difference in their theoretical focus. Specifically, we clarified that organizational dehumanization refers to a broader perception that the organization denies employees’ human qualities, whereas workplace objectification focuses on the experience of being treated as an instrumental tool for task execution, which is particularly salient in algorithmically managed gig work.

Second, in the *Theoretical Implications section* (Lines 664–678), we revised the original wording to more cautiously describe the applicability and explanatory value of workplace objectification in the context of platform algorithmic management. Accordingly, we revised the manuscript to avoid overstating this point as a major empirical contribution. Instead, we position the present study as revealing the relevance of workplace objectification in the context of algorithmic management.

Finally, in the *Limitations and Future Directions section* (Lines 834–841), we added a statement noting that future research could examine the relationship and distinction between these two constructs through parallel measurement and discriminant validity tests, so as to more clearly reveal their connections and differences in the context of algorithmic management.

The relevant revisions have been highlighted in the manuscript. Thank you again for your valuable suggestion.

Comments to the author:

3. A comprehensive set of analyses were run for the two studies reported here. However, it would generally be helpful to provide a little more context for these analyses, both when describing how they were done and what they tell us:
- For instance, it looks like some of the Control Variables (Gender & Education Level) were significant in some of the models tested (when running the hierarchical regression analyses - lines 341-353). While this reviewer understands that this isn’t amongst the main take-aways of this work, it would be good to acknowledge these findings, and provide some context for what they could mean.

Authors’ response:

In response to your suggestion, we supplemented the descriptions of the data analyses in both studies so that the purposes of the analytical methods and the meanings of the results are presented more clearly.

First, in the Data Analysis section (Lines 343–349), we clarified the roles of confirmatory factor analysis, common method bias testing, correlation analysis, and hierarchical regression analysis.

Second, in the hypothesis testing sections of both Study 1 and Study 2, we added brief explanations of the results for the control variables. We also added baseline models to Table 3 and Table 7, respectively.

Third, we added brief discussions at the end of Study 1 (Lines 448–477) and Study 2 (Lines 602–620), respectively, to comment on the finding that some control variables (i.e., gender and education level) appeared to reach significance in certain models.

The relevant revisions have been highlighted in the manuscript. Thank you again for your valuable suggestion.

Comments to the author:

4. It would be nice to add a short *Discussion section* at the end of Study 1, recapping the main takeaways of the study before moving on to the Introduction section of Study 2. As mentioned in the previous comment, a comprehensive set of analyses were run. It would be helpful to include a *Discussion section* here that helps focus the readers’ attention on the main findings the authors care about.
The same applies to the end of Study 2: It would be good to add a brief interim Discussion section summarizing that study’s main take-aways before moving on the General Discussion section.

Authors’ response:

We added *discussion section*s at the end of both Study 1 and Study 2, respectively, to provide brief interim discussions and thereby improve the clarity of the manuscript’s overall structure.

The relevant revisions have been highlighted in the manuscript. Thank you again for your valuable suggestion.

Comments to the author:

5.Lines 399-401: It may be good to share a little more how the article pages were designed for the scenario manipulation. The authors do state that the articles were inspired by Wang et al…[61]. However, it would be helpful to provide more context, and to mention what features the manipulation controls for (e.g., length of the texts, difficulty, emotional language, etc.). If these things were not controlled for, it may be good to discuss this as a limitation. (This could be added to the part where the authors discuss the possible external validity of the scenario manipulation (lines 521-522).)

Authors’ response:

Regarding the manipulation materials used in the scenario experiment, some relevant details were omitted in the original manuscript due to word limit constraints. In the revised manuscript, we have added a description of the development and selection process of the experimental materials in the PAC Manipulation and Pretest section (Lines 499–514). We also supplemented the manuscript with an explanation of how the manipulation controlled for factors such as textual content and length.

At the same time, in the *Limitations and Future Directions section* (Lines 821–833), we further noted the limitations of relying on brief text-based scenarios and provided corresponding directions for future research.

The relevant revisions have been highlighted in yellow in the manuscript. Thank you again for your valuable suggestion.

Comments to the author:

6. Line 489: The authors state that AAV (algorithm aversion) can be conceptualized “as a stable attitudinal trait”.
AAV might be generally stable, but one could imagine that it is not necessarily ‘fixed’. AAV could very well respond to experience. For instance, AAV might be higher as a result of particular work/life experiences.
Given this, could the AAV level have conceivably responded to the PAC manipulation? This could be ruled out by checking for a potential difference between the experimental and control groups. (If this hasn’t already been done.) If there is no difference in AAV between groups, this could be briefly stated. If there is a difference in AAV between groups, it would be good to report this difference, and address what this could mean about the relationship between PAC and AAV. (Note: Apologies if AAV for the two groups was in fact reported and this reviewer missed it!)

Authors’ response:

We sincerely thank the reviewer for these valuable suggestions. These comments are highly important for improving the rigor and internal logical consistency of the study. In response, we have carefully revised the manuscript. Our clarifications regarding the key issues are as follows:

**Clarification and correction regarding the measurement order of AAV.** First, we sincerely appreciate the reviewer’s careful observation regarding the potential concern caused by the measurement order of AAV. We would like to clarify that, in the actual experimental design, the measurement of AAV was administered before the presentation of the scenario manipulation materials. The statement in the original manuscript indicating that AAV was measured after the manipulation was a writing error, which we have corrected in the revised manuscript in the Experimental Procedure and Variable Measurement section (Lines 527–535).

Because the AAV data were collected prior to the experimental manipulation, participants had not yet been exposed to the experimental materials when completing the AAV measure. Therefore, the manipulation of PAC could not have influenced participants’ AAV.

**Balance test and statistical control for the baseline difference in AAV between the two groups.**As the reviewer correctly pointed out, although AAV was measured in advance, an independent-samples t-test showed that there was indeed a statistically significant difference in baseline AAV between the experimental group and the control group (t = -6.732, p < 0.001). This suggests an imbalance in sample distribution generated during the random assignment process.

To ensure the robustness of our conclusions, we took the following steps. First, in the Manipulation Test section (Lines 547–556), we added an explanation of the balance test for AAV and explicitly reported this baseline difference. Second, in the hypothesis testing, we strictly controlled for the main effect of AAV in the hierarchical regression models (see Table 7). By including both the main effect of AAV and its interaction term with PAC in Model 3 (M3), we effectively controlled for the variance associated with the baseline imbalance, thereby increasing confidence that the observed interaction effect (i.e., the moderating effect) is robust. The relevant revisions have been highlighted in yellow in the manuscript. Thank you again for your valuable suggestion.

Comments to the author:

Minor Comments:
1. It looks like in the submission information that accompanies the manuscript, the abstract was accidentally included twice.
2. Line 85 → “study contributes to…” what? It looks like there are some missing words.
3. Line 98 → “These” : The second "e" is in bold.
4. Line 107 → It would be good to include a citation for the definition of PAC (perceived algorithmic control).
5. Line 109 → Missing comma after “However, SVCB” (Note: SCVB is already clear at this point. Perhaps authors don’t need to repeat its definition here.)
6. Line 267 → What is meant by “inconsistent responses” here? It would be good to quickly clarify.
7. Lines 283-84, 287, 291, 294 → The Cronbach’s alpha is used to compare consistency of responses between the first and second wave, is that correct? It would be good to clarify this at least once, the first time the statistic is reported.
8. Line 301 → Potential mistake here? How about "was utilized to perform" rather than “unutilized perform”.
9. Line 298-303 → It’s a good habit to include citations for the tools used to perform the analyses.
10. Line 310 → Maybe quickly clarify the “alternative models.” E.g. alternative models, in which the variables are grouped into three or fewer factors.
11. Line 328 → It’s usually good form to report p-values that are greater than 0.001. (Not simply state <0.05 or <0.01). If it’s too cumbersome to do in the main manuscript text, these p-values could be included in the appendices.
12. Line 342 → Hypothesis testing section: This may be due to this reviewer’s lack of familiarity with SPSS’ approach to hierarchical regression analyses, but it might be helpful to spend a bit more introducing the analyses that were run. For instance, what is the difference between the 4 models shown in Table 3? Some more context may help readers understand the take-aways better.
13. Line 356 → Do we need to know how the PAC and WO variables were standardized?
14. Lines 436-38 → Hypothesis testing section: Same as the comment above. It would be helpful to understand the difference between the 2 models shown in Table 7. Some more context would clarify the take-aways. (Even if the table shows the difference, a quick explanation in the text would be helpful.)
15. Line 442/Figure 3 → A bar graph may not be the best way to visualize the difference between the experimental and control group WO levels. E.g. A violin plot, box plot or other plots that help show the spread of the data typically do a better job of illustrating differences between groups.

Authors’ response:

We sincerely thank the reviewer for the detailed and constructive comments on our manuscript. We carefully reviewed the manuscript and revised the relevant issues point by point. Our responses to the Minor Comments are as follows:

1.Regarding the duplicated abstract submission. We have deleted the duplicated abstract content.

2.Regarding the incomplete expression in Line 85 (“study contributes to ……” ). We have completed the sentence to ensure that its meaning is complete and clearly expressed.

3.Regarding the formatting issue in the word “These” in Line 98. We have corrected the bold formatting error in this word.

4.Regarding the missing reference for the definition of PAC in Line 107. Thank you for this suggestion. We have added an appropriate reference to support the definition of PAC.

5.Regarding the missing comma after “However, SVCB” in Line 109. We have inserted the missing comma and polished the sentence accordingly.

6.Regarding the unclear meaning of “inconsistent responses” in Line 267. Because the matching of the two-wave data was primarily based on the last four digits of the participants’ phone numbers, the term “inconsistent responses” here actually referred to questionnaires with mismatched identification codes. We have revised this expression accordingly.

7.Regarding the explanation of Cronbach’s α in Lines 283–284, 287, 291, and 294. We have added a clarification when Cronbach’s α is first reported, explicitly stating that it is used in this study to assess the internal consistency reliability of the scales.

8.Regarding the inappropriate wording “unutilized perform” in Line 301. We have revised this expression to a more appropriate wording.

9.Regarding the need to add references for the analytical tools described in Lines 298–303. We have added the relevant references when describing the analytical tools.

10.Regarding the need to briefly explain “alternative models” in Line 310. We have added a clarification that the “alternative models” refer to competing models in which the core variables were combined into three-factor, two-factor, or single-factor structures, so that readers can better understand the analytical process.

11.Regarding the suggestion to report exact p values in Line 328. Because presenting all exact p values one by one in the main text would reduce the conciseness of the manuscript, we reported them in the supplementary materials instead. The exact p values have been uploaded in S2 Appendix, S3 Appendix, and S5 Appendix for readers’ reference.

12.Regarding the need for more background explanation in the hypothesis testing sectionin Line 342. We have added a brief explanation of the hierarchical regression models in the main text, clarifying the differences among the models in the table to help readers better understand the analytical logic and conclusions.

13.Regarding whether PAC and WO were standardized in Line 356. We have clarified this part of the manuscript by explicitly explaining how the relevant variables were treated, in order to avoid ambiguity.

14.Regarding the need to explain the model differences in Table 7 mentioned in Lines 436–438. We have added an explanation in the main text to clarify the differences among the models in Table 7 and their corresponding analytical purposes, so as to enhance the clarity of the results interpretation.

15.Regarding the suggestion in Line 442 / Figure 3 that a boxplot or violin plot would be more appropriate. We agree with this suggestion and have replaced the original bar chart with a boxplot to better present the distributional differences in WO levels between the experimental group and the control group.

Once again, we sincerely thank the reviewer for the careful review and positive evaluation of our manuscript. We greatly value these comments and believe that these revisions have substantially improved the clarity, rigor, and overall quality of the manuscript.

**Reviewer #3:** The manuscript is technically sound and the data support the conclusions of the paper. the data was carried out rigorously well. The authors provided adequate data that make the manuscript to present a detailed ideas and information. The manuscript is presented in standard English language.

**Authors’ response:**

We sincerely appreciate Reviewer #3’s thoughtful evaluation. We are gratified that the reviewer found the manuscript to be technically sound, the analyses to be rigorous, and the conclusions to be well supported by the data. We also thank the reviewer for acknowledging the clarity of our writing. Your positive comments are a great encouragement to our team. We have addressed the comments raised by the other reviewers and hope that the revised version meets the journal's standards.
