# Supplementary material for: How to break free from the "tool" dilemma? A study on the impact of perceived algorithmic control on sustainable value co-creation behavior
Source: PLoS One. 2026 Sep 8;21(9):e0357097. doi: 10.1371/journal.pone.0357097 (PMC13552789; doi:10.1371/journal.pone.0357097)
Supplement: Supplementary file 5 [file pone.0357097.s009.docx]

**Response to reviewers**

**Reviewer #1:** The revised manuscript has improved substantially compared to the previous version. The authors have responded carefully to most of the reviewers’ comments, particularly in clarifying conceptual distinctions (e.g., workplace objectification vs. organizational dehumanization), refining causal language, and strengthening methodological transparency. The addition of limitations, interim discussions, and improved reporting of analyses has enhanced the rigor and readability of the paper.

Overall, the manuscript is now much clearer, more balanced, and closer to publication quality. That said, I still have several suggestions that could further strengthen the theoretical contribution and positioning of the study.
mini

**Comments to the author:**

1. Theoretical Contribution Still Needs Stronger Positioning
   While the authors have addressed prior concerns, the theoretical contribution remains somewhat underdeveloped or conservatively framed. The manuscript currently positions its contribution mainly as: applying objectification theory to gig work, extending trait activation theory into algorithmic contexts. However, this positioning still reads more as application/extension rather than theory advancement.
   I recommend the authors more explicitly articulate: What new mechanism is revealed beyond existing literature?
   How does algorithmic control reshape traditional psychological processes?
   What is uniquely theoretical (not just empirical) about combining PAC + WO + AAV?

**Authors’ response:**

We sincerely thank the reviewer for the constructive suggestions regarding the theoretical contribution. Based on your suggestions, we have revised the marginal contribution section in the Introduction (lines 133–134 and 139–142 in the revised manuscript) and the Theoretical Implications section to more clearly articulate the theoretical contribution of this paper relative to existing research on perceived algorithmic control. We have clarified that the core contribution of this paper is to reveal how perceived algorithmic control transforms workers’ evaluations of algorithmic systems into their instrumentalized self-understanding and self-construction in the platform labor process.

First, regarding the new mechanism revealed in this paper, we have added and strengthened the following statements in the Theoretical Implications section:“Existing research on algorithmic management has often explained workers’ negative reactions from the perspective of how they evaluate algorithmic systems or platform management practices, such as whether algorithms are perceived as fair, transparent, autonomy-restricting, or stressful. These studies mainly focus on workers’ evaluations of external algorithmic systems or organizational management practices, whereas this study shifts attention to how platform workers understand their own position in the labor process under algorithmic management.”

We have also added the following clarification:“In other words, algorithmic control not only affects how workers evaluate algorithms, but may also change how they understand themselves, leading them to view themselves as usable, controllable, and replaceable functional units within the platform system. Thus, WO captures a psychological process through which algorithmic control is internalized into workers’ self-understanding.”

Through the above revisions, we have made clear that the psychological mechanism revealed in this paper is that perceived algorithmic control may further change platform workers’ self-perception in their own labor process, leading them to experience workplace objectification as being treated instrumentally.

Second, regarding how algorithmic control affects traditional psychological processes, we have added the following statement:“Therefore, while this study examines WO as a mediating variable, more importantly, it reveals a psychological mechanism of algorithmic control that differs from existing explanations. PAC may weaken SVCB by changing workers’ understanding of their own position in the labor process, leading them to experience themselves as being treated as instruments.” The theoretical contribution of this paper is to advance the explanation of the negative effects of algorithmic control from how platform workers evaluate algorithms or platform management to how platform workers re-understand their own labor process under algorithmic control.

Finally, regarding the theoretical integration logic among PAC, WO, and AAV, we have added the following statement in the Theoretical Implications section:“Technologized and depersonalized algorithmic control, as an important situational cue, can activate individuals’ pre-existing attitudes toward algorithms and influence whether external algorithmic control is transformed into an instrumentalized understanding of the self. In this process, PAC represents the technologized situational cue in platform algorithmic management, WO reflects workers’ instrumentalized self-understanding under this cue, and AAV explains why this self-oriented psychological response differs across workers.”

Through the above revisions, we have more explicitly responded to the reviewer’s three concerns: First, the new mechanism revealed in this paper is that perceived algorithmic control changes workers’ understanding of their own labor position through workplace objectification; second, perceived algorithmic control not only affects how workers evaluate algorithms, but may also change how they view themselves; third, the theoretical integration of PAC, WO, and AAV explains how perceived algorithmic control is transformed into instrumentalized self-understanding and among which workers such transformation is more likely to occur.

The relevant changes have been made in the marginal contribution section of the Introduction and the Theoretical Implications section (lines 660–760) and have been highlighted in yellow in the manuscript. Thank you for your suggestion.

**Comments to the author:**

The authors have made a good effort to clarify the distinction between workplace objectification (WO) and organizational dehumanization, and have toned down overly strong claims. However, the distinction is still largely conceptual and narrative. There is no empirical differentiation between the constructs.

**Authors’ response:**

We thank the reviewer for raising this important concern. We agree with the reviewer’s assessment that, although this study theoretically discussed the distinction between workplace objectification (WO) and organizational dehumanization, because we did not measure both constructs simultaneously within the same research design, we are unable to empirically test their empirical boundary in the algorithmic management context through discriminant validity tests, confirmatory factor analysis, incremental validity tests, or competing model comparisons. Based on your suggestion, we have revised the Theoretical Implications section and the Limitations and Future Directions section, and re‑examined the relevant discussion of WO throughout the manuscript to avoid conflating WO with organizational dehumanization.

First, in the Theoretical Implications section, we have clarified the theoretical boundary between WO and organizational dehumanization. Specifically, we have added and revised the following statements:“Organizational dehumanization places greater emphasis on employees’ external evaluation of the organization or management system, that is, employees feel that the organization denies their human attributes, such as emotion, warmth, individuality, and agency, and treats them as objects lacking full human qualities. In contrast, WO places greater emphasis on individuals’ instrumentalized self‑experience in the labor process, that is, workers feel that they are merely tools, resources, components, or functional units for achieving organizational or platform goals.”

And we have added a clarification of the key distinction between the two:“Therefore, the key distinction between the two concepts is that organizational dehumanization focuses more on how workers evaluate whether the organization or management system denies their humanity, whereas WO focuses more on how workers understand their own position in the labor system, especially whether they experience themselves as usable, controllable, and replaceable instrumental beings.”

We have made clear that organizational dehumanization focuses more on how workers evaluate whether the organization or management system denies their humanity, whereas WO focuses more on how workers understand their own position in the labor system, especially whether they experience themselves as usable, controllable, and replaceable instrumental beings.

Second, in the Theoretical Implications section, we have further explained why this study adopts WO as the theoretical entry point, and we have avoided stating that this study has empirically proven that WO is superior to organizational dehumanization. We have added and revised the following statements:“Therefore, this study adopts WO as the theoretical lens to more accurately explain how algorithmic management leads platform workers to develop the subjective experience that I am merely an instrumental resource within the system.”

What this study focuses on is how perceived algorithmic leads platform workers to gradually understand themselves as executive nodes, functional components, or instrumental extensions within the algorithmic system.

Finally, we agree with the reviewer’s comment that “there is no empirical difference between these two constructs.” Because this study did not measure WO and organizational dehumanization simultaneously within the same research design, we are unable to directly test their empirical boundary in the algorithmic management context through discriminant validity tests, incremental validity tests, or competing model comparisons. Therefore, we have revised the relevant statements in the manuscript to avoid presenting the distinction between WO and organizational dehumanization as a conclusion that has been empirically supported by this study, and instead present it only as the conceptual basis for the theoretical choice in this study.

At the same time, we have added a discussion of this limitation in the Limitations and Future Directions section, stating that this study did not empirically distinguish between WO and organizational dehumanization within the same research design:“This study did not measure WO and organizational dehumanization simultaneously within the same research design, but only distinguished them theoretically. Therefore, it cannot directly test their empirical boundary in the context of algorithmic management.”

And we have noted that future research could include both WO and organizational dehumanization and use discriminant validity tests, incremental validity tests, competing model comparisons, and analyses of differentiated mechanisms to further clarify the relationship and distinction between the two constructs in the context of platform algorithmic management:“Future research could include both WO and organizational dehumanization and use discriminant validity tests, incremental validity tests, competing model comparisons, and analyses of differentiated mechanisms to further clarify the relationship and distinction between the two constructs in the context of platform algorithmic management.”

The relevant changes have been made in the Theoretical Implications section (lines 685–707) and the Limitations and Future Directions section (lines 892–904) and have been highlighted in yellow in the manuscript. Thank you for your suggestion.

**Comments to the author:**

3. Missing Integration of Relevant Literature (Important). One important issue is that some relevant and recent literature (previously suggested) has not been incorporated into the manuscript. I recommend some related articles maybe you can cited in your research, such as: Are resources always beneficial? The three-way interaction between slashies’ role stress, self-efficacy and job autonomy. Asia Pacific Journal of Management, 2025, 1-31.; When employees meet digital-intelligence transformation: Unveiling the role of employee intentions. International Journal of Information Management, 2025, 84: 102912.; Exploring the impact of forced teleworking on counterproductive work behavior: the role of event strength and work-family conflict. Internet Research, 2026, 36.1: 53-77.

**Authors’ response:**

We thank the reviewer for this important suggestion. Based on the reviewer's recommendation, we have added and integrated the relevant literature in the revised manuscript.

First, in the Introduction section, we cite Wu et al. (2025) to illustrate that when digital and intelligent technologies intervene in the work process, individuals' attitudes, intentions, and perceived control affect their behavioral responses to technological change (lines 55–57); in the hypothesis development section on the effect of perceived algorithmic control on sustained value co-creation behavior, we cite Duan et al. (2025) to support the view that autonomy is an important work resource in the context of flexible employment (lines 179–182); in the hypothesis development section on the moderating role of algorithm aversion, we cite Liang et al. (2026) to illustrate that salient work events may trigger psychological stress and affect subsequent behavioral responses (lines 266–271). Second, we have also added a citation to Lin et al. (2025), a recent study on perceived algorithmic control and gig workers' work engagement, to further connect the latest literature on algorithmic control and platform workers' psychological and behavioral consequences (lines 57–60).

The relevant changes have been highlighted in yellow in the manuscript. Thank you for your suggestion.

**Reviewer #2:** We commend the authors on their thorough revisions to the manuscript. The issues brought up in the previous round of reviews have been carefully addressed.

**Major Comments:**

Comments to the author:

There are a few minor corrections recommended:

- Line 65 → Replacing "workers being experienced as..." with "workers being treated as..." might be more appropriate for this sentence.

- Line 90 → Typo: the "pace" of work should be the "place" of work

- Line 138 → The "e" in "These" is in bold

- Line 422 → There seems to be a missing word: “and entered *in* the regression model”

- Lines 673-676 → The authors state that "At the same time, this study also theoretically demonstrates that, in the context of platform algorithmic management, WO is better able to capture workers lived perceptions of instrumentality and replaceability than the broader concept of organizational dehumanization".
Given that dehumanization and WO are not pitted against each other in the design of the studies, it may be good to avoid stating this too strongly here. This reviewer recognizes that this was addressed in the limitations section later on in the paper, but it may also be good to avoid this here as well.

---

Once again, we applaud the authors on their thorough revisions!

Authors’ response:

Thank you very much for your careful review of our manuscript and for your positive recognition of our previous revisions. We greatly appreciate your constructive comments, which have helped us further improve the clarity and rigor of our paper.

In response to your comments, we have made the following revisions to the manuscript:

First, the sentence on line 65 has been revised from "workers being experienced as" to "workers being treated as" to improve clarity.

Second, the spelling error on line 90 has been corrected, changing "pace" to "place of work."

Third, on line 138, the extra bolded letter "e" in the word "These" has been removed.

Fourth, on line 422, the missing word "in" has been added, and the phrase now reads "and entered in the regression model."

Finally, regarding the comparison between workplace objectification (WO) and organizational dehumanization on lines 673–676. We agree with the reviewer's assessment that, because the research design of this study did not directly compare WO and organizational dehumanization, it is inappropriate to make a strong claim that WO is superior to organizational dehumanization.

Therefore, we have revised the relevant statements in the Theoretical Implications section. The original statement:"At the same time, this study also theoretically demonstrates that, in the context of platform algorithmic management, WO is better able to capture workers' lived perceptions of instrumentality and replaceability than the broader concept of organizational dehumanization."has been revised to:"Therefore, this study adopts WO as the theoretical lens to more accurately explain how algorithmic management leads platform workers to develop the subjective experience that I am merely an instrumental resource within the system."

In addition, we have rephrased the conceptual discussion of WO and organizational dehumanization in the Theoretical Implications section to more clearly articulate the theoretical focus of each construct: "Organizational dehumanization places greater emphasis on employees' external evaluation of the organization or management system, that is, employees feel that the organization denies their human attributes, such as emotion, warmth, individuality, and agency, and treats them as objects lacking full human qualities. In contrast, WO places greater emphasis on individuals' instrumentalized self-experience in the labor process, that is, workers feel that they are merely tools, resources, components, or functional units for achieving organizational or platform goals." This further clarifies that organizational dehumanization focuses more on employees' external evaluation of whether the organization or management system denies their human attributes, whereas WO focuses more on workers' instrumentalized self-perception in the labor relationship.

At the same time, we have further acknowledged this issue in the Limitations and Future Directions section, as follows: "Fourth, this study did not measure WO and organizational dehumanization simultaneously within the same research design, but only distinguished them theoretically. Therefore, it cannot directly test their empirical boundary in the context of algorithmic management. Future research could include both WO and organizational dehumanization and use discriminant validity tests, incremental validity tests, competing model comparisons, and analyses of differentiated mechanisms to further clarify the relationship and distinction between the two constructs in the context of platform algorithmic management."

Through the above revisions, we have removed the overly strong comparative claim in the Theoretical Implications section and further clarified that this study adopts WO as the theoretical entry point based on the specific focus of our research question. The relevant changes have been made in the Theoretical Implications section and the Limitations and Future Directions section and have been highlighted in yellow in the manuscript. We thank the reviewer again for the valuable suggestions.

The relevant changes have been highlighted in yellow in the manuscript. Once again, thank you for your affirmation of our revision work and for your valuable suggestions.
