# Supplementary material for: How to break free from the "tool" dilemma? A study on the impact of perceived algorithmic control on sustainable value co-creation behavior
Source: PLoS One. 2026 Sep 8;21(9):e0357097. doi: 10.1371/journal.pone.0357097 (PMC13552789; doi:10.1371/journal.pone.0357097)
Supplement: Supplementary file 6 [file pone.0357097.s010.docx]

**Response to reviewers**

**Reviewer #1:** Having carefully reviewed this revised manuscript and the authors’ responses to the previous reviewers, I commend the authors for their thorough and thoughtful revisions. They have substantially improved conceptual clarity (especially regarding workplace objectification vs. organizational dehumanization), strengthened methodological transparency, and toned down overclaims. The two-study design (multi-wave survey + scenario experiment) provides convergent evidence for the proposed moderated mediation model. However, to further strengthen the manuscript prior to final acceptance, I offer the following comments for the authors to address in a final revision.

mini

**Comments to the author:**

1.While Study 2 successfully manipulates PAC and tests its effect on WO (H2a) and the moderation by AAV (H3a), the full mediating pathway (PAC → WO → SVCB) and the moderated mediation (H3b) are only tested in Study 1’s correlational design. The authors acknowledge this limitation but do not attempt any supplementary causal mediation analysis (e.g., sensitivity analysis for potential unmeasured confounding) nor do they explicitly state that the full mediation model remains correlational.

**Authors’ response:**

We would like to once again express our sincere gratitude for the reviewer's thorough review and constructive comments on our manuscript. Regarding the first concern—that the full mediation model is based on correlational data and should be explicitly stated as such—we have made targeted additions in the General Discussion section (see the revised manuscript, with the added text highlighted in yellow):

"It should be noted, however, that the full mediation model (PAC → WO → SVCB) and the moderated mediation model (H3b) were tested using correlational data from Study 1. Although Study 2 provided experimental evidence for the effect of PAC on WO and the moderating role of AAV, the complete mediating pathway and the moderated mediation effect remain correlational in nature and should not be interpreted as establishing strict causal ordering."

Here, we would like to further clarify the methodological logic of our two-study design. The purpose of our two-study design is not to attempt to establish all causal relationships with a single study; rather, through a progressive logic of "correlational → experimental," we leverage the distinct strengths of each study: Study 1 tests the full model in real platform work contexts, providing external validity—demonstrating that the theoretical model holds in real-world settings; Study 2, through experimental manipulation, provides internal validity for the PAC → WO relationship—establishing the causal nature of this key link. The full model indeed remains correlational in nature, but Study 2 provides causal evidence for the first half of the mediating pathway (PAC → WO), which strengthens our confidence in the overall model and clearly points to the direction for future research to further examine the complete causal chain.

Once again, we thank the reviewer for the rigorous scrutiny; these comments have greatly improved the accuracy and academic rigor of our methodological discussion.

**Comments to the author:**

2. AAV is measured at Time 1 in Study 1 and before manipulation in Study 2, which is appropriate. However, the authors do not test whether AAV itself might be influenced by prior PAC experiences (i.e., reverse causality or reciprocal dynamics). At minimum, discuss this as a limitation: “AAV was treated as a stable individual difference, but longitudinal designs are needed to rule out that prior PAC experiences may shape AAV over time.”

**Authors’ response:**

Regarding the discussion of the limitation that AAV was treated as a stable individual difference, we agree with the reviewer's viewpoint. Although measuring AAV at Time 1 in Study 1 and before the manipulation in Study 2 is methodologically reasonable in terms of temporal precedence, we acknowledge that this study indeed did not examine whether AAV itself might be influenced by prior PAC experiences. As the reviewer rightly pointed out, while AAV was treated as a stable individual difference, prior PAC experiences may potentially shape individuals' AAV levels over time, thereby giving rise to reverse causality or reciprocal dynamics.

In accordance with the reviewer's suggestion, we have supplemented the relevant content in the limitations section of the revised manuscript (lines 895-900), with the added text highlighted in yellow. The supplementary content reads as follows:

"Furthermore, although measuring AAV at Time 1 in Study 1 and before the manipulation in Study 2 is methodologically reasonable in terms of temporal precedence, this study did not examine whether AAV itself might be influenced by prior PAC experiences. Future research should adopt longitudinal designs to rule out the possibility that prior PAC experiences may shape AAV over time, thereby further clarifying the causal direction between the two variables."

This addition makes the limitations discussion of this study more comprehensive and rigorous, and also points to a promising direction for future research to further explore.

Once again, we thank the reviewer for the valuable suggestions, which have significantly enhanced the academic rigor of our manuscript.

**Reviewer #2:** Well done to the authors for addressing the comments in previous rounds of reviews. There are a few minor comments left:

**Major Comments:**

Comments to the author:

Lines 90-93: It's not clear what theory, evidence (or past research) the authors are drawing from to state that: "Therefore, compared with the broader concept of organizational dehumanization, WO is better able to capture platform workers’ perceptions of instrumentality and replaceability under algorithmic control." As in previous rounds of revisions, we suggest authors be careful about explicitly pitting organizational dehumanization against WO; unless, of course, there is strong backing from previous work in this case. Lines 264 to 276: It seems like the same argument is being made and discussed twice in a row: "AAV is likely to be activated by two key situational cues " (First on line 264 and next on line 270) But the two cues that are listed each time are a bit different, though they both include 'unpredictable algorithmic cues" as one of them. The discussion here could be tightened a little to avoid the repetition. Lines 676-690 and lines 705-724: These two paragraphs, that begin with "Third, this study further clarifies", seem to be repeating the same content. It looks like paragraph that discussed point "Two" may have been taken out by mistake and replaced with a repeated point "Three"?

Authors’ response:

We sincerely thank the reviewer for the careful review and valuable comments on our manuscript. Regarding the concern about the lack of clear theoretical or empirical basis for the statement in lines 90-93, we have carefully reflected on this issue and made corresponding revisions. Our detailed responses are as follows:

On the issue of the statement that "WO is better able to capture platform workers' perceptions" (lines 90-93):

We greatly appreciate the reviewer's prudent reminder. We fully accept the suggestion. We have revised the sentence as follows:

Original sentence: "Therefore, compared with the broader concept of organizational dehumanization, WO is better able to capture platform workers' perceptions of instrumentality and replaceability under algorithmic control."

Revised sentence: "Therefore, focusing on the research context of this study, WO is better able to capture platform workers' perceptions of instrumentality and replaceability under algorithmic control."

The revised wording shifts the focus of the argument from "comparison between constructs" to "fit with the research context," thereby preserving the logical coherence of the original argument while effectively avoiding any comparative or oppositional interpretation that might arise from explicitly mentioning organizational dehumanization.

On the issue of redundant discussion in lines 264-276:

In accordance with the reviewer's suggestion, we have thoroughly reorganized and rewritten this paragraph (now located near line 267), removing redundant statements. The specific revisions have been highlighted in yellow at line 267 of the revised manuscript for the reviewer's reference. The new formulation avoids the mechanical, itemized listing of the previous version, making the argumentation more concise and focused.

On the issue of content repetition between lines 676-690 and lines 705-724:

The reviewer correctly pointed out that lines 676-690 and lines 705-724 indeed repeated the same "Third" point. Upon careful verification of the revision process, we confirmed that this was due to an operational error during the adjustment of paragraph structure in the previous round of revision, which resulted in the unintended deletion of the paragraph discussing the "Second" point, while the redundant "Third" point was retained. Accordingly, we have thoroughly corrected this section as follows: (1) The redundant paragraph at lines 705-724 has been deleted. (2) The content at lines 676-690 has been rewritten to serve as a complete discussion of the "Second" point, with the substantive content regarding the study's theoretical contributions that was originally omitted now supplemented.

We sincerely apologize for the inconvenience caused by this editorial oversight during the reviewer's evaluation, and we are grateful for the reviewer's rigorous scrutiny. The theoretical contributions of this section are now presented in a clear and logically structured manner.

Once again, we thank the reviewer for the valuable suggestions, which have significantly enhanced the quality of our manuscript. We hope that our revisions meet the reviewer's expectations.
