# Supplementary material for: How to break free from the "tool" dilemma? A study on the impact of perceived algorithmic control on sustainable value co-creation behavior
Source: PLoS One. 2026 Sep 8;21(9):e0357097. doi: 10.1371/journal.pone.0357097 (PMC13552789; doi:10.1371/journal.pone.0357097)
Supplement: Supplementary file 7 [file pone.0357097.s001.doc]

**S1 Appendix. Lists of measures**

****Item statements of the main variables****

| **Constructs** | **Measurements** | |
| --- | --- | --- |
| **Perceived Algorithmic Control (PAC)** | PAC1 | The algorithm intelligently allocates work tasks to me. |
|  | PAC2 | The algorithm provides standardized instructions for my work based on platform criteria. |
|  | PAC3 | The algorithm delivers substantial informational support for task completion. |
|  | PAC4 | The algorithm provides real-time dynamic feedback on work performance. |
|  | PAC5 | The algorithm continuously tracks and geolocates my position. |
|  | PAC6 | The algorithm monitors my work progress in real time. |
|  | PAC7 | The algorithm assesses my work attitude through real-time surveillance. |
|  | PAC8 | The algorithm automatically evaluates the quality of my work output. |
|  | PAC9 | The algorithm ranks and classifies my performance within the platform. |
|  | PAC10 | The algorithm incentivizes my efforts with time-bound monetary rewards. |
|  | PAC11 | The algorithm imposes financial penalties when my work fails to meet platform requirements. |
| **Sustainable Value Co-creation Behaviour (SVCB)** | SVCB1 | I will keep offering feedback to the platform for service improvement. |
|  | SVCB2 | I will persist in fulfilling my duties and obligations as an employee. |
|  | SVCB3 | I will keep recommending the platform to friends and family. |
|  | SVCB4 | I will remain tolerant of passengers'/customers' shortcomings. |
|  | SVCB5 | I will continue to overlook the platform's minor occasional mistakes. |
| **Workplace Objectification（WO）** | WO1 | The platform conveys an impression that my job function is replaceable by automated systems. |
|  | WO2 | The platform prioritizes instrumental utility (what I can contribute) over reciprocal support (what it can offer me). |
|  | WO3 | From the platform's perspective, my subjective experiences (e.g., emotions, opinions) are irrelevant as long as my physical labor capacity remains intact. |
|  | WO4 | Workplace evaluations suggest the platform perceives machine substitution as equally or more effective than my human labor. |
|  | WO5 | The platform reductively equates my skill set with mere physical endurance. |
|  | WO6 | Operational practices reflect the platform's implicit devaluation of worker health and safety protections. |
|  | WO7 | My contractual relationship with the platform is contingent solely on continued functional utility. |
|  | WO8 | Worker wellbeing (health/physical condition) is systematically treated as a secondary consideration. |
|  | WO9 | The employment contract effectively commodifies my labor, granting the platform discretionary control akin to product ownership. |
|  | WO10 | Engagement is strictly demand-driven - the platform initiates contact only during labor shortages. |
| **Algorithm Aversion（AAV）** | AAV1 | I feel uneasy as platform algorithms have a precise grasp of my preferences. |
|  | AAV2 | I am averse to platform algorithms collecting my personal privacy information. |
|  | AAV3 | I worry about the potential safety risks that platform algorithms may entail. |
