# Supplementary material for: How to break free from the "tool" dilemma? A study on the impact of perceived algorithmic control on sustainable value co-creation behavior
Source: PLoS One. 2026 Sep 8;21(9):e0357097. doi: 10.1371/journal.pone.0357097 (PMC13552789; doi:10.1371/journal.pone.0357097)
Supplement: Supplementary file 8 [file pone.0357097.s004.doc]

**S4 Appendix. Original experiment stimuli in Study 2**

**Perceived Algorithmic Control - Experimental Group**

In recent years, the food delivery market has grown rapidly, with Meituan’s peak order volume reaching 16 million, making it the world’s largest platform. Facing fierce competition, platforms focus on supply quality, delivery experience, and software performance. Meituan’s delivery team aims to improve efficiency for over 400,000 riders while enhancing user satisfaction, reducing costs, and promoting system automation and intelligence.

They have optimised machine learning, operations research, and simulation technologies, launching a “super brain” for instant delivery tailored to on-demand scenarios. The system adjusts order structure by optimising delivery fees and times, considering rider location, ongoing orders, rider ability, restaurant prep time, weather, road conditions, and future demand. This ensures timely, suitable rider allocation and predicts delays for dynamic scheduling.

After assignment, riders receive estimated prep times and optimal routes via voice interaction. Post-delivery, the system alerts riders about demand across districts for real-time capacity management. These advances have cut average delivery time from 41 to 28 minutes and reduced per-order delivery costs by over 20%.

**Perceived Algorithmic Control - Control Group**

China’s traditional culture, shaped over millennia by diverse values, thoughts, and social contexts, remains vibrant today. Advertising not only attracts attention and promotes products but also integrates cultural elements to enhance appeal.

For instance, at Hangzhou’s Beiguan Night Market, businesses use traditional symbols like lanterns and wine jars or unique ingredients to draw customers. “Wine flags,” an ancient Chinese advertising form, enrich this cultural heritage.

Creative advertising should prioritise expressing traditional culture’s essence, moving beyond mere visuals or media forms. As technology advances, ads should harness ethnic characteristics and cultural thinking, not just colours or sounds, to better engage audiences and broaden reach. Modern communication tools further strengthen the influence of traditional culture in contemporary society.
