# Supplementary material for: How to break free from the "tool" dilemma? A study on the impact of perceived algorithmic control on sustainable value co-creation behavior
Source: PLoS One. 2026 Sep 8;21(9):e0357097. doi: 10.1371/journal.pone.0357097 (PMC13552789; doi:10.1371/journal.pone.0357097)
Supplement: Supplementary file 9 [file pone.0357097.s006.doc]

**How to break free from the "tool" dilemma? A study on the impact of perceived algorithmic control on sustainable value co-creation behavior**

Xuan Liu^1^，Yuqing Wang^1*^ , Haowei Zheng^1^ and Yuci Chen^1^

^1^ School of Economics and Management, Nanjing Tech University, Nanjing 211816, China

**^*^Correspondence:**

Yuqing Wang

[yuqing@njtech.edu.cn](mailto:yuqing@njtech.edu.cn)

# **Abstract**

With the growing prevalence of algorithmic technologies, algorithmic management has become a dominant control mechanism in the gig economy, profoundly shaping platform workers’ psychological states and behaviors. Grounded in objectification theory and trait activation theory, this study examines how perceived algorithmic control (PAC) affects platform workers’ sustainable value co-creation behavior (SVCB), highlighting the mediating role of workplace objectification (WO) and the moderating role of algorithm aversion (AAV). Using a two-wave survey (n = 285) and a scenario-based experiment (PAC experimental vs. control group; n = 216), the study finds that:(1) PAC significantly negatively affects SVCB; (2) PAC increases WO, which in turn reduces SVCB; (3) AAV positively moderates the indirect effect of PAC on SVCB through WO. This study advances understanding of the psychological mechanisms linking PAC to SVCB in gig work settings. It provides actionable insights for enhancing algorithmic governance and fostering sustained engagement among platform workers.

**Keywords** **Perceived algorithmic control**, **Sustainable value co-creation behavior**, **Workplace objectification**, **Algorithm aversion**

# **1 Introduction**

With the rapid growth of mobile internet and the gig economy, online labor platforms (OLPs)—such as Uber in the US and Meituan in China—have flourished. These platforms use algorithmic technologies to match fragmented user demands with workers’ spare time, reshaping traditional labor structures[1]. This has created a new labor force—platform workers—who perform tasks such as food delivery or ride-hailing under algorithmic control [2], such as food delivery riders and ride-hailing drivers. The penetration of platform-based work has been expanding and now constitutes a significant component of global employment [3]. In China, the Ali Research Institute predicts that by 2036, over 400 million people will engage in gig economy activities[4]. A similar growth trend is also evident across Western economies, where labor participation via platforms continues to rise [5, 6].Unlike traditional employment relationships, platform workers typically maintain a mutually beneficial, transactional relationship with the platform [7]. The long-term sustainability of platform enterprises depends heavily on the continued engagement and value creation of these workers [8].

Sustainable value co-creation behavior (SVCB) refers to employees’ proactive, ongoing engagement in value co-creation with their organization [9]. Within the context of the gig economy, SVCB is manifested in platform workers' use of their skills, time, and resources to provide services to users, thereby enhancing service quality and brand value in collaboration with the platform [10]. For platforms, SVCB contributes to improved operational efficiency, enhanced user experience, and sustained competitive advantage [11, 12]. For platform workers themselves, SVCB fosters job satisfaction and occupational identity, while also increasing access to orders, customer ratings, and platform-based rewards, ultimately enhancing income stability [9]. Therefore, how to effectively motivate SVCB among platform workers has become a critical concern for platform enterprises.

Unlike traditional organizations that use technical regulations and supervision, OLPs rely on algorithmic control. Algorithmic control automates task allocation, performance monitoring, and incentive adjustments [13]. While enhancing efficiency, algorithmic control can undermine workers’ autonomy and intrinsic motivation [14, 15], reducing engagement and increasing psychological stress[16]. Since the OLPs’s long-term success depends on sustained worker contribution [10], it is crucial to understand how algorithmic control influences SVCB.

Objectification Theory posits that individuals may be perceived and treated as objects or tools within social contexts, rather than being recognized as autonomous, emotionally sentient beings[17]. Originally applied to explore sexual objectification [18, 19], the theory has expanded to the workplace, giving rise to Workplace Objectification (WO). In highly standardized and controlling environments, workers are viewed as interchangeable production components [20]. WO refers to the process of perceiving individuals as objects, primarily reflecting instrumentality and the denial of human attributes [20, 21]. Unlike organizational dehumanization, which denies human uniqueness, WO emphasizes traits like instrumentality, disposability, lack of autonomy, and violability [22]. In the gig economy, platform workers’ experiences of data surveillance, automated decision-making, and performance evaluation reinforce their objectified status, as platforms treat them as task-completing tools rather than collaborative partners [21]. Research shows that WO weakens organizational identification and cooperation, reducing proactive behaviors such as service improvement and process optimization [23]. Therefore, this study posits WO as a critical mediator between PAC and SVCB.

The Job Demand-Control Model suggests that the impact of the work environment on individuals stems from the dynamic interaction between job demands and job control [24]. In the gig economy, platform work environments typically create a "high demand—low control" situation, where platform workers' task pace and behavioral autonomy are constrained by algorithms [14, 15], intensifying PAC. In such contexts, individual traits influence psychological responses. Algorithm Aversion (AAV), a stable trait reflecting negative attitudes towards algorithm-driven decisions [25, 26], may be triggered by high-intensity algorithmic control, making individuals more sensitive to perceptions of control and objectification. Thus, AAV likely moderates the effect of algorithmic control on WO.

Based on objectification theory and trait activation theory, this study constructs a moderated mediation model to explore how PAC influences platform workers' SVCB, with WO as a mediator and AAV as a moderator. The theoretical model is shown in Fig 1. The study contributes by: (1) expanding research on PAC by examining its impact on SVCB in gig work; (2) applying objectification theory to algorithmic management, showing how workers’ treatment as tools diminishes proactive value creation; and (3) identifying AAV as a boundary condition, highlighting how individual predispositions moderate the negative effects of PAC on WO and SVCB. These results shed light on the psychological mechanisms underlying algorithmic control and deliver actionable guidance for enhancing algorithmic governance and maintaining worker participation on OLPs.

**INSERT FIG 1 HERE**

**Fig 1. Theoretical model**

**2 Theoretical foundations and research hypotheses**

**2.1 Perceived algorithmic control（PAC） and sustainable value co-creation behavior（SVCB****）**

Unlike traditional organizational or leadership control, algorithmic control has become increasingly dominant on OLPs. Thes**e** OLPs employ algorithmic systems to conduct virtual surveillance, continuously enhancing efficiency and performance, thereby establishing a full-cycle governance model [15]. Throughout this process, platform workers increasingly perceive themselves to be under tight algorithmic supervision, with limited autonomy and discretion, giving rise to the experience of **PAC [27]**. PAC refers to workers’ cognitive and emotional perceptions of algorithmic intervention, comprising three dimensions: normative guidance, tracking and evaluation, and behavioral constraint. Normative guidance denotes the delivery of complex task requirements; tracking and evaluation involve stringent monitoring and performance assessment; and behavioral constraint refers to the immediate enforcement of rewards and penalties. On most OLPs, algorithmic systems govern the entire labor process—from task allocation to performance appraisal—undermining workers’ autonomy and discretion. However, SVCB defined as workers’ voluntary and sustained collaboration with platforms to generate value [9] , relies heavily on intrinsic motivation and autonomy [28] .Therefore, this study posits that PAC may undermine workers’ engagement in SVCB.

This inhibiting effect is primarily driven by the following factors: First, algorithmic systems are efficiency-oriented and optimize task allocation through standardized processes [10]. In a high PAC environment, workers tend to perceive their relationship with the platform as transactional, focusing on performance rather than relational investment. As a result, they may withhold emotional and cognitive resources, aiming only to fulfil minimum task requirements [29]. When workers further perceive that the platform’s algorithmic control is primarily cost-driven and lacks long-term developmental support, their emotional attachment to the platform erodes [30]. This weakens their motivation for proactive innovation and deep collaboration, ultimately suppressing their engagement in SVCB.

Second, the algorithmic normative and tracking mechanisms restrict autonomy and compress the space for platform workers’ innovation. The detailed task requirements delivered by algorithms (e.g., delivery time constraints) combined with stringent monitoring measures (such as real-time location tracking and process recording) create a "high demands–low control" work environment [27]. Prior research has established autonomy as a critical driver of proactive behavior [31]. When autonomy is stripped away by algorithmic management, platform workers are confined to executing tasks according to fixed procedures, losing the ability to adjust their work methods [32], which in turn diminishes their sense of control over their work. Since SVCB requires workers’ continuous active engagement [33], under conditions of constrained autonomy, platform workers find it difficult to propose improvements or innovations and tend to perform tasks mechanically rather than actively optimizing services.

Furthermore, OLPs reinforce behavioral constraints on workers through immediate reward and penalty mechanisms [27]. To avoid sanctions, workers tend to priorities short-term, compliance-oriented cooperation [34]. However, SVCB depends on collaborative interaction between individuals and organizations [35, 36]. The unidirectional obedience imposed by algorithmic control deprives platform workers of emotional support and feedback channels, leading them to perform tasks mechanically [37, 38] and to no longer regard proactive value creation behaviors as part of their work responsibilities, thereby reducing ongoing value co-creation with the platform. Therefore, PAC undermines platform workers’ emotional investment, autonomy, and social support through task execution, normative guidance, and behavioral constraints, intensifying psychological strain and behavioral restrictions, and ultimately weakening their capacity and willingness to engage in SVCB. On this basis, the hypothesis below is developed:

**H1:** **PAC** negatively affects platform workers’ SVCB.

**2.2 The mediating role of workplace objectification (WO)**

WO refers to the process of perceiving and treating individuals as instruments in the workplace, whereby their autonomy and human essence are denied [20, 21]. Based on objectification theory, highly standardized and controlled work environments reduce workers to replaceable production factors [20], leading to a dual experience of instrumentalization and vulnerabilities for platform workers. Within the setting of OLPs, platform workers often experience a dual process of instrumentalization and vulnerabilities. Instrumentalization occurs when algorithms reduce workers’ value to performance indicators—such as order volume or punctuality—while neglecting their professional development or emotional needs. Vulnerabilities, meanwhile, manifests in excessive task allocation driven by algorithmic efficiency, disregarding workers’ physical well-being [23]. Within algorithm-dominated labor systems, the phenomenon of WO is particularly prevalent.

This study argues that PAC positively predicts WO among platform workers. OLPs use algorithmic norms and performance-tracking systems to encourage compliance with efficiency targets [27]. Rules such as "delays result in penalty points" or "negative reviews affect task assignment" constrain worker behavior within algorithmic parameters [15]. Real-time tracking and ranking systems further reduce workers’ individuality to quantifiable data [39]. As workers internalize these logics, they are compelled to comply in order to maintain work opportunities, increasingly perceiving themselves as mechanical extensions of the system—thus heightening their experience of WO.

The algorithmic control mechanism systematically overlooks platform workers’ health rights, fostering a perception of physical vulnerability [40]. In pursuit of efficiency, OLPs often assign tasks that exceed normal workloads or schedule dense, time-consuming routes. As a result, workers increasingly feel controlled by algorithms and treated as tools to advance platform interests. For instance, delivery riders frequently receive multiple cross-regional orders within tight timeframes. Despite physical fatigue, they must rush to meet system-imposed countdowns [41]. Prolonged exposure to such high-intensity conditions reinforces the belief that algorithmic rules ignore physical limits and that the platform disregards worker health. This deepened sense of control leads to the perception that health is subordinate to efficiency, heightening the experience of WO. Thus, PAC intensifies platform workers’ WO.

WO further impedes platform workers’ SVCB. SVCB entails proactive engagement and discretionary effort [42], yet objectified workers often withdraw effort and limit performance [43]. According to objectification theory, continual evaluation through an instrumental lens erodes workers’ sense of purpose, leading them to equate self-worth with measurable output [17]. Treated as manipulable tools, they lose autonomy and cannot meaningfully engage in service innovation or process optimization [44], often settling for minimum task fulfilment.

Moreover, emotional engagement is essential for sustained co-creation [45]. Workers reduced to "data points" by algorithmic systems experience reinforced manipulation and instrumentalization [46], hindering deep cognitive or emotional connection with their work. WO also leads to emotional alienation and burnout [43], diminishing willingness to contribute beyond basic duties. Thus, platform workers in objectifying environments are less inclined to engage in emotionally driven SVCB, further inhibiting proactive value co-creation.

In conclusion, PAC leads to WO via the instrumentalization and vulnerability of workers, thereby inhibiting SVCB. This research advances the subsequent hypotheses:

**H2a**: **PAC** positively influences platform workers' **WO**.

**H2b**: **WO** negatively influences platform workers' **SVCB**.

**H2c**: **WO** mediates the relationship between **PAC** and **SVCB**.

**2.3 The moderating role of algorithm aversion (AAV)**

According to the Job Demand–Control Model, an individual's psychological and behavioral reactions stem from the interaction of job demands and their perceived control [24]. Within OLPs, algorithms impose high work demands through real-time tracking and strict time assessments, while restricting autonomy via rigid task allocation systems and limited rejection options [27]. This results in platform workers increasingly perceiving themselves as tools of the system, leading to heightened PAC and, consequently, greater WO.

Yet, individuals do not uniformly respond to algorithmic control. As noted by Y Zou, H Zhang, J Peng, Q Nie and Z Wang [47], psychological traits influence how people perceive and react to identical environments. One such trait is AAV—a dispositional resistance to algorithmic decision-making, marked by cognitive distrust, emotional discomfort, and behavioral rejection of algorithmic authority [25, 48]. Trait Activation Theory suggests that such latent traits are triggered by relevant contextual cues [49]. In the gig economy’s "high-demand, low-control" environments, AAV is likely to be activated by two key situational cues.

The first involves rigid rule-based cues—such as non-negotiable review systems and mandatory task assignments—signaling a lack of human discretion and autonomy [50]. The second entails unpredictable cues, such as sudden pricing changes or opaque algorithmic decisions, which introduce uncertainty and amplify AAV's defensive response. Together, these cues reinforce the belief that "algorithms equal control," especially among workers predisposed to algorithmic distrust.

When such cues are encountered, individuals with high AAV are more likely to experience intensified WO, both cognitively and emotionally. Cognitively, they interpret algorithmic instructions as reflecting disregard for human needs [51]. For instance, unrelenting deadlines are viewed not just as work stressors but as symbolic denials of their physical limits. Emotionally, these cues elicit stronger perceptions of loss of control and negative affective responses [52]. In line with Trait Activation Theory, the closer the match between trait and environment, the more intense the trait expression [49]. Therefore, under the same level of PAC, workers with high AAV experience significantly stronger WO than those with low AAV.

Moreover, high AAV individuals often possess entrenched schemas linking algorithmic management with neglect of worker wellbeing. When OLPs push dense delivery schedules without regulating rest or accounting for fatigue [21], these individuals are especially likely to interpret such practices as objectifying. They see themselves as expendable, evaluated solely by metrics like task completion or customer ratings, and deprived of their rights to health, agency, and recognition [23]. This reinforces the internalization of their role as a disposable tool, further deepening their experience of WO.

Based on these observations, this study proposes the following hypothesis:

**H3a**: AAV positively moderates the relationship between PAC and platform workers' WO.

In conclusion, the findings of this study provide robust evidence for the proposed moderated mediation model. We demonstrate that PAC adversely affects platform workers’ SVCB, with WO acting as a significant mediator in this association. Crucially, this mediated pathway is contingent upon a worker’s AAV. Specifically, for those with a higher AAV, the negative relationship between PAC and WO is notably stronger, thereby amplifying the negative indirect effect of PAC on SVCB via WO.

**H3b**: AAV positively moderates the indirect effect of PAC on platform workers' SVCB through WO. In other words, the stronger the AAV, the stronger the negative impact of PAC on SVCB through WO.

# **3 Study 1: Two-wave survey study**

**3.1 Sample and procedure**

This study received ethical approval from the Ethics Committee of Nanjing University of Technology (Approval No. NTJTECH-1-21). The recruitment period for this study is from November 1, 2024, through February 28, 2025. Data were collected via Credamo and Wenjuanxing, two widely used Chinese online platforms comparable to Prolific and MTurk. These platforms offer access to diverse participant pools and support flexible questionnaire design, thereby enhancing data quality. Prior to participation, respondents were presented with an electronic informed consent form outlining the study’s aims, procedures, and the right to withdraw at any time. Only those who provided consent were permitted to proceed. All responses were collected anonymously to ensure confidentiality.

A screening question was included in a pilot test: "Do you work on platforms such as Didi, T3, Caocao, Meituan, or Eleme?" Participants who responded "No" were automatically screened out. To mitigate common method bias, a two-wave research design was adopted. The two waves of data collection were conducted one month apart. In the first wave, data were collected on PAC, AAV and demographic variables. To reduce respondent fatigue and improve data quality, the following steps were taken: (1) Surveys were administered through Credamo and Wenjuanxing, enabling respondents to pause and resume at their convenience; (2) During data cleaning, responses with extremely short completion times, patterned answering (e.g., selecting the same option repeatedly), or failed attention checks (e.g., "Please select option 3") were excluded. Each valid respondent received a compensation of RMB 5 (approx. USD 0.71).

Of the 350 surveys distributed, 323 valid responses were obtained in the first wave (response rate = 92.29%). A month later, the same participants completed a follow-up questionnaire assessing WO and SVCB. After excluding inattentive or inconsistent responses, 285 valid questionnaires were retained (effective response rate = 88.24%).

Among the final sample, 177 participants (62.1%) were male and 108 (37.9%) were female. Most respondents (87.3%) were aged between 21 and 40. In terms of work experience, 98 participants (34.4%) had less than three years. Regarding education, 121 (42.4%) had a high school diploma or below, 114 (40.0%) held a college degree, and 50 (17.5%) held a bachelor’s degree. A total of 212 respondents (74.4%) were full-time platform workers, while 73 (25.6%) were part-time.

**3.2 Measures**

In this study, all scales used are from established, authoritative journals and have been empirically validated. For the English scales, a "translation-back-translation" method was employed to convert them into Chinese, followed by multiple rounds of empirical testing. The study employs a 5-point Likert scale, asking participants to rate their level of agreement or disagreement with the items (1 = "Strongly Disagree" to 5 = "Strongly Agree").

**Perceived Algorithmic Control (PAC):** This study uses the PAC scale developed by J Pei, S Liu, CuiXun and J Qu [27], consisting of 11 items. A sample item is: "The platform system (algorithm) tracks and locates my geographical position in real-time." The Cronbach's α = 0.903.

**Workplace Objectification (WO):** The WO scale adapted from L Crone, L Brunel and L Auzoult [23] includes 10 items. A sample item is: "The platform cares more about what I can do for them, rather than what they can do for me." The Cronbach's α = 0.944.

**Sustainable Value Co-Creation Behavior (SVCB):** The SVCB scale adapted from K Tu, X Yang, X Su and X Ou [53] consists of 5 items. A sample item is: "In my future work or services, I will continue to provide feedback to the platform that contributes to service improvement." The Cronbach's α = 0.878.

**Algorithm Aversion (AAV):** This study uses the AAV scale developed by C Le, Z Wang and W Kong [54], which consists of 3 items. A sample item is: "The platform’s algorithm knows my preferences so well, it makes me uncomfortable." The Cronbach's α = 0.796. The item statements for the variables are provided in S1 Appendix.

**Control Variables:** Consistent with previous studies [55, 56], this study includes gender, age, education level, job type, and work experience as control variables.

**3.3 Data analysis**

Data analysis for this study was performed using SPSS 27.0 and Mplus 8.3. Initially, confirmatory factor analysis (CFA) and common method bias assessment were conducted with Mplus 8.3. Subsequently, SPSS 27.0 was unutilized perform Harman’s single-factor test, descriptive statistics, correlation analysis, and hierarchical regression analysis. Finally, the Process 4.2 macro was employed to examine the mediation and moderated mediation effects.

**3.4 Analysis results**

**3.4.1 Confirmatory factor analysis**

Confirmatory Factor Analysis (CFA) was conducted using Mplus 8.3 to assess the discriminant validity among the four key variables: PAC, SVCB, WO and AAV.As presented in Table 1, the four-factor model exhibited robust fit indices (χ² = 831.134, CFI = 0.901, TLI = 0.889, RMSEA = 0.068, SRMR = 0.065). Crucially, this model significantly outperformed alternative models, thereby confirming strong discriminant validity across the variables.

**Table 1.** Confirmatory factor analysis results

| **MODEL** | **χ ^2^** | **χ ^2^ / d f** | **CFI** | **TLI** | **RMSEA** | **SRMR** |
| --- | --- | --- | --- | --- | --- | --- |
| Four-factor model：PAC; WO; AAV; SVCB | 831.134 | 2.240^***^ | 0.901 | 0.889 | 0.068 | 0.065 |
| Three-factor model A：PAC+AAV; WO; SVCB | 1255.728 | 3.358^***^ | 0.824 | 0.809 | 0.091 | 0.075 |
| Three-factor model B：PAC; WO+AAV; SVCB | 1293.039 | 3.457^***^ | 0.807 | 0.790 | 0.093 | 0.092 |
| Three-factor model C：PAC+WO; AAV; SVCB | 1976.948 | 5.286^***^ | 0.680 | 0.652 | 0.123 | 0.135 |
| Two-factor model A：PAC+AAV; WO+SVCB | 1628.799 | 4.332^***^ | 0.737 | 0.716 | 0.108 | 0.094 |
| Two-factor model B：PAC; WO+AAV+SVCB | 1806.890 | 4.806^***^ | 0.714 | 0.691 | 0.116 | 0.105 |
| One-factor model：PAC+ WO+AAV+SVCB | 2539.167 | 6.735^***^ | 0.546 | 0.511 | 0.142 | 0.138 |

Notes: N = 285; PAC = Perceived Algorithmic Control, WO = Workplace Objectification, AAV = Algorithm Aversion, SVCB = Sustainable Value Co-creation Behavior, + indicates merging into a single factor, *** p < 0.001.

**3.4.2 Common method bias**

To address common method bias, a Harman's single-factor test was initially performed. This analysis revealed that the first unrotated factor explained 22.657% of the total variance, which is below the commonly accepted 40% threshold, suggesting that CMB is not a significant concern in this study [57], indicating minimal bias. Following PM Podsakoff, SB MacKenzie, J-Y Lee and NP Podsakoff [57] procedure, a latent common method factor was added to the hypothesized four-factor model, creating a common latent factor (CLF) model. Comparison showed no significant improvement in model fit for the five-factor model, with negligible changes in fit indices (ΔCFI = 0.015, ΔTLI = 0.012, ΔRMSEA = 0.002). Consequently, common method bias is not considered a significant concern for the validity of this study's findings.

**3.4.3 Correlation analysis, measure reliability and validity**

The descriptive statistics for the main variables and control variables were analyzed using SPSS 27.0, with results shown in Table 2. All correlations were within a reasonable range, with the highest correlation at 0.580. The maximum variance inflation factor (VIF) was 1.271, indicating no severe multicollinearity. Average variance extracted (AVE) and composite reliability (CR) were also assessed. As shown in Table 3, AVE exceeded 0.5 and CR exceeded 0.7, meeting statistical standards [58], confirming good convergent and discriminant validity. Table 2 also shows a significant positive correlation between PAC and WO (r = 0.421, p < 0.01), and a significant negative correlation between WO and SVCB (r = - 0.499, p < 0.01), providing preliminary support for the hypotheses.

**Table 2. Mean, standard deviation, and correlation coefficients of variables**

| **Variable** | **M** | **SD** | **1** | **2** | **3** | **4** | **5** | **6** | **7** | **8** | **9** |
| --- | --- | --- | --- | --- | --- | --- | --- | --- | --- | --- | --- |
| 1.Gender | 1.380 | 0.531 |  |  |  |  |  |  |  |  |  |
| 2.Age | 2.610 | 0.702 | -0.068 |  |  |  |  |  |  |  |  |
| 3.Education Level | 3.580 | 1.023 | 0.149* | -0.254** |  |  |  |  |  |  |  |
| 4.Work Experience | 3.480 | 1.555 | -0.065 | 0.580** | -0.307** |  |  |  |  |  |  |
| 5.Job Type | 1.260 | 0.437 | -0.077 | 0.467** | -0.280** | 0.460** |  |  |  |  |  |
| 6.PAC | 3.650 | 0.651 | 0.000 | -0.247** | 0.333** | -0.266** | -0.280** | **0.713** |  |  |  |
| 7.WO | 3.320 | 0.911 | -0.058 | -0.255** | 0.268** | -0.255** | -0.243** | 0.421** | **0.817** |  |  |
| 8.AAV | 2.990 | 0.912 | -0.078 | 0.004 | -0.018 | -0.004 | 0.005 | 0.243** | 0.133* | **0.843** |  |
| 9.SVCB | 2.420 | 0.874 | 0.097 | 0.172** | -0.099 | 0.171** | 0.146* | -0.344** | -0.499** | -0.193** | **0.820** |
| Composite Reliability (CR) | | | | | | | | 0.919 | 0.952 | 0.710 | 0.672 |
| Average Variance Extracted (AVE) | | | | | | | | 0.508 | 0.667 | 0.880 | 0.911 |

Notes: N = 285; The coding for certain variables is as follows: Gender: Male = 1, Female = 2; Age: Below 20 = 1, 21-30 years = 2, 31-40 years = 3, 41-50 years = 4, Above 50 = 5; Education Level: Primary school or below = 1, Middle school = 2, General high school/vocational school/technical school/vocational high school = 3, Associate degree = 4, Bachelor's degree or above = 5; Work Experience: Less than 1 year = 1, 1 year (inclusive) to less than 3 years = 2, 3 years (inclusive) to less than 5 years = 3, 5 years (inclusive) to less than 7 years = 4, 7 years (inclusive) to less than 9 years = 5, 9 years or more = 6; Job Type: Full-time = 1, Part-time = 2.

**3.4.4 Hypothesis testing**

**Main effect test.** Hierarchical regression analysis using SPSS 27.0，table 3 indicates that PAC significantly negatively impacted SVCB (Model 3, β = -0.431, p < 0.001), supporting H1.

**Mediation effect test.** As shown in Table 3, PAC significantly positively influenced WO (Model 1, β = 0.466, p < 0.001), and WO significantly negatively affected SVCB (Model 4, β = -0.405, p < 0.001).H2a and H2b is supported.

To test the mediation effect, 5000 bootstrap samples were drawn using the PROCESS 4.2 plugin. Hypotheses 2a and 2b are supported. Additionally, as shown in Table 4, the mediation effect of PAC on SVCB through WO was -0.431, with a 95% confidence interval of [-0.591, -0.271], excluding 0. Thus, H2c is supported.

**Table 3.** Hierarchical regression analysis

| **Variables** | | **WO** | | **SVCB** | |
| --- | --- | --- | --- | --- | --- |
|  |  | **M1** | **M2** | **M3** | **M4** |
|  |  | **β (SE)** | **β (SE)** | **β (SE)** | **β (SE)** |
| **Control Variables** | | |  |  |  |
| Gender | -0.205*（0.100） | | -0.198*（0.098） | 0.265**（0.101） | 0.182（0.093） |
| Age | -0.116（0.087） | | -0.116（0.086） | 0.087（0.088） | 0.040（0.081） |
| Education Level | 0.104*（0.052） | | 0.103*（0.051） | 0.021（0.052） | 0.063（0.048） |
| Work Experience | -0.035（0.040） | | -0.039（0.039） | 0.030（0.040） | 0.016（0.037） |
| Job Type | -0.117（0.131） | | -0.147（0.129） | 0.034（0.132） | -0.014（0.121） |
| **Independent Variable** | | |  |  |  |
| PAC | 0.466***（0.081） | | 0.553***（0.088） | -0.431***（0.081） | -0.243**（0.079） |
| **Mediating Variable** | | |  |  |  |
| WO |  | |  |  | -0.405***（0.055） |
| **Moderating Variable** | | |  |  |  |
| AAV |  | | 0.004（0.056） |  |  |
| **Interaction Term** | | |  |  |  |
| PAC × AAV |  | | 0.279**（0.080） |  |  |
| R^2^ | 0.230 | | 0.265 | 0.141 | 0.283 |
| ΔR^2^ | 0.093 | | 0.033 | 0.085 | 0.142 |
| F | 13.860*** | | 12.468*** | 7.619*** | 15.615*** |

Note: * p < 0.05, ** p < 0.01, *** p < 0.001.

**Table 4. Mediating effect test**

| **Path: PAC-WO-SVCB** | | | |
| --- | --- | --- | --- |
| **Effect Type** | **Effect Value** | **SE** | **95% CI** |
| **Total Effect**  PAC-SVCB | -0.431 | 0.081 | [-0.591, -0.271] |
| **Direct Effect**  PAC-SVCB | -0.243 | 0.079 | [-0.398, -0.088] |
| **Indirect Effect**  PAC-WO-SVCB | -0.189 | 0.044 | [-0.283, -0.113] |

**Moderating effect test.** To investigate the moderating influence of AAV on the association between PAC and WO, both variables were standardized, and an interaction term between AAV and PAC was created to mitigate multicollinearity. Using the PROCESS 4.2 macro, the interaction term significantly predicted WO (Model 2, β = 0.279, p < 0.01), indicating a moderation effect. Further following the approach of KJ Preacher, PJ Curran and DJ Bauer [59]，a simple slope analysis, dividing AAV into high and low levels, fig 2 showed that when AAV was high (M + 1SD), the effect of PAC on WO was stronger (simple slope = 0.832, t = 5.371, p < 0.001), while at low AAV (M - 1SD), the effect was weaker (simple slope = 0.274, t = 0.903, p < 0.05), supporting Hypothesis 3a.

**INSERT FIG 2 HERE**

**Fig 2. Moderation effect of AAV**

**Moderated mediation effect test.** This study assessed whether the indirect effect of PAC on SVCB via WO is moderated by AAV, using PROCESS 4.2 macro. The moderated mediation index was −0.113, with a 95% confidence interval of [−0.184, −0.046], excluding zero, confirming the moderated mediation. As shown in Table 5, when AAV is high, the indirect effect of PAC on SVCB through WO is strongest (−0.327, SE = 0.069, 95% CI [−0.473, −0.201]), with the interval excluding zero. When AAV is low, the indirect effect remains significant but weaker (−0.121, SE = 0.041, 95% CI [−0.213, −0.049]). The difference in mediation effects between high and low AAV is significant (−0.224, SE = 0.047, 95% CI [−0.324, −0.139]), confirming that AAV negatively moderates the indirect effect of PAC on SVCB via WO. Hypothesis 3b is therefore supported.

Table 5. Results of moderated mediation effect test

| **Mediator** | **Moderator** | **Effect Size** | **SE** | **95% CI** |
| --- | --- | --- | --- | --- |
| WO | Low AAV (M-1SD) | -0.121 | 0.041 | [-0.213, -0.049] |
|  | High AAV (M+1SD) | -0.327 | 0.069 | [-0.473 -0.201] |
|  | Difference | -0.224 | 0.047 | [-0.324, -0.139] |

# **4 Study 2: Scenario experiment study**

Although Study 1 collected data at multiple time points, all variables were self-reported, with PAC and AAV measured simultaneously, potentially limiting causal inference and internal validity. Therefore, Study 2 employs a scenario experiment to establish causal evidence for the relationships among PAC, WO, and AAV, thus enhancing internal validity. Specifically, it tests the effect of PAC on WO (H2a) and the moderating role of AAV in this mechanism (H3a).

**4.1 Design and participants**

This study received ethical approval from the Ethics Committee of Nanjing University of Technology (Approval No. NTJTECH-1-21). This experiment employed a between-subjects design with a single factor (PAC experimental group vs. control group). A total of 238 platform workers were recruited in China, informed that the survey was for academic purposes only, fully anonymous, and did not disclose personal information. To enhance sample quality and control for invalid responses, attention-check items (e.g., "Please select 3 for this question") were included, yielding 216 valid samples. Using G*Power [60], the required sample size was calculated. With a medium effect size (d = 0.5), α = 0.05, and 1−β = 0.80, at least 64 participants per group were needed. Therefore, the effective sample size met the standard. Among the valid participants, 38.5% were aged 21–30, 36.9% had 1 year (inclusive) to less than 3 years of work experience, and 63.5% were male.

**4.2 PAC manipulation and pretest**

Drawing on H Wang, Z CHEN, Z LI, Z LIU, C LIANG and B ZHAO [61], we designed article pages discussing or not discussing algorithmic control to manipulate PAC. The PAC experimental group read an article about PAC, while the control group read an unrelated article [62] (See S2 Appendix). A pretest with 136 platform workers confirmed the PAC manipulation's validity.

Independent t-tests showed significant differences (experimental group: M = 3.67, SD = 0.57 vs. control group: M = 2.98, SD = 0.58), t (136) = 6.94, p < 0.001, 95% CI [0.49, 0.88], d = 1.19, confirming successful manipulation of PAC.

**4.3 Experiment procedure and measurement**

To prevent participants from skipping instructions, the "Next" button remained inactive for 20 seconds. Participants were randomly assigned to two groups to read different materials, all validated in the pretest. After reading, participants completed the 11-item PAC measure, followed by assessments of AAV (Cronbach's α = 0.817) and WO (Cronbach's α = 0.830), using a 5-point Likert scale (1 = strongly disagree to 5 = strongly agree). Participants answered demographic questions, including gender, age, education level, and work-related information. Participants received RMB 5 (approx. USD 0.71) for their participation.

**4.4 Manipulation test**

Independent samples t-test results from the main experiment indicated that the PAC score in the experimental group (M = 3.740, SD = 0.350) was significantly higher than that in the control group (M = 3.210, SD = 0.387), t (216) = 10.530, p < 0.001, 95% CI [0.431, 0.630], d = 0.815. This confirms the successful manipulation of PAC.

**4.5 Results**

**4.5.1 Reliability and validity analysis**

To ensure reliability, Cronbach's α, average variance extracted (AVE), and composite reliability (CR) for WO and AAV were assessed. As shown in Table 6, all factor loadings exceeded 0.50 (0.629 – 0.874). AVE > 0.5 and CR > 0.7, meeting C Fornell and DF Larcker [58] standards, confirming good convergent and discriminant validity.

Table 6. Results of reliability and validity analysis

| **Constructs** | **Factor loadings** | **AVE** | **CR** |
| --- | --- | --- | --- |
| **Workplace Objectification** |  | **0.522** | **0.919** |
| WO1 | 0.699 |  |  |
| WO2 | 0.638 |  |  |
| WO3 | 0.743 |  |  |
| WO4 | 0.789 |  |  |
| WO5 | 0.710 |  |  |
| WO6 | 0.638 |  |  |
| WO7 | 0.682 |  |  |
| WO8 | 0.682 |  |  |
| WO9 | 0.629 |  |  |
| WO10 | 0.667 |  |  |
| **Algorithm Aversion** |  | **0.721** | **0.885** |
| AAV1 | 0.851 |  |  |
| AAV2 | 0.821 |  |  |
| AAV3 | 0.874 |  |  |

**4.5.2 Hypothesis testing**

Based on G Casu, MG Mariani, R Chiesa, D Guglielmi and P Gremigni [63], this study included gender, age, education level, and work-related variables as control variables. AAV as the moderator, PAC was the independent variable (coded as a dummy variable: PAC experimental group = 1, control group = 0), and WO was the dependent variable. A multilevel linear regression analysis was conducted.

**H2a test****.** Fig 3 displays the mean levels of WO in the experimental and control groups under PAC. Independent sample t-tests reveal that the experimental group, with PAC, has significantly higher WO (M = 3.390, SD = 0.539) compared to the control group (M = 2.820, SD = 0.508), t (216) = 8.048, p < 0.001, 95% CI [0.433, 0.714], d = 1.097. Thus, H2a is supported.

Further, using the PROCESS 4.2 macro, using 5000 bootstrap samples, table 7 showed that PAC significantly positively affected WO (Model 1, β = 0.502, p < 0.001), confirming support for H2a.

Table 7. Hierarchical regression analysis

| **Variables** | **WO** | | | |
| --- | --- | --- | --- | --- |
|  | **M1** | | **M2** | |
|  | **β** | **SE** | **β** | **SE** |
| **Control Variables** |  |  |  | |
| Gender | -0.069 | 0.040 | -0.048 | 0.040 |
| Age | -0.070 | 0.037 | 0.061 | 0.036 |
| Education level | 0.019 | 0.040 | 0.024 | 0.040 |
| Work experience | 0.054 | 0.031 | 0.044 | 0.031 |
| Daily working hours | 0.017 | 0.031 | -0.001 | 0.031 |
| **Independent Variable** |  |  |  |  |
| PAC | 0.502*** | 0.078 | 0.435*** | 0.083 |
| **Mediating Variable** |  |  |  |  |
| WO |  |  |  |  |
| **Moderating Variable** |  |  |  |  |
| AAV |  |  | 0.089* | 0.045 |
| **Interaction Term** |  |  |  |  |
| PAC × AAV |  |  | 0.195* | 0.090 |
| R^2^ | 0.263 | | 0.297 | |
| ΔR^2^ | 0.147 | | 0.181 | |
| F | 12.401*** | | 10.936*** | |

Notes: * p < 0.05, ** p < 0.01, *** p < 0.001. Daily working hours：Less than 2 hours = 1, 2(inclusive) to less than 4 hours = 2, 4 (inclusive) to less than 8 hours =3, 8 (inclusive) hours or more = 4.

**INSERT FIG 3 HERE**

**Fig 3. Comparison of** WO **between** PAC e**xperimental** **group and** **control group**

**H3a test.** The moderation effect was tested using the PROCESS 4.2 macro, with results shown in Table 7. The interaction term between PAC and AAV significantly predicted WO (Model 2, β = 0.195, p < 0.05), indicating the presence of a moderation effect. A simple slope analysis, following KJ Preacher, PJ Curran and DJ Bauer [59], was conducted, dividing AAV into high and low levels and plotting the interaction effect. As shown in Fig 4, when AAV was high (M + 1SD), the regression slope for PAC and WO was stronger (simple slope = 0.659, t = 3.574, p < 0.001). When AAV was low (M - 1SD), the slope was weaker (simple slope = 0.211, t = 0.594, p > 0.05). These results suggest that the relationship between PAC and WO is positively moderated by AAV, meaning the higher the AAV, the stronger the positive effect of PAC on WO. Therefore, H3a is supported.

**INSERT FIG 4 HERE**

**Fig 4. Moderation effect of AAV**

# **5 Discussion and implication**

**5.1 General discussion**

Many OLPs use algorithmic systems to achieve precise matching between labor supply and demand [1]. Therefore, it is critical to explore the role of PAC in influencing platform workers' SVCB. The specific findings are as follows: First, Study 1, based on a sample of 285 platform workers in China, demonstrates that PAC reduces platform workers' SVCB. WO plays a mediating role between PAC and SVCB. AAV moderates the relationship between PAC, WO, and the mediating effect. Secondly, Study 2 employed a between-subjects design (PAC experimental vs. control group) and recruited an additional 216 platform workers for the formal experiment. Specifically, Study 2 validated the positive impact of PAC on WO and found an interactive effect between PAC and AAV on WO, further supporting the moderating role of AAV.

**5.2 Theoretical implications**

First, this study advances research on PAC by empirically examining its psychological and behavioral effects on platform workers. While earlier work largely adopts qualitative approaches focused on economic rights and interests[34], limited quantitative evidence addresses workers’ subjective experiences. Existing empirical studies emphasize organizational outcomes such as service quality and proactive behavior [27, 64]. By elucidating how PAC undermines SVCB, this study fills a key gap and contributes to theory development in gig economy scholarship.

Second, the study introduces WO as a mediating mechanism, extending objectification theory into algorithmic labor contexts. Prior research has focused mainly on sexual objectification, particularly its impact on women [17, 65] with limited attention to digital work. By validating WO as a pathway through which PAC impairs SVCB, the study offers empirical grounding to debates on algorithmic management’s psychological costs.

Third, this research clarifies the conceptual distinction between WO and organizational dehumanization. While the latter involves denying human attributes through mechanistic or animalistic frames[66, 67], WO focuses on instrumentalization and the loss of autonomy [20, 22]. Differentiating these constructs enhances theoretical precision and informs more ethically attuned platform governance.

Finally, the study identifies AAV as a key moderator, advancing understanding of individual differences in responses to algorithmic control. Previous studies have centered on technological aspects—fairness, privacy, acceptance[68, 69]—while neglecting dispositional factors. By conceptualizing AAV as a stable attitudinal trait moderating the PAC–WO–SVCB linkage, this study extends Trait Activation Theory and the Job Demands–Control Model, while addressing calls for more worker-centric AAV research [70].

**5.3 Managerial implications**

This study highlights the need for OLPs to recognize the adverse effects of PAC on platform workers’ behaviors. While algorithmic control improves efficiency, over-reliance can undermine autonomy and reduce SVCB. OLPs must balance PAC with human-centered management to foster autonomy and engagement. For example, enhancing transparency in task allocation and offering flexible scheduling can improve fairness perceptions and promote participation over passive compliance.

Second, WO is particularly damaging in the gig economy, where workers may feel like mere "tools of production." Addressing WO is critical, as it harms mental health, reduces motivation, and impairs work quality. OLPs should introduce social features like virtual communities and discussion groups to encourage communication, emotional support, and a sense of belonging. Regular assessments of workers’ psychological well-being and providing emotional support are also recommended.

Third, as algorithmic management grows, addressing AAV becomes essential. Resistance stems from mistrust of algorithms, so OLPs should facilitate direct dialogue between workers and algorithm experts to build trust. Two-way feedback mechanisms, allowing workers to contribute to algorithm design, can reduce resistance and improve acceptance.

Finally, investing in training and career development is vital for improving workers’ adaptability and self-awareness. Managers should recognize platform workers as autonomous, capable individuals. Given the evolving demands in platform work, skill development and career support can help workers maintain engagement and enhance their SVCB.

**5.4 Limitations and future directions**

Despite achieving certain results, this study has the following limitations. First, while the sample was drawn from various types of gig platforms across the country, differences in platform management and regional policies may have introduced heterogeneity in the findings. Future research could focus on a single mainstream platform and use longitudinal tracking for targeted comparative studies. Second, the short interval between the two waves of the survey may have led to memory interference from the first survey, affecting data validity. Although the scenario experiment increases external validity, its design limitations may not fully capture real-world behavior. Future studies could extend the interval to reduce memory bias and enhance experimental diversity for greater generalizability. Third, the brief text used in the experiment may not fully reflect the intensity of real platform pressures; richer manipulations, such as live task simulations or VR, could improve future experiments. Finally, this study only considers AAV as a moderator. Future research could explore other individual and organizational variables, such as surface compliance or platform support, to examine how these factors influence the effects of PAC.

# **6 Conclusion**

This study, grounded in objectification theory and trait activation theory, examines how PAC leads to WO and subsequently inhibits platform workers’ SVCB, using a multi-wave survey and scenario experiment. The research further reveals that for workers with high AAV, the negative effects of PAC are more pronounced. The findings suggest that platform managers should consider workers’ attitudes towards algorithms in their algorithmic governance, fostering communication and interaction with workers to mitigate the negative impact of PAC. Overall, this study not only deepens the theoretical understanding of SVCB but also provides valuable insights for the management of PAC, laying a theoretical foundation for future developments in algorithmic management.

# **Acknowledgements**

Thank you to everyone who participated in this research and to those who conducted the surveys for this study.

**Supporting information**

S1 Appendix. Lists of measures

S2 Appendix. Original experiment stimuli in Study 2

**Author contributions**

Conceptualization: Xuan Liu, Yuqing Wang, Haowei Zheng, Yuci Chen.

Data curation: Yuqing Wang, Haowei Zheng.

Investigation: Yuqing Wang, Yuci Chen.

Methodology: Yuqing Wang.

Supervision: Xuan Liu.

Writing – original draft: Yuqing Wang.

Writing – review & editing: Xuan Liu, Yuqing Wang, Haowei Zheng, Yuci Chen

**References**
