# Supplementary material for: How to break free from the "tool" dilemma? A study on the impact of perceived algorithmic control on sustainable value co-creation behavior
Source: PLoS One. 2026 Sep 8;21(9):e0357097. doi: 10.1371/journal.pone.0357097 (PMC13552789; doi:10.1371/journal.pone.0357097)
Supplement: Peer review history

# Peer review: Decision Letter 0

**DOI:** 10.1371/journal.pone.0357097.r001

**Authors:** Peng Zhang

1 Feb 2026

PONE-D-25-44146

How to break free from the "tool" dilemma? A study on the impact of perceived algorithmic control on sustainable value co-creation behavior

PLOS One?

Dear Dr. Wang,

Thank you for submitting your manuscript to PLOS ONE. After careful consideration, we feel that it has merit but does not fully meet PLOS ONE’s publication criteria as it currently stands. Therefore, we invite you to submit a revised version of the manuscript that addresses the points raised during the review process.

Please submit your revised manuscript by Mar 18 2026 11:59PM. If you will need more time than this to complete your revisions, please reply to this message or contact the journal office at plosone@plos.org. When you're ready to submit your revision, log on to https://www.editorialmanager.com/pone/ and select the 'Submissions Needing Revision' folder to locate your manuscript file.

- A letter that responds to each point raised by the academic editor and reviewer(s). You should upload this letter as a separate file labeled 'Response to Reviewers'.
- A marked-up copy of your manuscript that highlights changes made to the original version. You should upload this as a separate file labeled 'Revised Manuscript with Track Changes'.
- An unmarked version of your revised paper without tracked changes. You should upload this as a separate file labeled 'Manuscript'.

If you would like to make changes to your financial disclosure, please include your updated statement in your cover letter. Guidelines for resubmitting your figure files are available below the reviewer comments at the end of this letter.

If applicable, we recommend that you deposit your laboratory protocols in protocols.io to enhance the reproducibility of your results. Protocols.io assigns your protocol its own identifier (DOI) so that it can be cited independently in the future. For instructions see: https://journals.plos.org/plosone/s/submission-guidelines#loc-laboratory-protocols. Additionally, PLOS ONE offers an option for publishing peer-reviewed Lab Protocol articles, which describe protocols hosted on protocols.io. Read more information on sharing protocols at https://plos.org/protocols?utm_medium=editorial-email&utm_source=authorletters&utm_campaign=protocols.

We look forward to receiving your revised manuscript.

Kind regards,

Peng Zhang, Ph.D.

Academic Editor

PLOS One

Journal Requirements:

When submitting your revision, we need you to address these additional requirements.

1. Please ensure that your manuscript meets PLOS ONE's style requirements, including those for file naming. The PLOS ONE style templates can be found at

https://journals.plos.org/plosone/s/file?id=wjVg/PLOSOne_formatting_sample_main_body.pdf and

https://journals.plos.org/plosone/s/file?id=ba62/PLOSOne_formatting_sample_title_authors_affiliations.pdf

2. Please note that PLOS One has specific guidelines on code sharing for submissions in which author-generated code underpins the findings in the manuscript. In these cases, we expect all author-generated code to be made available without restrictions upon publication of the work. Please review our guidelines at https://journals.plos.org/plosone/s/materials-and-software-sharing#loc-sharing-code and ensure that your code is shared in a way that follows best practice and facilitates reproducibility and reuse.

3. When completing the data availability statement of the submission form, you indicated that you will make your data available on acceptance. We strongly recommend all authors decide on a data sharing plan before acceptance, as the process can be lengthy and hold up publication timelines. Please note that, though access restrictions are acceptable now, your entire data will need to be made freely accessible if your manuscript is accepted for publication. This policy applies to all data except where public deposition would breach compliance with the protocol approved by your research ethics board. If you are unable to adhere to our open data policy, please kindly revise your statement to explain your reasoning and we will seek the editor's input on an exemption. Please be assured that, once you have provided your new statement, the assessment of your exemption will not hold up the peer review process.

4. If the reviewer comments include a recommendation to cite specific previously published works, please review and evaluate these publications to determine whether they are relevant and should be cited. There is no requirement to cite these works unless the editor has indicated otherwise.

[Note: HTML markup is below. Please do not edit.]

Reviewers' comments:

Reviewer's Responses to Questions

**Comments to the Author**

1. Is the manuscript technically sound, and do the data support the conclusions?

Reviewer #1: Yes

Reviewer #2: Yes

Reviewer #3: Yes

**********

2. Has the statistical analysis been performed appropriately and rigorously?

Reviewer #1: Yes

Reviewer #2: I Don't Know

Reviewer #3: Yes

**********

3. Have the authors made all data underlying the findings in their manuscript fully available?

The PLOS Data policy

Reviewer #1: Yes

Reviewer #2: Yes

Reviewer #3: Yes

**********

4. Is the manuscript presented in an intelligible fashion and written in standard English?

Reviewer #1: Yes

Reviewer #2: Yes

Reviewer #3: Yes

**********

Reviewer #1: Thanks for allowing me to review the paper How to break free from the "tool" dilemma? A study on the impact of perceived algorithmic control on sustainable value co-creation behavior. This is a well-designed and timely study with strong theoretical and practical contributions. With moderate revisions addressing some points—particularly around conceptual clarity, generalizability, and experimental validity—it would be suitable for publication in PLOS ONE.

1. Conceptual Clarity and Theoretical Contribution

WO vs. Organizational Dehumanization: While the distinction is noted (lines 65–66, 482–485), it could be strengthened. Clarify how WO (instrumentality, disposability) uniquely applies to gig work versus general dehumanization constructs. A brief comparative analysis in the discussion would help. (e.g. Are resources always beneficial? The three-way interaction between slashies’ role stress, self-efficacy and job autonomy. Asia Pacific Journal of Management, 2025, 1-31.)

AAV as a Trait vs. State: The study treats AAV as a stable trait, but algorithmic aversion may be context-dependent or learnable. Acknowledge this limitation and suggest future research on AAV development over time. (e.g. The buffering role of workplace mindfulness: How job insecurity of human-artificial intelligence collaboration impacts employees’ work–life-related outcomes. Journal of Business and Psychology, 2024, 39.6: 1395-1411.)

2. Sample and Generalizability

The sample is limited to Chinese platform workers (e.g., Didi, Meituan). While valuable, findings may not generalize to Western platforms or non-delivery/ride-hailing gig work (e.g., freelance writing, coding). Please confirm this cultural and platform-specific limitation.

Most participants were male (62.1%) and aged 21–40. Consider discussing how gender/age might influence PAC, WO, or SVCB, or control for them more explicitly.

3. Measurement and Common Method Bias

Although Harman’s test and CLF were used (lines 315–324), the two-wave design still collected PAC and AAV at the same time in Wave 1. Consider noting that temporal separation between PAC and AAV could further reduce CMV.

The AAV scale has only 3 items (Cronbach’s α = 0.796). While acceptable, a longer scale might improve reliability. Mention this as a minor limitation.

4. Experimental Design (Study 2)

The scenario-based manipulation of PAC (reading an article) may lack ecological validity compared to real platform interactions. Participants may not fully internalize the algorithmic control experience. Suggest future studies use simulated task platforms or VR.

The control group read an unrelated article. Ensure that the control topic is neutral and does not inadvertently trigger thoughts about algorithms or work control.

5. Causal Claims

While the experiment strengthens causality for PAC → WO, the mediation (WO → SVCB) and full moderated mediation model still rely on correlational survey data. Use cautious language (e.g., “supports a causal pathway” rather than “establishes causality”).

6. Discussion and Implications

Theoretical Implications: Expand on how the study advances trait activation theory in algorithmic contexts. How does AAV interact with situational cues in gig work?

Managerial Implications: Provide more concrete examples: What would “transparent task allocation” look like? How can platforms implement “two-way feedback mechanisms” effectively? (e.g. When employees meet digital-intelligence transformation: Unveiling the role of employee intentions. International Journal of Information Management, 2025, 84: 102912.)

Ethical and Policy Implications: Briefly discuss how regulatory frameworks (e.g., EU AI Act, gig worker laws) could address PAC and WO.

7. Writing and Presentation

Abstract: The abstract repeats the same sentence twice (lines 11–23). Remove the duplication.

Figures: Ensure all figures (Fig 1–4) are clearly referenced in the text. In the PDF, some figures are listed at the end without in-text callouts (e.g., “INSERT FIG HERE” placeholders remain).

References: Some refs are incomplete (e.g., ref 62 lacks title/author). Ensure all references follow PLOS ONE formatting guidelines.

Minor Corrections

Line 11–23: Remove duplicated sentence in abstract.

Line 301: “unutilized” → “used.”

Line 440–441: Table 7 note formatting is misaligned.

Line 527–528: “surface compliance or platform support” — briefly define these if they are proposed as future moderators.

Figure placement: Integrate figures into the main text where referenced.

Reviewer #2: We commend the authors on an interesting pair of studies. The question of platform workers’ well-being is important, and will only become increasingly so as more and more people engage in part-time or full-time platform work. There is no doubt that understanding how workers relate to their jobs in the platform work industry is critical. As the authors themselves are clearly aware, this understanding enables us to better intervene in how platform work is managed.

Comments are divided into major and minor recommendations. Most comments are listed in an order aligned with the paper itself, unless otherwise specified:

Major Comments:

1. We thank the authors for an extremely clear and coherent Introduction section. It did feel at times, however, that concepts and definitions were perhaps repeated more than is needed for readers’ understanding. Authors should feel free to disregard this recommendation, but it may be possible to streamline/shorten the Introduction section a little.

2. One of the claims made by the authors (starting on line 481) is that “this research clarifies the conceptual distinction between WO and organizational dehumanization.” Given the scope of the studies reported here, it is not clear how this conceptual distinction has been made. It is this reviewer’s opinion that this cannot be stated as a major research contribution of the project, or, at the very least, not without additional context:

- In the Introduction, on line 65, the authors write “Unlike organizational dehumanization, which denies human uniqueness, WO emphasizes traits like instrumentality, disposability, lack of autonomy, and violability.” From this explanation alone, it is not clear how organizational dehumanization and WO are distinguished. A more detailed explanation of organization dehumanization (as opposed to WO), would be helpful, especially if it can say more about the specific features that are associated with one but not the other.

- In the discussion, as mentioned above, the authors claim that the conceptual distinction between the two has been made clear. However, in the studies itself, only WO is tested. As far as has been reported, it does not seem that any measures/scales reflecting “organizational humanization” were tested/collected in the two studies. If one claims to have distinguished between the two, then we would expect the two frameworks (WO vs. organizational dehumanization) to be pitted against each other in some manner.

- Especially to those unfamiliar with the relevant literatures, the way WO is described and operationalized can seem very much aligned with lay intuitions of organizational dehumanization.

For instance, in line 500, the authors state: “WO is particularly damaging in the gig economy, where workers may feel like mere "tools of production." This impression of being “tools of production” associated with WO seems very reminiscent of the “mechanistic” frame associated with organizational dehumanization (mentioned in line 482).

Please note that this reviewer isn’t arguing against the idea that WO and organizational dehumanization can be (at least partially) dissociated. Rather, the recommendation is to either: (1) Provide further evidence/background for how this distinction has been made, or to (2) Acknowledge that this distinction cannot yet be fully clarified by the present approach, and perhaps suggest ways this distinction could be clarified by future work.

3. A comprehensive set of analyses were run for the two studies reported here. However, it would generally be helpful to provide a little more context for these analyses, both when describing how they were done and what they tell us:

- For instance, it looks like some of the Control Variables (Gender & Education Level) were significant in some of the models tested (when running the hierarchical regression analyses - lines 341-353). While this reviewer understands that this isn’t amongst the main take-aways of this work, it would be good to acknowledge these findings, and provide some context for what they could mean.

- Some additional specific recommendations are listed in the ‘Minor Comments’ section below.

4. It would be nice to add a short Discussion section at the end of Study 1, recapping the main takeaways of the study before moving on to the Introduction section of Study 2. As mentioned in the previous comment, a comprehensive set of analyses were run. It would be helpful to include a Discussion section here that helps focus the readers’ attention on the main findings the authors care about.

The same applies to the end of Study 2: It would be good to add a brief interim Discussion section summarizing that study’s main take-aways before moving on the General Discussion section.

5. Lines 399-401: It may be good to share a little more how the article pages were designed for the scenario manipulation. The authors do state that the articles were inspired by Wang et al…[61]. However, it would be helpful to provide more context, and to mention what features the manipulation controls for (e.g., length of the texts, difficulty, emotional language, etc.). If these things were not controlled for, it may be good to discuss this as a limitation. (This could be added to the part where the authors discuss the possible external validity of the scenario manipulation (lines 521-522).)

6. Line 489: The authors state that AAV (algorithm aversion) can be conceptualized “as a stable attitudinal trait”.

AAV might be generally stable, but one could imagine that it is not necessarily ‘fixed’. AAV could very well respond to experience. For instance, AAV might be higher as a result of particular work/life experiences.

Given this, could the AAV level have conceivably responded to the PAC manipulation? This could be ruled out by checking for a potential difference between the experimental and control groups. (If this hasn’t already been done.) If there is no difference in AAV between groups, this could be briefly stated. If there is a difference in AAV between groups, it would be good to report this difference, and address what this could mean about the relationship between PAC and AAV. (Note: Apologies if AAV for the two groups was in fact reported and this reviewer missed it!)

----

Minor Comments:

1. It looks like in the submission information that accompanies the manuscript, the abstract was accidentally included twice.

2. Line 85 → “study contributes to…” what? It looks like there are some missing words.

3. Line 98 → “These” : The second "e" is in bold.

4. Line 107 → It would be good to include a citation for the definition of PAC (perceived algorithmic control).

5. Line 109 → Missing comma after “However, SVCB” (Note: SCVB is already clear at this point. Perhaps authors don’t need to repeat its definition here.)

6. Line 267 → What is meant by “inconsistent responses” here? It would be good to quickly clarify.

7. Lines 283-84, 287, 291, 294 → The Cronbach’s alpha is used to compare consistency of responses between the first and second wave, is that correct? It would be good to clarify this at least once, the first time the statistic is reported.

8. Line 301 → Potential mistake here? How about "was utilized to perform" rather than “unutilized perform”.

9. Line 298-303 → It’s a good habit to include citations for the tools used to perform the analyses.

10. Line 310 → Maybe quickly clarify the “alternative models.” E.g. alternative models, in which the variables are grouped into three or fewer factors.

11. Line 328 → It’s usually good form to report p-values that are greater than 0.001. (Not simply state <0.05 or <0.01). If it’s too cumbersome to do in the main manuscript text, these p-values could be included in the appendices.

12. Line 342 → Hypothesis testing section: This may be due to this reviewer’s lack of familiarity with SPSS’ approach to hierarchical regression analyses, but it might be helpful to spend a bit more introducing the analyses that were run. For instance, what is the difference between the 4 models shown in Table 3? Some more context may help readers understand the take-aways better.

13. Line 356 → Do we need to know how the PAC and WO variables were standardized?

14. Lines 436-38 → Hypothesis testing section: Same as the comment above. It would be helpful to understand the difference between the 2 models shown in Table 7. Some more context would clarify the take-aways. (Even if the table shows the difference, a quick explanation in the text would be helpful.)

15. Line 442/Figure 3 → A bar graph may not be the best way to visualize the difference between the experimental and control group WO levels. E.g. A violin plot, box plot or other plots that help show the spread of the data typically do a better job of illustrating differences between groups.

---

We would like to end by acknowledging, once more, the importance of this research. As OLPs become a bigger and bigger part of the labor landscape, research like this will become increasingly meaningful and impactful.

We thank the authors for their work.

Reviewer #3: The manuscript is technically sound and the data support the conclusions of the paper. the data was carried out rigorously well. The authors provided adequate data that make the manuscript to present a detailed ideas and information. The manuscript is presented in standard English language.

**********

what does this mean?). If published, this will include your full peer review and any attached files.

If you choose “no”, your identity will remain anonymous but your review may still be made public.

**Do you want your identity to be public for this peer review?**  For information about this choice, including consent withdrawal, please see our Privacy Policy

Reviewer #1: No

Reviewer #2: No

Reviewer #3: **Yes:** Moses Dele Amosun

**********

[NOTE: If reviewer comments were submitted as an attachment file, they will be attached to this email and accessible via the submission site. Please log into your account, locate the manuscript record, and check for the action link "View Attachments". If this link does not appear, there are no attachment files.]

To ensure your figures meet our technical requirements, please review our figure guidelines: https://journals.plos.org/plosone/s/figures

You may also use PLOS’s free figure tool, NAAS, to help you prepare publication quality figures: https://journals.plos.org/plosone/s/figures#loc-tools-for-figure-preparation.

NAAS will assess whether your figures meet our technical requirements by comparing each figure against our figure specifications.

Attachment Submitted filename: ManuscriptPONE-D-25-44146rvsd.doc

# Author comment: Author response to Decision Letter 1

**DOI:** 10.1371/journal.pone.0357097.r002

16 Mar 2026

Response to reviewers

Reviewer #1: Thanks for allowing me to review the paper How to break free from the "tool" dilemma? A study on the impact of perceived algorithmic control on sustainable value co-creation behavior. This is a well-designed and timely study with strong theoretical and practical contributions. With moderate revisions addressing some points—particularly around conceptual clarity, generalizability, and experimental validity—it would be suitable for publication in PLOS ONE.

Comments to the author:

1.Conceptual Clarity and Theoretical Contribution

WO vs. Organizational Dehumanization: While the distinction is noted (lines 65–66, 482–485), it could be strengthened. Clarify how WO (instrumentality, disposability) uniquely applies to gig work versus general dehumanization constructs. A brief comparative analysis in the discussion would help. (e.g. Are resources always beneficial? The three-way interaction between slashies’ role stress, self-efficacy and job autonomy. Asia Pacific Journal of Management, 2025, 1-31.)

AAV as a Trait vs. State: The study treats AAV as a stable trait, but algorithmic aversion may be context-dependent or learnable. Acknowledge this limitation and suggest future research on AAV development over time. (e.g. The buffering role of workplace mindfulness: How job insecurity of human-artificial intelligence collaboration impacts employees’ work–life-related outcomes. Journal of Business and Psychology, 2024, 39.6: 1395-1411.)

Authors’ response:

We sincerely thank the reviewer for the constructive comments regarding conceptual clarity and theoretical refinement. In response to these suggestions, we have carefully revised the manuscript to further strengthen the theoretical distinction between workplace objectification (WO) and organizational dehumanization, and to provide a more cautious and precise conceptualization of algorithm aversion (AAV).

First, regarding the distinction between workplace objectification and organizational dehumanization, we have added a clearer theoretical differentiation in the Introduction (Lines 58–86) to explain why workplace objectification is more appropriate for capturing the psychological experience of platform workers under algorithmic control. We also revised the Discussion section (Lines 664–678) to provide a brief comparison between the two constructs. In addition, we clarified this issue in the Limitations and Future Directions section (Lines 834–841) by acknowledging the related limitation. Specifically, we clarified that organizational dehumanization refers to a broader perception that the organization denies employees’ human qualities, whereas workplace objectification focuses on the experience of being treated as an instrumental tool for task execution, which is particularly salient in algorithmically managed gig work.

Second, regarding the conceptualization of algorithm aversion, we carefully considered the reviewer’s point that this construct may be context-dependent and potentially learnable. Accordingly, we refined the definition of algorithm aversion in the Introduction (Lines 87–114) to make it more precise. We also added discussion in the Limitations and Future Directions section (Lines 848–864), where we acknowledge the context-dependent nature of algorithm aversion and call for future research to adopt longitudinal designs or experience sampling methods to examine its dynamic evolution, as well as whether its moderating role may vary across different stages of algorithm adaptation.

The relevant revisions have been highlighted in yellow in the manuscript. Thank you again for your valuable suggestion.

Comments to the author:

2. Sample and Generalizability

The sample is limited to Chinese platform workers (e.g., Didi, Meituan). While valuable, findings may not generalize to Western platforms or non-delivery/ride-hailing gig work (e.g., freelance writing, coding). Please confirm this cultural and platform-specific limitation.

Most participants were male (62.1%) and aged 21–40. Consider discussing how gender/age might influence PAC, WO, or SVCB, or control for them more explicitly.

Authors’ response:

We have revised the manuscript accordingly. In fact, both Study 1 and Study 2 were based exclusively on Chinese platform workers (e.g., workers from DiDi and Meituan). We have now clarified in the Sample and Procedure section of Study 1 (Lines 291–292), as well as in the Design and Participants section of Study 2 (Line 490-491), that the samples in this research focus on Chinese ride-hailing and food delivery platform workers. We have also added a corresponding statement in the Limitations and Future Directions section (Lines 788–808) to acknowledge the limitations related to the sample scope of the present study.

In addition, we agree that demographic variables such as gender and age may introduce potential bias into the results. To address this concern, we made two revisions. First, we re-ran the regression analyses using a hierarchical regression approach (see the revised Tables 3 and 7 for details). In Model 1 and Model 4, we included all demographic variables (e.g., gender, age, and education) as control variables. The results show that, after controlling for these demographic factors, the effects of perceived algorithmic control (PAC) on workplace objectification (WO) and sustainable value co-creation behavior (SVCB) remained highly significant. Second, we added brief interim discussions at the end of Study 1 (Lines 448–477) and Study 2 (Lines 602–620), respectively, to further interpret the findings at each stage.

The relevant revisions have been highlighted in yellow in the manuscript. Thank you for your suggestion.

Comments to the author:

3.Measurement and Common Method Bias

Although Harman’s test and CLF were used (lines 315–324), the two-wave design still collected PAC and AAV at the same time in Wave 1. Consider noting that temporal separation between PAC and AAV could further reduce CMV.

The AAV scale has only 3 items (Cronbach’s α = 0.796). While acceptable, a longer scale might improve reliability. Mention this as a minor limitation.

Authors’ response:

We appreciate the reviewer’s valuable comment on this issue. In fact, although PAC and AAV were both measured in the first wave during the initial questionnaire design, the survey procedure was structured in a way that helped reduce potential bias. Specifically, demographic information and algorithm aversion were measured first, whereas perceived algorithmic control was measured at the end of the questionnaire. In addition, in the instruction for the algorithm aversion measure, participants were explicitly asked to respond based on their “overall and stable feelings and attitudes toward algorithmic technology over time,” rather than on situational or momentary emotional reactions. We have added this clarification to the Sample and Procedure section (Lines 298–303).

At the same time, in the Limitations and Future Directions section (Lines 809–820), we acknowledge that collecting these variables at different time points could further reduce potential common method variance, and we suggest that future research may adopt a multi-wave design to separate the measurement of the independent variable and the moderator.

In addition, regarding the algorithm aversion (AAV) scale, which contains only three items (Cronbach’s α = 0.796), we agree that the relatively small number of items may to some extent limit construct coverage and measurement precision. Accordingly, we have added a corresponding statement in the Limitations and Future Directions section (Lines 842–847).

The relevant revisions have been highlighted in yellow in the manuscript. Thank you again for your valuable suggestion.

Comments to the author:

4.Experimental Design (Study 2)

The scenario-based manipulation of PAC (reading an article) may lack ecological validity compared to real platform interactions. Participants may not fully internalize the algorithmic control experience. Suggest future studies use simulated task platforms or VR.

The control group read an unrelated article. Ensure that the control topic is neutral and does not inadvertently trigger thoughts about algorithms or work control.

Authors’ response:

First, we agree that a scenario-based experimental manipulation may not fully capture the complexity and psychological intensity of real platform interactions. Therefore, we have addressed this issue in the Limitations and Future Directions section (Lines 821–833).

Second, regarding the control group materials, some related details were omitted in the original manuscript due to word limit constraints. In the revised manuscript, we have added a description of the development and selection process of the experimental materials in the PAC Manipulation and Pretest section (Lines 499–514). Specifically, the experimental materials were adapted from prior studies, and were further evaluated and revised through internal discussions and with the assistance of two doctoral students, in order to ensure their cultural appropriateness.

The relevant revisions have been highlighted in yellow in the manuscript. Thank you again for your valuable suggestion.

Comments to the author:

5. Causal Claims

While the experiment strengthens causality for PAC → WO, the mediation (WO → SVCB) and full moderated mediation model still rely on correlational survey data. Use cautious language (e.g., “supports a causal pathway” rather than “establishes causality”).

Authors’ response:

In response to this suggestion, we have made consistent revisions throughout the manuscript. Specifically, when referring to Study 1 and the full model, we adopted more cautious expressions to avoid overstating the findings. For Study 2, we revised the wording to indicate that it “provides more direct causal evidence for the relationship between PAC and WO,” while avoiding overly strong claims such as “proves” or “establishes causality.”

In addition, we further clarified in the discussion of Study 1, the discussion of Study 2, and the General Discussion the distinction between the scope of causal inference that can be supported by the experimental study and the correlational nature of the evidence provided by the survey study. The relevant revisions have been highlighted in the manuscript. Thank you again for your valuable suggestion.

Comments to the author:

6.Discussion and Implications

Theoretical Implications: Expand on how the study advances trait activation theory in algorithmic contexts. How does AAV interact with situational cues in gig work?

Managerial Implications: Provide more concrete examples: What would “transparent task allocation” look like? How can platforms implement “two-way feedback mechanisms” effectively? (e.g. When employees meet digital-intelligence transformation: Unveiling the role of employee intentions. International Journal of Information Management, 2025, 84: 102912.)

Ethical and Policy Implications: Briefly discuss how regulatory frameworks (e.g., EU AI Act, gig worker laws) could address PAC and WO.

Authors’ response:

Regarding the theoretical implications, we revised and enriched the relevant content in response to your suggestion. First, in the Introduction (Lines 87–113), we strengthened the logical connection among the high-demand/low-control platform labor context, algorithm aversion, and trait activation theory. We also expanded the Theoretical Implications section (Lines 679–702) to clarify how this study extends trait activation theory in the context of platform algorithmic management, and how algorithm aversion interacts with situational cues in gig work.

Regarding the managerial implications, we also revised and refined this part by adding more specific and actionable practical suggestions.

With respect to the ethical and policy implications, we added a fifth paragraph to the Managerial Implications section in the revised manuscript (Lines 770–786) to provide a brief discussion of these issues.

The relevant revisions have been highlighted in the manuscript. Thank you again for your valuable suggestion.

Comments to the author:

7.Writing and Presentation

Abstract: The abstract repeats the same sentence twice (lines 11–23). Remove the duplication.

Figures: Ensure all figures (Fig 1–4) are clearly referenced in the text. In the PDF, some figures are listed at the end without in-text callouts (e.g., “INSERT FIG HERE” placeholders remain).

References: Some refs are incomplete (e.g., ref 62 lacks title/author). Ensure all references follow PLOS ONE formatting guidelines.

Minor Corrections

Line 11–23: Remove duplicated sentence in abstract.

Line 301: “unutilized” → “used.”

Line 440–441: Table 7 note formatting is misaligned.

Line 527–528: “surface compliance or platform support” — briefly define these if they are proposed as future moderators.

Figure placement: Integrate figures into the main text where referenced.

Authors’ response:

We sincerely thank the reviewer for the careful examination of the manuscript’s formatting, figures, and references. We have revised each of these issues point by point in accordance with your suggestions.

We checked and standardized the in-text citations of Figures 1–4, removed placeholders such as “INSERT FIG HERE” from the original manuscript, and integrated the figures into the main text at the appropriate points of reference.

We also conducted a comprehensive review and supplementation of the references, corrected incomplete entries, and revised the reference list to conform to the formatting requirements of PLOS ONE.

The duplicated sentence in the abstract has been deleted.

The word “unutilized” in Line 301 has been revised.

The note format in Table 7 has been reformatted and consistently aligned.

Brief definitions of “surface compliance” and “platform support” have been added to improve clarity of expression.

The relevant revisions have been highlighted in the manuscript. Thank you again for your valuable suggestion.

Reviewer #2: We commend the authors on an interesting pair of studies. The question of platform workers’ well-being is important, and will only become increasingly so as more and more people engage in part-time or full-time platform work. There is no doubt that understanding how workers relate to their jobs in the platform work industry is critical. As the authors themselves are clearly aware, this understanding enables us to better intervene in how platform work is managed.

Comments are divided into major and minor recommendations. Most comments are listed in an order aligned with the paper itself, unless otherwise specified:

Major Comments:

Comments to the author:

1. We thank the authors for an extremely clear and coherent Introduction section. It did feel at times, however, that concepts and definitions were perhaps repeated more than is needed for readers’ understanding. Authors should feel free to disregard this recommendation, but it may be possible to streamline/shorten the Introduction section a little.

Authors’ response:

We appreciate the reviewer’s suggestion. While we retained most of the introduction to preserve conceptual clarity, we carefully reviewed the section and removed several repetitive sentences to improve conciseness.The relevant revisions have been highlighted in the manuscript.

Comments to the author:

2. One of the claims made by the authors (starting on line 481) is that “this research clarifies the conceptual distinction between WO and organizational dehumanization.” Given the scope of the studies reported here, it is not clear how this conceptual distinction has been made. It is this reviewer’s opinion that this cannot be stated as a major research contribution of the project, or, at the very least, not without additional context:

- In the Introduction, on line 65, the authors write “Unlike organizational dehumanization, which denies human uniqueness, WO emphasizes traits like instrumentality, disposability, lack of autonomy, and violability.” From this explanation alone, it is not clear how organizational dehumanization and WO are distinguished. A more detailed explanation of organization dehumanization (as opposed to WO), would be helpful, especially if it can say more about the specific features that are associated with

Attachment Submitted filename: Response to reviewers.docx

# Peer review: Decision Letter 1

**DOI:** 10.1371/journal.pone.0357097.r003

**Authors:** Peng Zhang

6 May 2026

PONE-D-25-44146R1

How to break free from the "tool" dilemma? A study on the impact of perceived algorithmic control on sustainable value co-creation behavior

PLOS One?

Dear Dr. Wang,

Please submit your revised manuscript by Jun 20 2026 11:59PM. If you will need more time than this to complete your revisions, please reply to this message or contact the journal office at plosone@plos.org. When you're ready to submit your revision, log on to https://www.editorialmanager.com/pone/ and select the 'Submissions Needing Revision' folder to locate your manuscript file.

- A letter that responds to each point raised by the academic editor and reviewer(s). You should upload this letter as a separate file labeled 'Response to Reviewers'.
- A marked-up copy of your manuscript that highlights changes made to the original version. You should upload this as a separate file labeled 'Revised Manuscript with Track Changes'.
- An unmarked version of your revised paper without tracked changes. You should upload this as a separate file labeled 'Manuscript'.

If you would like to make changes to your financial disclosure, please include your updated statement in your cover letter. Guidelines for resubmitting your figure files are available below the reviewer comments at the end of this letter.

If applicable, we recommend that you deposit your laboratory protocols in protocols.io to enhance the reproducibility of your results. Protocols.io assigns your protocol its own identifier (DOI) so that it can be cited independently in the future. For instructions see: https://journals.plos.org/plosone/s/submission-guidelines#loc-laboratory-protocols. Additionally, PLOS ONE offers an option for publishing peer-reviewed Lab Protocol articles, which describe protocols hosted on protocols.io. Read more information on sharing protocols at https://plos.org/protocols?utm_medium=editorial-email&utm_source=authorletters&utm_campaign=protocols.

As the corresponding author, your ORCID iD is verified in the submission system and will appear in the published article. PLOS supports the use of ORCID, and we encourage all coauthors to register for an ORCID iD and use it as well. Please encourage your coauthors to verify their ORCID iD within the submission system before final acceptance, as unverified ORCID iDs will not appear in the published article. *Only*  the individual author can complete the verification step; PLOS staff *cannot*  verify ORCID iDs on behalf of authors.

We look forward to receiving your revised manuscript.

Kind regards,

Peng Zhang, Ph.D.

Academic Editor

PLOS One

Journal Requirements:

If the reviewer comments include a recommendation to cite specific previously published works, please review and evaluate these publications to determine whether they are relevant and should be cited. There is no requirement to cite these works unless the editor has indicated otherwise.

Please review your reference list to ensure that it is complete and correct. If you have cited papers that have been retracted, please include the rationale for doing so in the manuscript text, or remove these references and replace them with relevant current references. Any changes to the reference list should be mentioned in the rebuttal letter that accompanies your revised manuscript. If you need to cite a retracted article, indicate the article’s retracted status in the References list and also include a citation and full reference for the retraction notice.

[Note: HTML markup is below. Please do not edit.]

Reviewers' comments:

Reviewer's Responses to Questions

**Comments to the Author**

Reviewer #1: (No Response)

Reviewer #2: (No Response)

**********

2. Is the manuscript technically sound, and do the data support the conclusions?

Reviewer #1: Yes

Reviewer #2: Yes

**********

3. Has the statistical analysis been performed appropriately and rigorously?

Reviewer #1: Yes

Reviewer #2: Yes

**********

4. Have the authors made all data underlying the findings in their manuscript fully available?

The PLOS Data policy

Reviewer #1: No

Reviewer #2: (No Response)

**********

5. Is the manuscript presented in an intelligible fashion and written in standard English?

Reviewer #1: Yes

Reviewer #2: Yes

**********

Reviewer #1: The revised manuscript has improved substantially compared to the previous version. The authors have responded carefully to most of the reviewers’ comments, particularly in clarifying conceptual distinctions (e.g., workplace objectification vs. organizational dehumanization), refining causal language, and strengthening methodological transparency. The addition of limitations, interim discussions, and improved reporting of analyses has enhanced the rigor and readability of the paper.

Overall, the manuscript is now much clearer, more balanced, and closer to publication quality. That said, I still have several suggestions that could further strengthen the theoretical contribution and positioning of the study.

1. Theoretical Contribution Still Needs Stronger Positioning

While the authors have addressed prior concerns, the theoretical contribution remains somewhat underdeveloped or conservatively framed. The manuscript currently positions its contribution mainly as: applying objectification theory to gig work, extending trait activation theory into algorithmic contexts. However, this positioning still reads more as application/extension rather than theory advancement.

I recommend the authors more explicitly articulate: What new mechanism is revealed beyond existing literature?

How does algorithmic control reshape traditional psychological processes?

What is uniquely theoretical (not just empirical) about combining PAC + WO + AAV?

2. The authors have made a good effort to clarify the distinction between workplace objectification (WO) and organizational dehumanization, and have toned down overly strong claims. However, the distinction is still largely conceptual and narrative. There is no empirical differentiation between the constructs.

3. Missing Integration of Relevant Literature (Important). One important issue is that some relevant and recent literature (previously suggested) has not been incorporated into the manuscript. I recommend some related articles maybe you can cited in your research, such as: Are resources always beneficial? The three-way interaction between slashies’ role stress, self-efficacy and job autonomy. Asia Pacific Journal of Management, 2025, 1-31.; When employees meet digital-intelligence transformation: Unveiling the role of employee intentions. International Journal of Information Management, 2025, 84: 102912.; Exploring the impact of forced teleworking on counterproductive work behavior: the role of event strength and work-family conflict. Internet Research, 2026, 36.1: 53-77.

Reviewer #2: We commend the authors on their thorough revisions to the manuscript. The issues brought up in the previous round of reviews have been carefully addressed.

---

There are a few minor corrections recommended:

- Line 65 → Replacing "workers being experienced as..." with "workers being treated as..." might be more appropriate for this sentence.

- Line 90 → Typo: the "pace" of work should be the "place" of work

- Line 138 → The "e" in "These" is in bold

- Line 422 → There seems to be a missing word: “and entered *in* the regression model”

- Lines 673-676 → The authors state that "At the same time, this study also theoretically demonstrates that, in the context of platform algorithmic management, WO is better able to capture workers lived perceptions of instrumentality and replaceability than the broader concept of organizational dehumanization".

Given that dehumanization and WO are not pitted against each other in the design of the studies, it may be good to avoid stating this too strongly here. This reviewer recognizes that this was addressed in the limitations section later on in the paper, but it may also be good to avoid this here as well.

---

Once again, we applaud the authors on their thorough revisions!

**********

what does this mean?). If published, this will include your full peer review and any attached files.

If you choose “no”, your identity will remain anonymous but your review may still be made public.

**Do you want your identity to be public for this peer review?**  For information about this choice, including consent withdrawal, please see our Privacy Policy

Reviewer #1: No

Reviewer #2: No

**********

[NOTE: If reviewer comments were submitted as an attachment file, they will be attached to this email and accessible via the submission site. Please log into your account, locate the manuscript record, and check for the action link "View Attachments". If this link does not appear, there are no attachment files.]

To ensure your figures meet our technical requirements, please review our figure guidelines: https://journals.plos.org/plosone/s/figures

You may also use PLOS’s free figure tool, NAAS, to help you prepare publication quality figures: https://journals.plos.org/plosone/s/figures#loc-tools-for-figure-preparation.

NAAS will assess whether your figures meet our technical requirements by comparing each figure against our figure specifications.

# Author comment: Author response to Decision Letter 2

**DOI:** 10.1371/journal.pone.0357097.r004

30 May 2026

Response to reviewers

Reviewer #1: The revised manuscript has improved substantially compared to the previous version. The authors have responded carefully to most of the reviewers’ comments, particularly in clarifying conceptual distinctions (e.g., workplace objectification vs. organizational dehumanization), refining causal language, and strengthening methodological transparency. The addition of limitations, interim discussions, and improved reporting of analyses has enhanced the rigor and readability of the paper.

Overall, the manuscript is now much clearer, more balanced, and closer to publication quality. That said, I still have several suggestions that could further strengthen the theoretical contribution and positioning of the study.

mini

Comments to the author:

1.Theoretical Contribution Still Needs Stronger Positioning

While the authors have addressed prior concerns, the theoretical contribution remains somewhat underdeveloped or conservatively framed. The manuscript currently positions its contribution mainly as: applying objectification theory to gig work, extending trait activation theory into algorithmic contexts. However, this positioning still reads more as application/extension rather than theory advancement.

I recommend the authors more explicitly articulate: What new mechanism is revealed beyond existing literature?

How does algorithmic control reshape traditional psychological processes?

What is uniquely theoretical (not just empirical) about combining PAC + WO + AAV?

Authors’ response:

We sincerely thank the reviewer for the constructive suggestions regarding the theoretical contribution. Based on your suggestions, we have revised the marginal contribution section in the Introduction (lines 133–134 and 139–142 in the revised manuscript) and the Theoretical Implications section to more clearly articulate the theoretical contribution of this paper relative to existing research on perceived algorithmic control. We have clarified that the core contribution of this paper is to reveal how perceived algorithmic control transforms workers’ evaluations of algorithmic systems into their instrumentalized self-understanding and self-construction in the platform labor process.

First, regarding the new mechanism revealed in this paper, we have added and strengthened the following statements in the Theoretical Implications section:“Existing research on algorithmic management has often explained workers’ negative reactions from the perspective of how they evaluate algorithmic systems or platform management practices, such as whether algorithms are perceived as fair, transparent, autonomy-restricting, or stressful. These studies mainly focus on workers’ evaluations of external algorithmic systems or organizational management practices, whereas this study shifts attention to how platform workers understand their own position in the labor process under algorithmic management.”

We have also added the following clarification:“In other words, algorithmic control not only affects how workers evaluate algorithms, but may also change how they understand themselves, leading them to view themselves as usable, controllable, and replaceable functional units within the platform system. Thus, WO captures a psychological process through which algorithmic control is internalized into workers’ self-understanding.”

Through the above revisions, we have made clear that the psychological mechanism revealed in this paper is that perceived algorithmic control may further change platform workers’ self-perception in their own labor process, leading them to experience workplace objectification as being treated instrumentally.

Second, regarding how algorithmic control affects traditional psychological processes, we have added the following statement:“Therefore, while this study examines WO as a mediating variable, more importantly, it reveals a psychological mechanism of algorithmic control that differs from existing explanations. PAC may weaken SVCB by changing workers’ understanding of their own position in the labor process, leading them to experience themselves as being treated as instruments.” The theoretical contribution of this paper is to advance the explanation of the negative effects of algorithmic control from how platform workers evaluate algorithms or platform management to how platform workers re-understand their own labor process under algorithmic control.

Finally, regarding the theoretical integration logic among PAC, WO, and AAV, we have added the following statement in the Theoretical Implications section:“Technologized and depersonalized algorithmic control, as an important situational cue, can activate individuals’ pre-existing attitudes toward algorithms and influence whether external algorithmic control is transformed into an instrumentalized understanding of the self. In this process, PAC represents the technologized situational cue in platform algorithmic management, WO reflects workers’ instrumentalized self-understanding under this cue, and AAV explains why this self-oriented psychological response differs across workers.”

Through the above revisions, we have more explicitly responded to the reviewer’s three concerns: First, the new mechanism revealed in this paper is that perceived algorithmic control changes workers’ understanding of their own labor position through workplace objectification; second, perceived algorithmic control not only affects how workers evaluate algorithms, but may also change how they view themselves; third, the theoretical integration of PAC, WO, and AAV explains how perceived algorithmic control is transformed into instrumentalized self-understanding and among which workers such transformation is more likely to occur.

The relevant changes have been made in the marginal contribution section of the Introduction and the Theoretical Implications section (lines 660–760) and have been highlighted in yellow in the manuscript. Thank you for your suggestion.

Comments to the author:

The authors have made a good effort to clarify the distinction between workplace objectification (WO) and organizational dehumanization, and have toned down overly strong claims. However, the distinction is still largely conceptual and narrative. There is no empirical differentiation between the constructs.

Authors’ response:

We thank the reviewer for raising this important concern. We agree with the reviewer’s assessment that, although this study theoretically discussed the distinction between workplace objectification (WO) and organizational dehumanization, because we did not measure both constructs simultaneously within the same research design, we are unable to empirically test their empirical boundary in the algorithmic management context through discriminant validity tests, confirmatory factor analysis, incremental validity tests, or competing model comparisons. Based on your suggestion, we have revised the Theoretical Implications section and the Limitations and Future Directions section, and re examined the relevant discussion of WO throughout the manuscript to avoid conflating WO with organizational dehumanization.

First, in the Theoretical Implications section, we have clarified the theoretical boundary between WO and organizational dehumanization. Specifically, we have added and revised the following statements:“Organizational dehumanization places greater emphasis on employees’ external evaluation of the organization or management system, that is, employees feel that the organization denies their human attributes, such as emotion, warmth, individuality, and agency, and treats them as objects lacking full human qualities. In contrast, WO places greater emphasis on individuals’ instrumentalized self experience in the labor process, that is, workers feel that they are merely tools, resources, components, or functional units for achieving organizational or platform goals.”

And we have added a clarification of the key distinction between the two:“Therefore, the key distinction between the two concepts is that organizational dehumanization focuses more on how workers evaluate whether the organization or management system denies their humanity, whereas WO focuses more on how workers understand their own position in the labor system, especially whether they experience themselves as usable, controllable, and replaceable instrumental beings.”

We have made clear that organizational dehumanization focuses more on how workers evaluate whether the organization or management system denies their humanity, whereas WO focuses more on how workers understand their own position in the labor system, especially whether they experience themselves as usable, controllable, and replaceable instrumental beings.

Second, in the Theoretical Implications section, we have further explained why this study adopts WO as the theoretical entry point, and we have avoided stating that this study has empirically proven that WO is superior to organizational dehumanization. We have added and revised the following statements:“Therefore, this study adopts WO as the theoretical lens to more accurately explain how algorithmic management leads platform workers to develop the subjective experience that I am merely an instrumental resource within the system.”

What this study focuses on is how perceived algorithmic leads platform workers to gradually understand themselves as executive nodes, functional components, or instrumental extensions within the algorithmic system.

Finally, we agree with the reviewer’s comment that “there is no empirical difference between these two constructs.” Because this study did not measure WO and organizational dehumanization simultaneously within the same research design, we are unable to directly test their empirical boundary in the algorithmic management context through discriminant validity tests, incremental validity tests, or competing model comparisons. Therefore, we have revised the relevant statements in the manuscript to avoid presenting the distinction between WO and organizational dehumanization as a conclusion that has been empirically supported by this study, and instead present it only as the conceptual basis for the theoretical choice in this study.

At the same time, we have added a discussion of this limitation in the Limitations and Future Directions section, stating that this study did not empirically distinguish between WO and organizational dehumanization within the same research design:“This study did not measure WO and organizational dehumanization simultaneously within the same research design, but only distinguished them theoretically. Therefore, it cannot directly test their empirical boundary in the context of algorithmic management.”

And we have noted that future research could include both WO and organizational dehumanization and use discriminant validity tests, incremental validity tests, competing model comparisons, and analyses of differentiated mechanisms to further clarify the relationship and distinction between the two constructs in the context of platform algorithmic management:“Future research could include both WO and organizational dehumanization and use discriminant validity tests, incremental validity tests, competing model comparisons, and analyses of differentiated mechanisms to further clarify the relationship and distinction between the two constructs in the context of platform algorithmic management.”

The relevant changes have been made in the Theoretical Implications section (lines 685–707) and the Limitations and Future Directions section (lines 892–904) and have been highlighted in yellow in the manuscript. Thank you for your suggestion.

Comments to the author:

3. Missing Integration of Relevant Literature (Important). One important issue is that some relevant and recent literature (previously suggested) has not been incorporated into the manuscript. I recommend some related articles maybe you can cited in your research, such as: Are resources always beneficial? The three-way interaction between slashies’ role stress, self-efficacy and job autonomy. Asia Pacific Journal of Management, 2025, 1-31.; When employees meet digital-intelligence transformation: Unveiling the role of employee intentions. International Journal of Information Management, 2025, 84: 102912.; Exploring the impact of forced teleworking on counterproductive work behavior: the role of event strength and work-family conflict. Internet Research, 2026, 36.1: 53-77.

Authors’ response:

We thank the reviewer for this important suggestion. Based on the reviewer's recommendation, we have added and integrated the relevant literature in the revised manuscript.

First, in the Introduction section, we cite Wu et al. (2025) to illustrate that when digital and intelligent technologies intervene in the work process, individuals' attitudes, intentions, and perceived control affect their behavioral responses to technological change (lines 55–57); in the hypothesis development section on the effect of perceived algorithmic control on sustained value co-creation behavior, we cite Duan et al. (2025) to support the view that autonomy is an important work resource in the context of flexible employment (lines 179–182); in the hypothesis development section on the moderating role of algorithm aversion, we cite Liang et al. (2026) to illustrate that salient work events may trigger psychological stress and affect subsequent behavioral responses (lines 266–271). Second, we have also added a citation to Lin et al. (2025), a recent study on perceived algorithmic control and gig workers' work engagement, to further connect the latest literature on algorithmic control and platform workers' psychological and behavioral consequences (lines 57–60).

The relevant changes have been highlighted in yellow in the manuscript. Thank you for your suggestion.

Reviewer #2: We commend the authors on their thorough revisions to the manuscript. The issues brought up in the previous round of reviews have been carefully addressed.

Major Comments:

Comments to the author:

There are a few minor corrections recommended:

- Line 65 → Replacing "workers being experienced as..." with "workers being treated as..." might be more appropriate for this sentence.

- Line 90 → Typo: the "pace" of work should be the "place" of work

- Line 138 → The "e" in "These" is in bold

- Line 422 → There seems to be a missing word: “and entered *in* the regression model”

- Lines 673-676 → The authors state that "At the same time, this study also theoretically demonstrates that, in the context of platform algorithmic management, WO is better able to capture workers lived perceptions of instrumentality and replaceability than the broader concept of organizational dehumanization".

Given that dehumanization and WO are not pitted against each other in the design of the studies, it may be good to avoid stating this too strongly here. This reviewer recognizes that this was addressed in the limitations section later on in the paper, but it may also be good to avoid this here as well.

---

Once again, we applaud the authors on their thorough revisions!

Authors’ response:

Thank you very much for your careful review of our manuscript and for your positive recognition of our previous revisions. We greatly appreciate your constructive comments, which have helped us further improve the clarity and rigor of our paper.

In response to your comments, we have made the following revisions to the manuscript:

First, the sentence on line 65 has been revised from "workers being experienced as" to "workers being treated as" to improve clarity.

Second, the spelling error on line 90 has been corrected, changing "pace" to "place of work."

Third, on line 138, the extra bolded letter "e" in the word "These" has been removed.

Fourth, on line 422, the missing word "in" has been added, and the phrase now reads "and entered in the regression model."

Finally, regarding the comparison between workplace objectification (WO) and organizational dehumanization on lines 673–676. We agree with the reviewer's assessment that, because the research design of this study did not directly compare WO and organizational dehumanization, it is inappropriate to make a strong claim that WO is superior to organiza

Attachment Submitted filename: Response_to_reviewers_auresp_2.docx

# Peer review: Decision Letter 2

**DOI:** 10.1371/journal.pone.0357097.r005

**Authors:** Peng Zhang

29 Jun 2026

PONE-D-25-44146R2

How to break free from the "tool" dilemma? A study on the impact of perceived algorithmic control on sustainable value co-creation behavior

PLOS One?

Dear Dr. Wang,

Thank you for submitting your manuscript to PLOS ONE. After careful consideration, we feel that it has merit but does not fully meet PLOS ONE’s publication criteria as it currently stands. Therefore, we invite you to submit a revised version of the manuscript that addresses the points raised during the review process.

Please submit your revised manuscript by Aug 13 2026 11:59PM. If you will need more time than this to complete your revisions, please reply to this message or contact the journal office at plosone@plos.org. When you're ready to submit your revision, log on to https://www.editorialmanager.com/pone/ and select the 'Submissions Needing Revision' folder to locate your manuscript file.

- A letter that responds to each point raised by the academic editor and reviewer(s). You should upload this letter as a separate file labeled 'Response to Reviewers'.
- A marked-up copy of your manuscript that highlights changes made to the original version. You should upload this as a separate file labeled 'Revised Manuscript with Track Changes'.
- An unmarked version of your revised paper without tracked changes. You should upload this as a separate file labeled 'Manuscript'.

If you would like to make changes to your financial disclosure, please include your updated statement in your cover letter. Guidelines for resubmitting your figure files are available below the reviewer comments at the end of this letter.

If applicable, we recommend that you deposit your laboratory protocols in protocols.io to enhance the reproducibility of your results. Protocols.io assigns your protocol its own identifier (DOI) so that it can be cited independently in the future. For instructions see: https://journals.plos.org/plosone/s/submission-guidelines#loc-laboratory-protocols. Additionally, PLOS ONE offers an option for publishing peer-reviewed Lab Protocol articles, which describe protocols hosted on protocols.io. Read more information on sharing protocols at https://plos.org/protocols?utm_medium=editorial-email&utm_source=authorletters&utm_campaign=protocols.

As the corresponding author, your ORCID iD is verified in the submission system and will appear in the published article. PLOS supports the use of ORCID, and we encourage all coauthors to register for an ORCID iD and use it as well. Please encourage your coauthors to verify their ORCID iD within the submission system before final acceptance, as unverified ORCID iDs will not appear in the published article. *Only*  the individual author can complete the verification step; PLOS staff *cannot*  verify ORCID iDs on behalf of authors.

We look forward to receiving your revised manuscript.

Kind regards,

Peng Zhang, Ph.D.

Academic Editor

PLOS One

Journal Requirements:

1. If the reviewer comments include a recommendation to cite specific previously published works, please review and evaluate these publications to determine whether they are relevant and should be cited. There is no requirement to cite these works unless the editor has indicated otherwise.

2. Please review your reference list to ensure that it is complete and correct. If you have cited papers that have been retracted, please include the rationale for doing so in the manuscript text, or remove these references and replace them with relevant current references. Any changes to the reference list should be mentioned in the rebuttal letter that accompanies your revised manuscript. If you need to cite a retracted article, indicate the article’s retracted status in the References list and also include a citation and full reference for the retraction notice.

[Note: HTML markup is below. Please do not edit.]

Reviewers' comments:

Reviewer's Responses to Questions

**Comments to the Author**

Reviewer #1: All comments have been addressed

Reviewer #2: (No Response)

**********

2. Is the manuscript technically sound, and do the data support the conclusions?

Reviewer #1: Yes

Reviewer #2: Yes

**********

3. Has the statistical analysis been performed appropriately and rigorously?

Reviewer #1: Yes

Reviewer #2: Yes

**********

4. Have the authors made all data underlying the findings in their manuscript fully available?

The PLOS Data policy

Reviewer #1: Yes

Reviewer #2: (No Response)

**********

5. Is the manuscript presented in an intelligible fashion and written in standard English?

Reviewer #1: Yes

Reviewer #2: Yes

**********

Reviewer #1: Having carefully reviewed this revised manuscript and the authors’ responses to the previous reviewers, I commend the authors for their thorough and thoughtful revisions. They have substantially improved conceptual clarity (especially regarding workplace objectification vs. organizational dehumanization), strengthened methodological transparency, and toned down overclaims. The two-study design (multi-wave survey + scenario experiment) provides convergent evidence for the proposed moderated mediation model. However, to further strengthen the manuscript prior to final acceptance, I offer the following comments for the authors to address in a final revision.

1. While Study 2 successfully manipulates PAC and tests its effect on WO (H2a) and the moderation by AAV (H3a), the full mediating pathway (PAC → WO → SVCB) and the moderated mediation (H3b) are only tested in Study 1’s correlational design. The authors acknowledge this limitation but do not attempt any supplementary causal mediation analysis (e.g., sensitivity analysis for potential unmeasured confounding) nor do they explicitly state that the full mediation model remains correlational.

2. AAV is measured at Time 1 in Study 1 and before manipulation in Study 2, which is appropriate. However, the authors do not test whether AAV itself might be influenced by prior PAC experiences (i.e., reverse causality or reciprocal dynamics). At minimum, discuss this as a limitation: “AAV was treated as a stable individual difference, but longitudinal designs are needed to rule out that prior PAC experiences may shape AAV over time.”

Reviewer #2: Well done to the authors for addressing the comments in previous rounds of reviews.

There are a few minor comments left:

---

Lines 90-93: It's not clear what theory, evidence (or past research) the authors are drawing from to state that: "Therefore, compared with the broader concept of organizational dehumanization, WO is better able to capture platform workers’ perceptions of instrumentality and replaceability under algorithmic control."

As in previous rounds of revisions, we suggest authors be careful about explicitly pitting organizational dehumanization against WO; unless, of course, there is strong backing from previous work in this case.

Lines 264 to 276: It seems like the same argument is being made and discussed twice in a row: "AAV is likely to be activated by two key situational cues " (First on line 264 and next on line 270) But the two cues that are listed each time are a bit different, though they both include 'unpredictable algorithmic cues" as one of them. The discussion here could be tightened a little to avoid the repetition.

Lines 676-690 and lines 705-724: These two paragraphs, that begin with "Third, this study further clarifies", seem to be repeating the same content. It looks like paragraph that discussed point "Two" may have been taken out by mistake and replaced with a repeated point "Three"?

**********

what does this mean?). If published, this will include your full peer review and any attached files.

If you choose “no”, your identity will remain anonymous but your review may still be made public.

**Do you want your identity to be public for this peer review?**  For information about this choice, including consent withdrawal, please see our Privacy Policy

Reviewer #1: No

Reviewer #2: No

**********

[NOTE: If reviewer comments were submitted as an attachment file, they will be attached to this email and accessible via the submission site. Please log into your account, locate the manuscript record, and check for the action link "View Attachments". If this link does not appear, there are no attachment files.]

To ensure your figures meet our technical requirements, please review our figure guidelines: https://journals.plos.org/plosone/s/figures

You may also use PLOS’s free figure tool, NAAS, to help you prepare publication quality figures: https://journals.plos.org/plosone/s/figures#loc-tools-for-figure-preparation.

NAAS will assess whether your figures meet our technical requirements by comparing each figure against our figure specifications.

# Author comment: Author response to Decision Letter 3

**DOI:** 10.1371/journal.pone.0357097.r006

27 Jul 2026

Response to reviewers

Reviewer #1: Having carefully reviewed this revised manuscript and the authors’ responses to the previous reviewers, I commend the authors for their thorough and thoughtful revisions. They have substantially improved conceptual clarity (especially regarding workplace objectification vs. organizational dehumanization), strengthened methodological transparency, and toned down overclaims. The two-study design (multi-wave survey + scenario experiment) provides convergent evidence for the proposed moderated mediation model. However, to further strengthen the manuscript prior to final acceptance, I offer the following comments for the authors to address in a final revision.

mini

Comments to the author:

1.While Study 2 successfully manipulates PAC and tests its effect on WO (H2a) and the moderation by AAV (H3a), the full mediating pathway (PAC → WO → SVCB) and the moderated mediation (H3b) are only tested in Study 1’s correlational design. The authors acknowledge this limitation but do not attempt any supplementary causal mediation analysis (e.g., sensitivity analysis for potential unmeasured confounding) nor do they explicitly state that the full mediation model remains correlational.

Authors’ response:

We would like to once again express our sincere gratitude for the reviewer's thorough review and constructive comments on our manuscript. Regarding the first concern—that the full mediation model is based on correlational data and should be explicitly stated as such—we have made targeted additions in the General Discussion section (see the revised manuscript, with the added text highlighted in yellow):

"It should be noted, however, that the full mediation model (PAC → WO → SVCB) and the moderated mediation model (H3b) were tested using correlational data from Study 1. Although Study 2 provided experimental evidence for the effect of PAC on WO and the moderating role of AAV, the complete mediating pathway and the moderated mediation effect remain correlational in nature and should not be interpreted as establishing strict causal ordering."

Here, we would like to further clarify the methodological logic of our two-study design. The purpose of our two-study design is not to attempt to establish all causal relationships with a single study; rather, through a progressive logic of "correlational → experimental," we leverage the distinct strengths of each study: Study 1 tests the full model in real platform work contexts, providing external validity—demonstrating that the theoretical model holds in real-world settings; Study 2, through experimental manipulation, provides internal validity for the PAC → WO relationship—establishing the causal nature of this key link. The full model indeed remains correlational in nature, but Study 2 provides causal evidence for the first half of the mediating pathway (PAC → WO), which strengthens our confidence in the overall model and clearly points to the direction for future research to further examine the complete causal chain.

Once again, we thank the reviewer for the rigorous scrutiny; these comments have greatly improved the accuracy and academic rigor of our methodological discussion.

Comments to the author:

2. AAV is measured at Time 1 in Study 1 and before manipulation in Study 2, which is appropriate. However, the authors do not test whether AAV itself might be influenced by prior PAC experiences (i.e., reverse causality or reciprocal dynamics). At minimum, discuss this as a limitation: “AAV was treated as a stable individual difference, but longitudinal designs are needed to rule out that prior PAC experiences may shape AAV over time.”

Authors’ response:

Regarding the discussion of the limitation that AAV was treated as a stable individual difference, we agree with the reviewer's viewpoint. Although measuring AAV at Time 1 in Study 1 and before the manipulation in Study 2 is methodologically reasonable in terms of temporal precedence, we acknowledge that this study indeed did not examine whether AAV itself might be influenced by prior PAC experiences. As the reviewer rightly pointed out, while AAV was treated as a stable individual difference, prior PAC experiences may potentially shape individuals' AAV levels over time, thereby giving rise to reverse causality or reciprocal dynamics.

In accordance with the reviewer's suggestion, we have supplemented the relevant content in the limitations section of the revised manuscript (lines 895-900), with the added text highlighted in yellow. The supplementary content reads as follows:

"Furthermore, although measuring AAV at Time 1 in Study 1 and before the manipulation in Study 2 is methodologically reasonable in terms of temporal precedence, this study did not examine whether AAV itself might be influenced by prior PAC experiences. Future research should adopt longitudinal designs to rule out the possibility that prior PAC experiences may shape AAV over time, thereby further clarifying the causal direction between the two variables."

This addition makes the limitations discussion of this study more comprehensive and rigorous, and also points to a promising direction for future research to further explore.

Once again, we thank the reviewer for the valuable suggestions, which have significantly enhanced the academic rigor of our manuscript.

Reviewer #2: Well done to the authors for addressing the comments in previous rounds of reviews. There are a few minor comments left:

Major Comments:

Comments to the author:

Lines 90-93: It's not clear what theory, evidence (or past research) the authors are drawing from to state that: "Therefore, compared with the broader concept of organizational dehumanization, WO is better able to capture platform workers’ perceptions of instrumentality and replaceability under algorithmic control." As in previous rounds of revisions, we suggest authors be careful about explicitly pitting organizational dehumanization against WO; unless, of course, there is strong backing from previous work in this case. Lines 264 to 276: It seems like the same argument is being made and discussed twice in a row: "AAV is likely to be activated by two key situational cues " (First on line 264 and next on line 270) But the two cues that are listed each time are a bit different, though they both include 'unpredictable algorithmic cues" as one of them. The discussion here could be tightened a little to avoid the repetition. Lines 676-690 and lines 705-724: These two paragraphs, that begin with "Third, this study further clarifies", seem to be repeating the same content. It looks like paragraph that discussed point "Two" may have been taken out by mistake and replaced with a repeated point "Three"?

Authors’ response:

We sincerely thank the reviewer for the careful review and valuable comments on our manuscript. Regarding the concern about the lack of clear theoretical or empirical basis for the statement in lines 90-93, we have carefully reflected on this issue and made corresponding revisions. Our detailed responses are as follows:

On the issue of the statement that "WO is better able to capture platform workers' perceptions" (lines 90-93):

We greatly appreciate the reviewer's prudent reminder. We fully accept the suggestion. We have revised the sentence as follows:

Original sentence: "Therefore, compared with the broader concept of organizational dehumanization, WO is better able to capture platform workers' perceptions of instrumentality and replaceability under algorithmic control."

Revised sentence: "Therefore, focusing on the research context of this study, WO is better able to capture platform workers' perceptions of instrumentality and replaceability under algorithmic control."

The revised wording shifts the focus of the argument from "comparison between constructs" to "fit with the research context," thereby preserving the logical coherence of the original argument while effectively avoiding any comparative or oppositional interpretation that might arise from explicitly mentioning organizational dehumanization.

On the issue of redundant discussion in lines 264-276:

In accordance with the reviewer's suggestion, we have thoroughly reorganized and rewritten this paragraph (now located near line 267), removing redundant statements. The specific revisions have been highlighted in yellow at line 267 of the revised manuscript for the reviewer's reference. The new formulation avoids the mechanical, itemized listing of the previous version, making the argumentation more concise and focused.

On the issue of content repetition between lines 676-690 and lines 705-724:

The reviewer correctly pointed out that lines 676-690 and lines 705-724 indeed repeated the same "Third" point. Upon careful verification of the revision process, we confirmed that this was due to an operational error during the adjustment of paragraph structure in the previous round of revision, which resulted in the unintended deletion of the paragraph discussing the "Second" point, while the redundant "Third" point was retained. Accordingly, we have thoroughly corrected this section as follows: (1) The redundant paragraph at lines 705-724 has been deleted. (2) The content at lines 676-690 has been rewritten to serve as a complete discussion of the "Second" point, with the substantive content regarding the study's theoretical contributions that was originally omitted now supplemented.

We sincerely apologize for the inconvenience caused by this editorial oversight during the reviewer's evaluation, and we are grateful for the reviewer's rigorous scrutiny. The theoretical contributions of this section are now presented in a clear and logically structured manner.

Once again, we thank the reviewer for the valuable suggestions, which have significantly enhanced the quality of our manuscript. We hope that our revisions meet the reviewer's expectations.

Attachment Submitted filename: Response_to_reviewers_auresp_3.docx

# Peer review: Decision Letter 3

**DOI:** 10.1371/journal.pone.0357097.r007

**Authors:** Peng Zhang

13 Aug 2026

How to break free from the "tool" dilemma? A study on the impact of perceived algorithmic control on sustainable value co-creation behavior

PONE-D-25-44146R3

Dear Dr. Wang,

We’re pleased to inform you that your manuscript has been judged scientifically suitable for publication and will be formally accepted for publication once it meets all outstanding technical requirements.

Within one week, you’ll receive an e-mail detailing the required amendments. When these have been addressed, you’ll receive a formal acceptance letter and your manuscript will be scheduled for publication.

An invoice will be generated when your article is formally accepted. Please note, if your institution has a publishing partnership with PLOS and your article meets the relevant criteria, all or part of your publication costs will be covered. Please make sure your user information is up-to-date by logging into Editorial Manager at Editorial Manager® and clicking the ‘Update My Information' link at the top of the page. For questions related to billing, please contact billing support.

If your institution or institutions have a press office, please notify them about your upcoming paper to help maximize its impact. If they’ll be preparing press materials, please inform our press team as soon as possible -- no later than 48 hours after receiving the formal acceptance. Your manuscript will remain under strict press embargo until 2 pm Eastern Time on the date of publication. For more information, please contact onepress@plos.org.

Kind regards,

Peng Zhang, Ph.D.

Academic Editor

PLOS One

Additional Editor Comments (optional):

Reviewers' comments:

Reviewer's Responses to Questions

**Comments to the Author**

Reviewer #1: All comments have been addressed

Reviewer #2: All comments have been addressed

**********

2. Is the manuscript technically sound, and do the data support the conclusions?

Reviewer #1: (No Response)

Reviewer #2: Yes

**********

3. Has the statistical analysis been performed appropriately and rigorously?

Reviewer #1: (No Response)

Reviewer #2: I Don't Know

**********

4. Have the authors made all data underlying the findings in their manuscript fully available?

The PLOS Data policy

Reviewer #1: (No Response)

Reviewer #2: Yes

**********

5. Is the manuscript presented in an intelligible fashion and written in standard English?

Reviewer #1: (No Response)

Reviewer #2: Yes

**********

Reviewer #1: (No Response)

Reviewer #2: We commend the authors on carefully addressing the revisions suggested throughout the review process. At this point, we believe the article is ready to be accepted.

**********

what does this mean?). If published, this will include your full peer review and any attached files.

If you choose “no”, your identity will remain anonymous but your review may still be made public.

**Do you want your identity to be public for this peer review?**  For information about this choice, including consent withdrawal, please see our Privacy Policy

Reviewer #1: No

Reviewer #2: No

**********

# Editor report: Acceptance letter

**DOI:** 10.1371/journal.pone.0357097.r008

**Authors:** Peng Zhang

PONE-D-25-44146R3

PLOS One

Dear Dr. Wang,

I'm pleased to inform you that your manuscript has been deemed suitable for publication in PLOS One. Congratulations! Your manuscript is now being handed over to our production team.

At this stage, our production department will prepare your paper for publication. This includes ensuring the following:

* All references, tables, and figures are properly cited

* All relevant supporting information is included in the manuscript submission,

* There are no issues that prevent the paper from being properly typeset

You will receive further instructions from the production team, including instructions on how to review your proof when it is ready. Please keep in mind that we are working through a large volume of accepted articles, so please give us a few days to review your paper and let you know the next and final steps.

Lastly, if your institution or institutions have a press office, please let them know about your upcoming paper now to help maximize its impact. If they'll be preparing press materials, please inform our press team within the next 48 hours. Your manuscript will remain under strict press embargo until 2 pm Eastern Time on the date of publication. For more information, please contact onepress@plos.org.

You will receive an invoice from PLOS for your publication fee after your manuscript has reached the completed accept phase. If you receive an email requesting payment before acceptance or for any other service, this may be a phishing scheme. Learn how to identify phishing emails and protect your accounts at https://explore.plos.org/phishing.

If we can help with anything else, please email us at customercare@plos.org.

Thank you for submitting your work to PLOS One and supporting open access.

Kind regards,

PLOS One Editorial Office Staff

on behalf of

Prof. Peng Zhang

Academic Editor

PLOS One
